# Cancer Cell Mitochondria Targeting by Pancratistatin Analogs is Dependent on Functional Complex II and III

**DOI:** 10.1038/srep42957

**Published:** 2017-02-21

**Authors:** Dennis Ma, Christopher Pignanelli, Daniel Tarade, Tyler Gilbert, Megan Noel, Fadi Mansour, Scott Adams, Alexander Dowhayko, Kyle Stokes, Sergey Vshyvenko, Tomas Hudlicky, James McNulty, Siyaram Pandey

**Affiliations:** 1 Department of Chemistry and Biochemistry, University of Windsor, 401 Sunset Avenue, Windsor, Ontario N9B 3P4, Canada; 2 Chemistry Department and Centre for Biotechnology, Brock University, 500 Glenridge Avenue, St. Catharines, Ontario L2S 3A1, Canada; 3 Department of Chemistry, McMaster University, 1280 Main Street West, Hamilton, Ontario L8S 4M1, Canada

## Abstract

Enhanced mitochondrial stability and decreased dependence on oxidative phosphorylation confer an acquired resistance to apoptosis in cancer cells, but may present opportunities for therapeutic intervention. The compound pancratistatin (PST) has been shown to selectively induce apoptosis in cancer cells. However, its low availability in nature has hindered its clinical advancement. We synthesized PST analogs and a medium-throughput screen was completed. Analogs SVTH-7, -6, and -5 demonstrated potent anti-cancer activity greater than PST and several standard chemotherapeutics. They disrupted mitochondrial function, activated the intrinsic apoptotic pathway, and reduced growth of tumor xenografts *in vivo*. Interestingly, the pro-apoptotic effects of SVTH-7 on cancer cells and mitochondria were abrogated with the inhibition of mitochondrial complex II and III, suggesting mitochondrial or metabolic vulnerabilities may be exploited by this analog. This work provides a scaffold for characterizing distinct mitochondrial and metabolic features of cancer cells and reveals several lead compounds with high therapeutic potential.

Mitochondria serve many cellular functions including energy production via oxidative phosphorylation. Additionally, this organelle plays a pivotal role in apoptosis, a cellular suicide program, housing apoptogenic factors that lead to the detriment of the cell once released into the cytoplasm^1^. Unlike the extrinsic pathway of apoptosis, which requires an external stimulus, the intrinsic pathway is elicited by internal stress, such as DNA damage or oxidative stress^2^. Following intracellular stress, mitochondrial permeabilization is induced, causing apoptogenic factor release, and subsequent execution of apoptosis. This process can occur physiologically, during embryogenesis, and acts as a safeguard against DNA damage, but can also be pathologically altered in various disease states^3^,^4^. Cancer cells divert most of their means of energy production to glycolysis and away from mitochondrial oxidative phosphorylation^5^. This reallocation in cellular energetics, in addition to the upregulation of anti-apoptotic proteins, provides additional stability to mitochondria and, consequently, an acquired resistance to apoptosis^6^,^7^,^8^. Thus, targeting the differences in energy metabolism and cancer cell mitochondria could serve as potential strategies for cancer therapy^9^.

Mitocans are a class of compounds that have recently emerged and are defined as drugs that specifically target cancer cell mitochondria to induce mitochondrial dysfunction and activation of mitochondrial mediated apoptosis pathways^10^,^11^. These include, but are not limited to, electron transport chain (ETC) blockers, activators of the permeability transition pore of the mitochondria, pro-apoptotic Bcl-2 protein mimetics, and anti-apoptotic Bcl-2 protein inhibitors^12^,^13^,^14^,^15^. Mitocans have been shown to be generally well tolerated by non-cancerous cells and effective anti-cancer agents, alone or in combination to enhance the activity of other cancer therapies^16^.

We have previously discovered one such compound, pancratistatin (PST), an Amaryllidaceae alkaloid isolated from the *Hymenocallis littoralis* plant, which selectively induces apoptosis in numerous cancer cell types by mitochondrial targeting^17^,^18^,^19^,^20^,^21^,^22^,^23^. However, further development of PST has been hindered by its low availability in the *Hymenocallis littoralis* species and complications in its chemical synthesis. Circumventing these bottlenecks, we have synthesized a number of PST analogs that possess the proposed anti-cancer pharmacophore of PST and related alkaloids^24^.

In this study, the anti-cancer activity of 7-deoxyPST and PST analogs, natural PST, and standard chemotherapeutics were evaluated via a medium-throughput screen in an array of cancer cell lines and non-cancerous cells. Several PST analogs, including SVTH-7, -6, and -5 demonstrated selective, potent anti-cancer activity, having greater efficacy than natural PST, their C-7 deoxy counterparts, and, most importantly, several standard chemotherapeutics. These analogs were effective in disrupting mitochondrial function and activating the intrinsic pathway of apoptosis. Furthermore, these analogs were able to induce apoptosis of cancer cells grown in three-dimensional spheroid culture selectively and reduce growth of colorectal cancer and glioblastoma tumor xenografts *in vivo*. Interestingly, inhibition of mitochondrial complex II and III abolished the mitochondrial pro-apoptotic effects of SVTH-7, suggesting that a mitochondrial vulnerability may be exploited by this PST analog. This novel observation forms a basis for discerning important mitochondrial and metabolic features in cancer cells and presents several compounds with high therapeutic potential.

## Results

### PST Analogs have Selective Anti-Cancer Activity Greater Than Standard Chemotherapeutics & Natural PST

**Figure 1:**
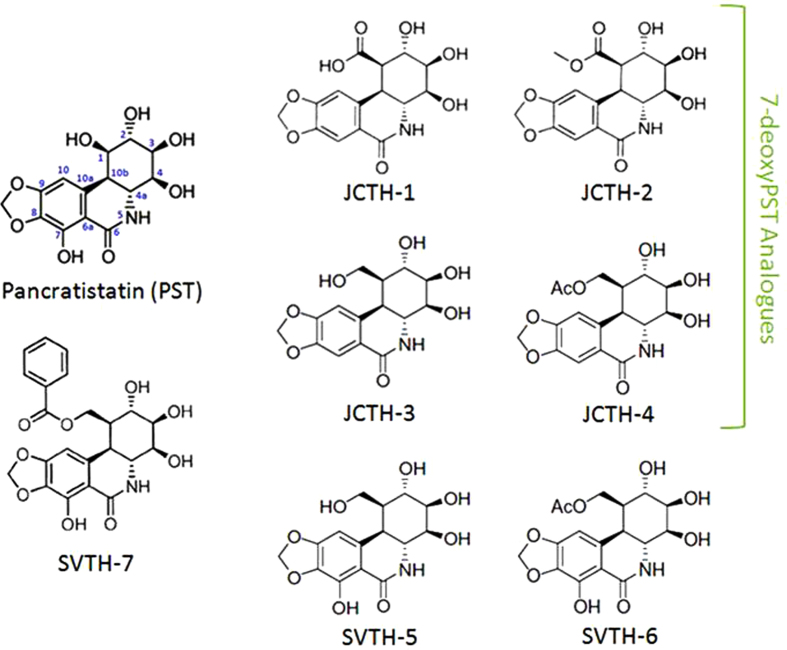
Structure of PST and PST Analogs.

**Figure 2:**
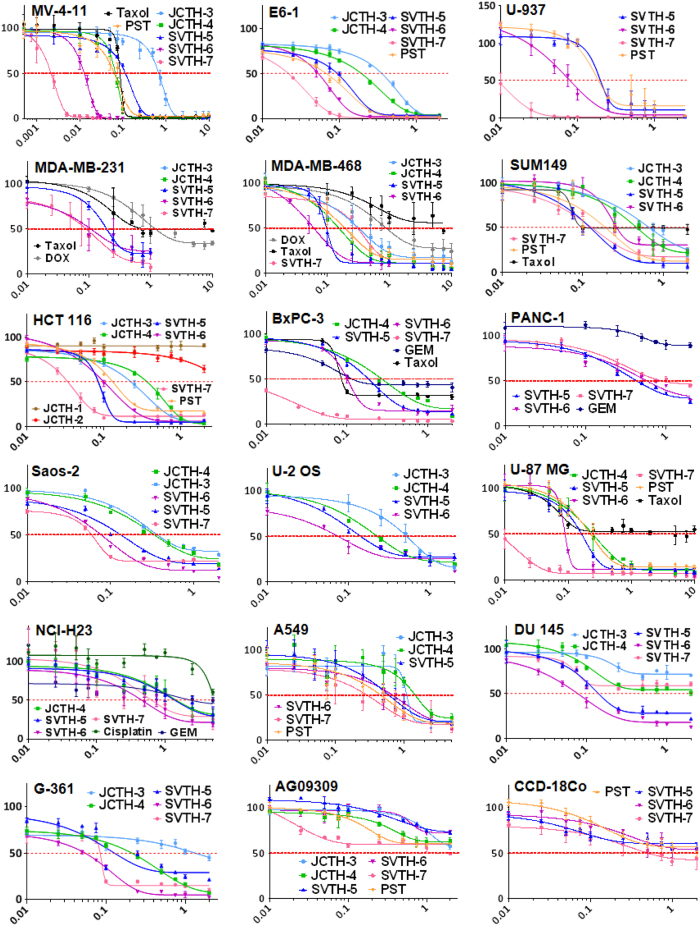
PST Analogs have Selective Anti-Cancer Activity Greater Than Standard Chemotherapeutics & Natural PST. Cancer and non-cancerous cells were treated with PST analogs, PST, Taxol, Doxorubicin (DOX), Gemcitabine (GEM), and Cisplatin for 48 hours. With the WST-1 assay, absorbance was read at 450 nm and expressed as a percent of control (DMSO). Values are expressed as mean ± SD from quadruplicates of 3 independent experiments. X-axis: Concentration (μM) (using a Log 10 Scale). Y-axis: Absorbance at 450 nm (% of Control).

The preclinical advancement of PST has been hindered by its low yield from its natural source and complexities in its chemical synthesis. Previously, we have shown modest to comparable anti-cancer activity of a 7-deoxyPST analog in comparison to natural PST^25^,^26^,^27^. Recently, we have synthesized several PST analogs with a C-7 hydroxyl group (Fig. 1), thus, possessing the complete pharmacophore attributed to the anticancer activity of PST and related alkaloids^24^. A comprehensive screen of anti-cancer activity of these analogs, in parallel with 7-deoxyPST analogs, PST, and common chemotherapeutics was completed on a battery of cancer cell lines as well as non-cancerous cells using the WST-1 colorimetric assay (Fig. 2). Taken as a whole, SVTH-7, followed by SVTH-6 and SVTH-5, had the most potent activity, with SVTH-7 having much greater activity than natural PST while SVTH-6 and -5 possessed comparable or greater efficacy than natural PST with regards to their half-maximal inhibitory concentration (IC_50_) values (Table 1). As predicted, SVTH-6 and SVTH-5, which are C-7 hydoxylated forms of JCTH-4 and JCTH-3, respectively, were markedly more effective than their 7-deoxyPST counterparts.

Triple negative breast cancer (TNBC) lacks the estrogen, progesterone, and HER2 receptor, and thus traditional breast cancer therapies, including hormone therapy and Herceptin, are not effective^28^. Standard chemotherapeutics for TNBC include Taxol and Doxorubicin (DOX). Interestingly, SVTH-7, -6, and -5 had lower IC_50_ values than Taxol and DOX in the TNBC cell lines MDA-MB-231 and MDA-MB-468. SVTH-7 and SVTH-6 were also more effective than Gemcitabine (GEM), the standard chemotherapeutic for notoriously chemoresistant pancreatic cancer^29^, in the BxPC-3 and PANC-1 pancreatic cancer cell lines. Furthermore, JCTH-3 and -4, and SVTH-5, -6, and -7 were more potent than Cisplatin and GEM, having lower IC_50_ values in the NCI-H23 non-small cell lung cancer cell line, a commonly chemoresistant cancer. Moreover, SVTH-7 and -6 had lower IC_50_ values than Taxol in MV-4-11 leukemia, and U-87 MG glioblastoma. Similar results were observed with MCF7 breast adenocarcinoma, OVCAR-3 ovarian adenocarcinoma, and Hep G2 hepatoma cells (Supplemental Fig. 1a). Notably, the IC_50_ values of PST and its analogs in the AG09309 and CCD-18Co non-cancerous cells were well above those observed in the cancer cells lines tested, demonstrating a selective therapeutic window. Additional time points, doses and statistical analyses of compounds tested are shown in Supplemental Fig. 1b–w.

### PST Analogs Induce Apoptosis Selectively in Cancer Cells

**Figure 3:**
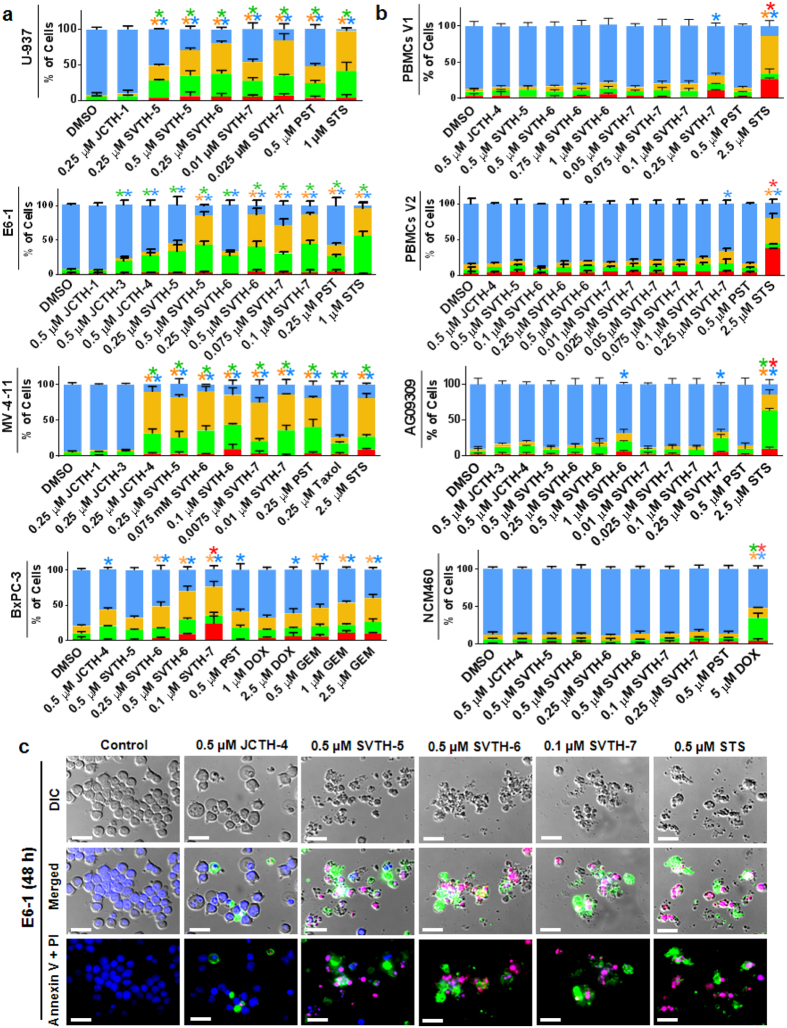
PST Analogs Induce Apoptosis Selectively in Cancer Cells. Annexin V binding and PI staining of cells treated for 48 hours was monitored with image-based cytometry. **(a)** Cancer cell lines. **(b)** Non-cancerous cells. Values are expressed as mean ± SD from at least 3 independent experiments. *p < 0.01 vs. DMSO control. **(c)** Annexin V binding (green), PI staining (red), and Hoechst (blue) monitored with microscopy. Cell morphology is shown using differential interference contrast (DIC) microscopy. Scale bar = 25 μm. Micrographs are representative of 3 independent experiments.

To evaluate cell death caused by PST analogs, the Annexin V binding assay and propidium iodide (PI) staining was done in parallel to monitor early apoptosis^30^ and necrotic or late apoptotic cell death, respectively^31^. PST analogs were effective in inducing apoptosis in the U-937, E6-1, and MV-4-11 lymphoma and leukemia cell lines as well as BxPC-3 pancreatic adenocarcinoma cells (Fig. 3a). SVTH-7, followed by SVTH-6 and SVTH-5, was the most effective in inducing apoptosis compared to natural PST and the 7-deoxyPST analogs. Staurosporine (STS) was used as a positive control for apoptotic induction^32^. Interestingly, SVTH-6 and -7 were more efficacious in inducing apoptosis in BxPC-3 cells compared to Gemcitabine (GEM), the standard chemotherapeutic for pancreatic cancer. Non-cancerous cells, including peripheral blood mononuclear cells from healthy volunteers 1 (PBMCs V1) and 2 (PBMCs V2), AG09309 normal human fibroblasts, and NCM460 normal human epithelial cells were much less sensitive to apoptotic induction. Only SVTH-7 and SVTH-6, at doses substantially higher than what is required to induce apoptosis in cancer cells, demonstrated mild toxicity in some of these non-cancerous cells (Fig. 3b). Cell death analyses of additional non-cancerous peripheral blood mononuclear cells from other healthy volunteers with similar resilience against PST analog treatment are depicted in Supplemental Fig. 2a. Furthermore, to see if apoptosis could occur in actively dividing PBMCs V1, concanavalin a (Con A), a known inducer of proliferation of peripheral blood mononuclear cells was added. Although Con A was able to induce proliferation, PST and PST analogs were still not able to trigger apoptosis in PBMCs V1, which is in contrast with Taxol treatment, indicating that PST and its analogs do not target cells because they are actively dividing (Supplemental Fig. 2b). HEK-293 human embryonic kidney cells were also unresponsive to PST and PST analog treatment (Supplemental Fig. 2c). Representative micrographs of E6-1 leukemia cells undergoing apoptosis after 48 hours of PST analog treatment are shown in Fig. 3c. SVTH-7, -6, and -5 were the most effective at yielding condensed cell morphology, nuclear condensation, and Annexin V (green) and PI (red) fluorescence, which are indicative of apoptotic induction.

### PST Analogs Activate the Intrinsic Pathway of Apoptosis in Cancer Cells

**Figure 4:**
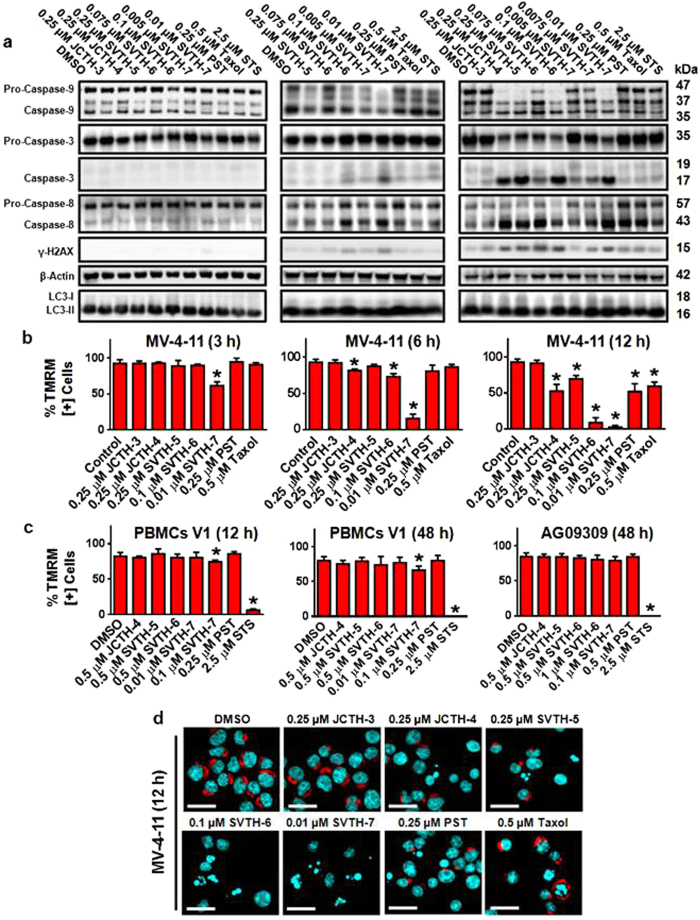
PST Analogs Activate the Intrinsic Pathway of Apoptosis in Cancer Cells. **(a)** Western blot analysis of cell lysates of MV-4-11 Leukemia cells treated with PST, PST Analogs, Taxol, and staurosporine (STS) for 3, 6 and 12 hours. TMRM was used to monitor MMP in **(b)** MV-4-11 leukemia cells, **(c)** non-cancerous peripheral blood mononuclear cells from volunteer 1 (PBMCs V1), and normal human fibroblasts (AG09309) with image-based cytometry. *p < 0.01 vs. DMSO control. **(d)** TMRM fluorescence microscopy counterstained with Hoechst (cyan). Scale bar = 25 μm. All images are representative of at least 3 independent experiments.

Mitochondria play a pivotal role in the induction of intrinsic apoptosis. When dysfunctional, these organelles can permeabilize and release apoptogenic factors, leading to the execution of apoptosis^33^. One such factor is cytochrome c (Cyto c), which upon its release from the mitochondria, leads to the conversion of Pro-Caspase-9 (Pro-Casp-9) to Caspase-9 (Casp-9), which in turn cleaves Pro-Caspase-3 (Pro-Casp-3) to Caspase-3 (Casp-3)^34^. The executioner caspase, Casp-3, exerts its lethal effects by cleaving a multitude of cellular proteins needed for cellular function, structural stability, and survival^35^. MV-4-11 leukemia cells were treated with PST and PST analogs and no noticeable activation of caspases were observed at 3 hours. At 6 hours, there is prominent activation of Casp-9 and -3 with SVTH-7 and to a lesser extent with SVTH-6. After 12 hours, JCTH-4, SVTH-5, -6, and -7 treatment yielded prominent cleavage of Pro-Casp-9 and Pro-Casp-3, as well as DNA damage, as indicated by the presence of γ-H2AX, in MV-4-11 cells (Fig. 4a), demonstrating their ability to induce the aforementioned caspase-dependent pathway of apoptosis. Densitometric analyses are depicted in Supplemental Fig. 3a. Moreover, SVTH-7 caused the release of Cyto c from the mitochondria of MV-4-11 cells (Supplemental Fig. 3b). Although at 12 hours there is activation of Caspase-8, leukemia cells that are dominant negative for the Fas-Associated Death Domain (FADD) (DN FADD Jurkat), a critical component of the extrinsic pathway and activation of Caspase-8, were still very sensitive to PST and PST analogs compared to corresponding leukemia cells with functional FADD (Supplemental Fig. 3c). This indicates that the extrinsic pathway of apoptosis is not a predominant pathway responsible for PST and PST-analog-induced cytotoxicity. Likewise, there was no observable differences in the conversion of LC3-I to LC3-II, a marker of autophagy^36^, with PST and PST analog treatment, suggesting autophagy to possess little role in the anti-cancer activity of these compounds (Fig. 4a and Supplemental Fig. 3b).

Complimenting these findings, permeabilization of mitochondria, as seen with dissipation of mitochondrial membrane potential (MMP), was first noticeable at 3 hours in MV-4-11 cells with SVTH-7 treatment, and at 6 hours with JCTH-4 and SVTH-6, with a more pronounced effect at 12 hours as shown by a decrease of TMRM red fluorescence (Fig. 4b). Additional time points and doses are shown in (Supplemental Fig. 3d) and similar results are observed with E6-1 leukemia and BxPC-3 pancreatic adenocarcinoma cells (Supplemental Fig. 3e and f). This effect on mitochondria was observed to be selective towards cancer cells as PMBCs V1 and AG09309 had very minimal or no observable decreases in MMP with PST and PST analog treatment (Fig. 4c). Representative micrographs depicting TMRM fluorescence of MV-4-11 cells treated with PST and PST analog, Taxol, and staurosporine (STS) are shown in Fig. 4d. Collectively, these findings suggest that PST analogs are able to act on the mitochondria to induce the intrinsic pathway of apoptosis.

### PST and PST-Induced Apoptosis is Highly Dependent on Mitochondrial Membrane Permeabilization and Partially Dependent on Caspase Activity

**Figure 5:**
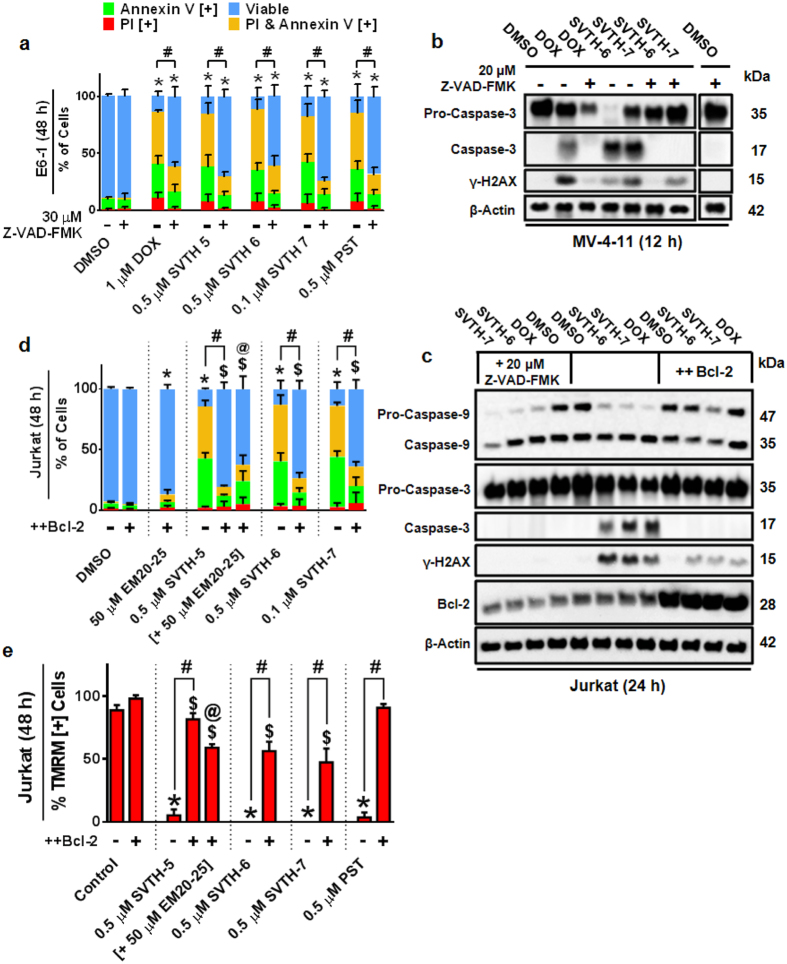
PST and PST-Induced Apoptosis is Highly Dependent on Mitochondrial Membrane Permeabilization and Partially Dependent on Caspase Activity. (**a**) E6-1 cells were pre-treated with Z-VAD-FMK caspase inhibitor for 1 hour and then treated with PST analogs and DOX. Annexin V binding and PI staining was quantified with image based cytometry. *p < 0.01 vs. DMSO control (comparison of viable cells only); ^#^*p* < 0.001 vs. respective groups untreated with Z-VAD-FMK (comparison of viable cells only). (**b**) Western blot analysis was performed on MV-4-11 cells pre-treated with Z-VAD-FMK for one hour and treated with 1 μM DOX, 0.5 μM SVTH-6, and 0.01 μM SVTH-7 for an additional 12 hours. (**c**) Jurkat cells (E6-1 leukemia cells) were pre-treated with Z-VAD-FMK for 1 hour or DMSO. These cells, along with Jurkat cells over-expressing the anti-apoptotic protein Bcl-2 (++Bcl-2), were then treated with 1 μM DOX, 0.5 μM SVTH-6, and 0.1 μM SVTH-7 for 24 hours. Western blot analysis was performed on corresponding cell lysates. (**d**) Annexin V binding and PI staining, as well as (**e**) TMRM was quantified with image based cytometry on these Jurkat after 48 hours of treatment. *p < 0.01 vs. DMSO control (comparison of viable cells only); ^$^*p* < 0.01 vs. DMSO control of ++Bcl-2 Jurkat; ^#^*p* < 0.001 vs. respective groups without over-expression of Bcl-2; @*p* < 0.01 vs. ++Bcl-2 Jurkat treated with 0.5 μM SVTH-5 alone. All quantitative values are expressed as mean ± SD from at least 3 independent experiments. Western blots are representative of at least 3 independent experiments.

Following MMP collapse and mitochondrial permeabilization, apoptogenic factors are released and cause subsequent activation of caspases. To study the dependence of caspases in PST analog-induced apoptosis, the Z-VAD-FMK broad-spectrum caspase inhibitor was used (Fig. 5a). Interestingly, this inhibitor was able to prevent activation of Casp-3 by SVTH-6 and -7 in MV-4-11 (Fig. 5b) and E6-1 (Fig. 5c) leukemia cells. Densitometric analyses are given in Supplemental Fig. 4a and b. This was able to partially rescue E6-1 and MV-4-11 (Supplemental Fig. 4c) leukemia cells from PST and PST analog-induced apoptosis, suggesting both caspases and other apoptosis inducers are involved in such cell death. Doxorubicin (DOX) was used as a positive control for Z-VAD-FMK-mediated rescue^37^.

Jurkat cells over-expressing the anti-apoptotic protein Bcl-2 (++Bcl-2), a protein known to stabilize mitochondria and prevent mitochondrial membrane permeabilization, were then treated with PST analogs and Doxorubicin (DOX) for 24 hours. ++Bcl-2 Jurkat cells had drastically lower levels of Casp-3 and -9 activation and γ-H2AX compared to Jurkat cells with no over-expression of Bcl-2 (E6-1) (Fig. 5c). Densitometric analyses are given in Supplemental Fig. 4b Furthermore, ++Bcl-2 Jurkat cells were drastically less susceptible to PST analog-induced apoptosis (Fig. 5d) and experienced significantly less MMP dissipation (Fig. 5e) compared to Jurkat without this over-expression of this mitochondrial stabilizing protein. Interestingly, the Bcl-2 inhibitor EM20-25, which disrupts interactions between Bcl-2 and Bax^38^, was able to sensitize the ++Bcl-2 Jurkat to the PST analog SVTH-5 and potentiated its ability to induce apoptosis and dissipate MMP in these cells (Fig. 5d and e). Therefore, this body of work suggests that PST analog-induced apoptosis is highly dependent on mitochondrial membrane permeabilization.

### PST Analogs Act on Cancer Cell Mitochondria and Cause Mitochondrial Dysfunction

**Figure 6:**
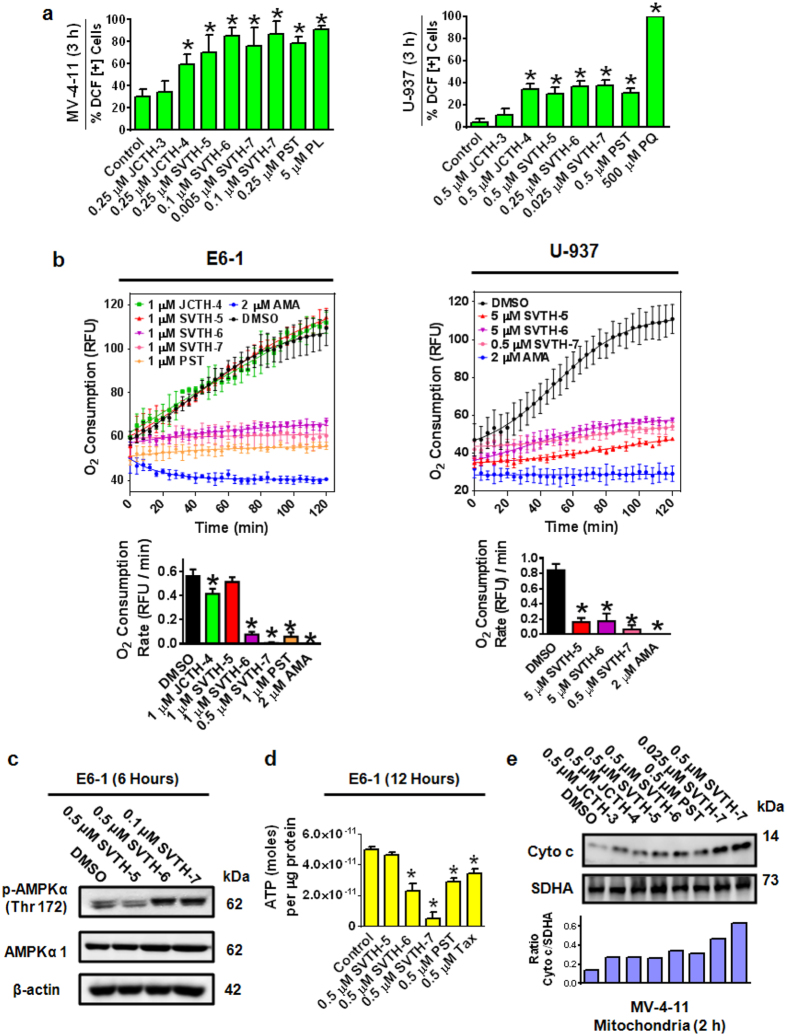
PST Analogs Act on Cancer Cell Mitochondria and Cause Mitochondrial Dysfunction. **(a)** H2DCFDA was used to measure whole cell ROS in MV-4-11 and U-937 cells treated for 3 hours with image-based cytometry. *p < 0.01 vs. DMSO control. **(b)** The MitoXpress^®^ Xtra - Oxygen Consumption Assay was used to monitor oxygen consumption via fluorescence generation. Cells were treated, and the fluorescent MitoXpress^®^ reagent was added and monitored at Ex. 380 nm and Em. 650, every 2 minutes for 2 hours at 37 °C. Oxygen consumption rates were calculated by measuring the slopes of the linear regions of the oxygen consumption curves. *p < 0.001 vs. DMSO control. **(c)** Western blot analysis of E6-1 following treatment with the indicated drugs for 6 hours. Results are representative of 3 independent trials. **(d)** Detection of ATP levels following treatment with SVTH-5, -6, and -7, PST, and Taxol using the luciferase-luciferin ATP determination assay. Amount of ATP was expressed as number of moles of ATP over micrograms of protein. Results are shown as the mean ± SD from at least 3 independent experiments. *p < 0.05 vs DMSO control. **(e)** Western Blot analysis of Cyto c release (of post mitochondrial supernatant) from directly treated mitochondria isolated from MV-4-11 cells for 2 hours. SDHA was probed in the mitochondrial pellet samples as loading controls. All quantitative values are expressed as mean ± SD from at least 3 independent experiments. Western blots are representative of at least 3 independent experiments.

One of the first events of mitochondrial dysfunction is the generation of reactive oxygen species (ROS)^39^. Using H_2_DCFDA, an indicator of ROS, PST analogs and PST were shown to increase the production of ROS in MV-4-11 leukemia and U-937 lymphoma cells after 3 hours of treatment (Fig. 6a). Piperlongumine (PL) and paraquat (PQ) were used as a positive control for ROS generation^40^,^41^.

Oxygen consumption of cells is a direct indicator of mitochondrial function^42^. To assess the effect of PST analogs on oxygen consumption, the MitoXpress^®^ Xtra - Oxygen Consumption Assay was used (Fig. 6b). SVTH-6, -7, and PST were able to effectively decrease the rate of oxygen consumption in E6-1 leukemia cells. In U-937 lymphoma cells, SVTH-5, -6, and -7 were effective in decreasing oxygen consumption rates. Antimycin A (AMA), a complex III inhibitor of the ETC, was used as a positive control for oxygen consumption cessation. Phosphorylation of AMPK (p-AMPK), a marker for activating several cell survival pathways, and total amount of ATP were measured following 6 and 12 hours of treatment and there was an increase in p-AMPK and a decrease in total amount of ATP in each group compared to the control, with the exception to SVTH-5 (Fig. 6c and d). These results indicate that PST analogs are effective in reducing oxygen consumption and therefore, mitochondrial function.

To determine if PST analogs are able to directly act on cancer cell mitochondria to release Cyto c, mitochondria isolated from MV-4-11 cells were directly treated with PST analogs for 2 hours and the release of Cyto c was monitored (Fig. 6e). Interestingly, such treatment caused the release of this apoptogenic factor with the most pronounced effect observed with SVTH-6 and -7. Therefore, together these findings demonstrate that PST and PST analogs act on cancer cell mitochondria and cause mitochondrial dysfunction directly.

### PST Analog-Induced Apoptosis is Dependent on Functional Complex II and III of the Mitochondrial Electron Transport Chain

**Figure 7:**
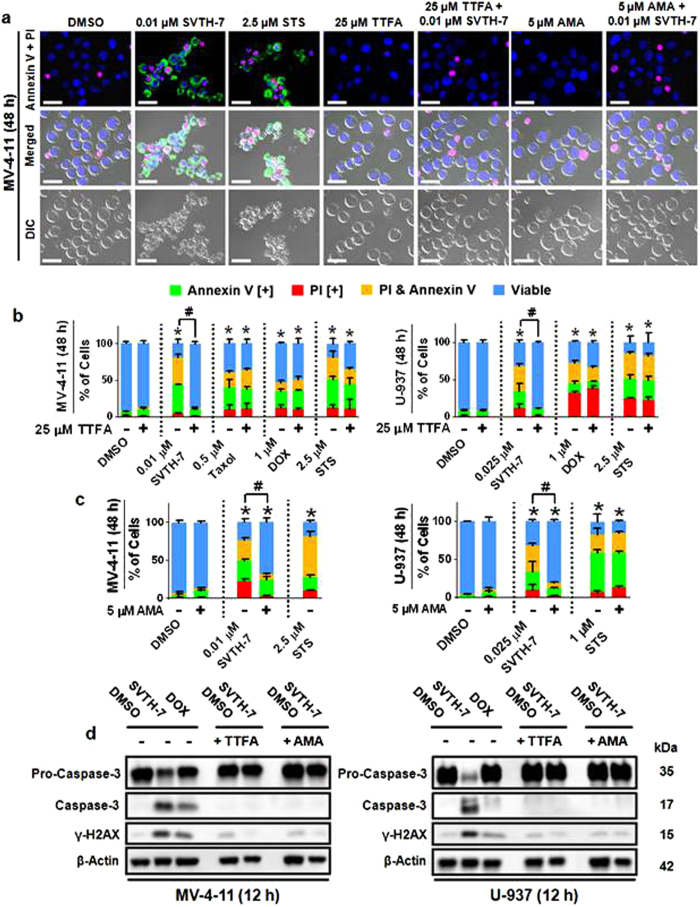
PST Analog-Induced Apoptosis is Dependent on Functional Complex II and III of the Mitochondrial Electron Transport Chain. MV-4-11, and U-937 cancer cells were pre-treated with TTFA and AMA for 1 hour and then treated with PST analog, staurosporine (STS), Doxorubicin (DOX), and Taxol for 48 hours. Annexin V binding (green) and PI staining (PI) (red) was observed with **(a)** microscopy and **(b,c)** quantified with image based cytometry. Scale bar = 25 μm. *p < 0.01 vs. DMSO control (comparison of viable cells only); ^#^*p* < 0.001 vs. respective groups without TTFA or AMA (comparison of viable cells only). **(d)** Western blot analysis of AMA and TTFA pre-treated cells treated with 1 μM DOX, and 0.01 μM SVTH-7 with MV-4-11 cells and 0.025 μM SVTH-7 with U-937 cells. Images are representative of 3 independent experiments.

As we have shown PST analogs to act on cancer cell mitochondria and affect their functioning, we investigated the role of mitochondrial ETC complexes in PST analog-induced apoptosis using the complex II inhibitor Thenoyltrifluoroacetone (TTFA) and the complex III inhibitor AMA^43^,^44^. Interestingly, TTFA was able to rescue these cells from SVTH-7 insult, making the percentage of dead cells statistically similar to those of the DMSO control following 48 hours of treatment, preventing Casp-3 activation and reducing the levels of γ-H2AX (Fig. 7a,b,d). Cell salvation by TTFA was specific to SVTH-7 as this inhibitor had no significant effect on Taxol, DOX and STS treatment. A slightly less dramatic rescue was observed with AMA (Fig. 7a,c,d). TTFA and AMA were also able to prevent SVTH-7-induced Casp-9 activation (Supplemental Fig. 5a).

**Figure 8:**
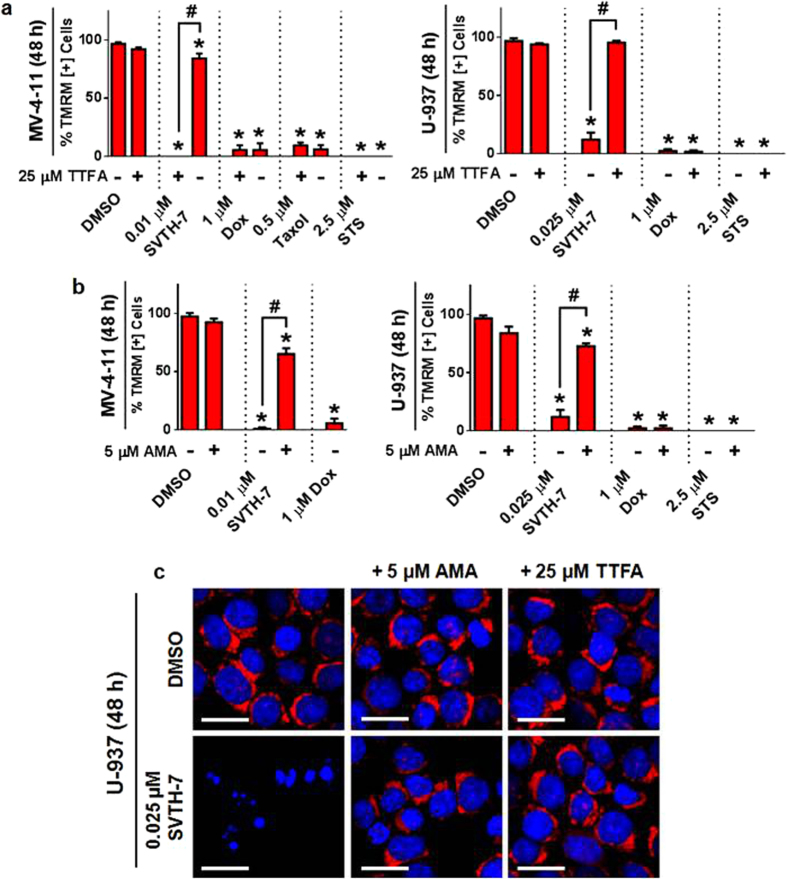
PST Analog-Induced MMP Dissipation is Dependent on Functional Complex II and III of the Mitochondrial Electron Transport Chain. MV-4-11 and U-937 cancer cells were pre-treated with (**a**) TTFA or (**b**) AMA for 1 hour and then treated with PST analogs, staurosporine (STS), Doxorubicin (DOX), and Taxol for 48 hours. TMRM fluorescence was quantified with image-based cytometry. *p < 0.01 vs. DMSO control; ^#^*p* < 0.001 vs. respective groups without TTFA or AMA. All values are expressed as mean ± SD from at least 3 independent experiments. (**c**) Representative fluorescent micrographs of 3 independent experiments of U-937 lymphoma cells stained with TMRM (red) and Hoechst (blue). Scale bar = 25 μm.

In addition, TTFA was able to protect cancer cell mitochondria from SVTH-7-induced dissipation of MMP and bring the percentage of TMRM positive cells to levels that are similar to values observed in the DMSO control treated group which can be seen at both 12 (Supplemental Fig. 6) and 48 hours (Fig. 8a and c). A similar but less dramatic rescue of MMP was observed with AMA (Supplemental Fig. 6 and Fig. 8b,c). Prevention of mitochondrial membrane permeabilization by TTFA was specific to SVTH-7 treatment as no significant changes in the percentage of TMRM positive cells was observed with Taxol, DOX, and STS treatment in conjunction with this inhibitor. Interestingly, inhibition of complex I with the inhibitor Rotenone (ROT)^44^ and uncoupling the ETC from ATP production with FCCP had no significant effect of SVTH-7 activity (Supplemental Figs 7 and 8). The functionality of the aforementioned ETC modulators on mitochondrial function was validated by an oxygen consumption assay as seen in Supplemental Fig. 9. Therefore, these observations imply that functional complex II, and, to a lesser extent, complex III are required for SVTH-7 to exert its pro-apoptotic effects in cancer cells.

### PST Analogs Selectively Induce Apoptosis in 3D Spheroid Models of Cancer

**Figure 9:**
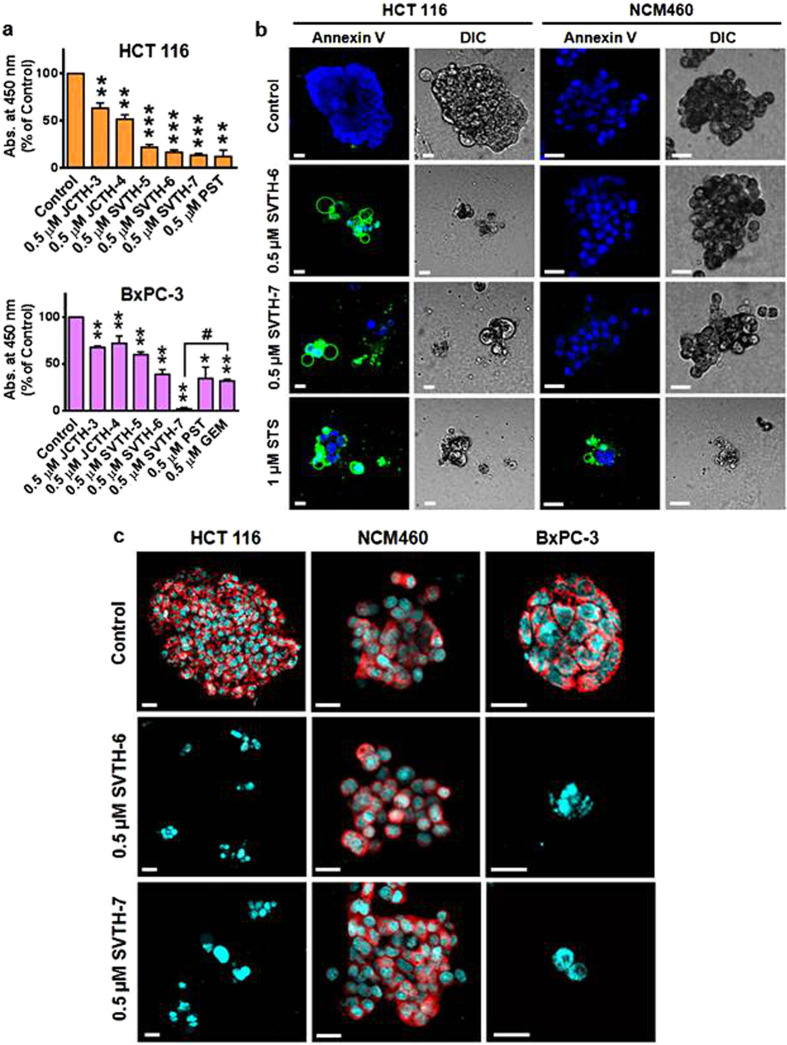
PST Analogs Selectively Induce Apoptosis in 3D Spheroid Models of Cancer. Cells were cultured on basement membrane extract (BMX) to form 3D spheroids, grown for 48 hours, and treated for 72 hours. **(a)** The WST-1 reagent was used to quantify viability. Absorbance was read at 450 nm and expressed as a percentage of control. Values are expressed as mean ± SD from triplicates of at least three independent experiments. *p < 0.05; **p < 0.005; ***p < 0.0005 vs. DMSO control. ^#^*p* < 0.001 vs. 0.5 μM GEM. **(b)** Confocal microscopy was used to monitor Annexin V binding (green) and **(c)** TMRM fluorescence (red). Cells were counterstained with NucRed Live 647 ReadyProbes^®^ Reagent to visualize nuclei (blue in B, cyan in C). Scale bar = 20 μm. Micrographs are representative of 3 independent experiments.

The three-dimensional architecture of tumors has been shown to dictate the responsiveness of cancer cells to chemotherapeutics^45^. To evaluate the efficacy of PST analogs in a more architecturally accurate context, cells were grown in three-dimensional spheroid culture on basement membrane extract (BMX) coated surfaces for 48 hours, which provides a scaffold for cells to form three-dimensional structures^46^, and treated with PST analogs for 72 hours. SVTH-7, -6, -5 and natural PST were the most effective on the HCT 116 colorectal cancer and BxPC-3 pancreatic cancer spheroids as determined by the WST-1 viability assay (Fig. 9a). Interestingly, SVTH-6 and natural PST had comparable anti-cancer activity compared to GEM, the current standard chemotherapeutic for pancreatic cancer^29^, in BxPC-3 spheroids while SVTH-7 had significantly superior activity compared to GEM.

Annexin V binding was monitored in HCT 116 colorectal cancer spheroids (Fig. 9b). Similarly to the STS positive control for apoptosis, HCT 116 cells treated with SVTH-6 and -7 were positive for Annexin V binding, indicated by the green fluorescence. This was accompanied by nuclear condensation and cell shrinkage, as depicted in fluorescence and corresponding DIC micrographs respectively, which are all indicative of apoptosis. Minimal Annexin V binding was present in the DMSO solvent control treated group. In the DIC micrograph of the solvent control, spheroids were dramatically larger and cells exhibited large, round, healthy cellular morphology. NCM460 normal colon mucosa spheroid cells were dramatically less sensitive to SVTH-6 and -7. Unlike the STS positive control, minimal Annexin V binding was observed in both the solvent control and SVTH-6 and -7 treated cells, which exhibited healthy nuclear and cell morphology.

MMP collapse was monitored as another marker of apoptosis (Fig. 9c)^47^. SVTH-6 and -7 were able to dissipate MMP in HCT 116 and BxPC-3 cancer cells in spheroid culture as indicated by the dissipation of red TMRM fluorescence. However, no such dissipation was evident in the NCM460 normal colon mucosa spheroid cells. Together, these results indicate that PST analogs SVTH-6 and -7 are both effective and selective against cancer cells grown in three-dimensional spheroid culture.

### PST Analogs Decrease Growth of Tumors in Xenograft Mouse Models

**Figure 10:**
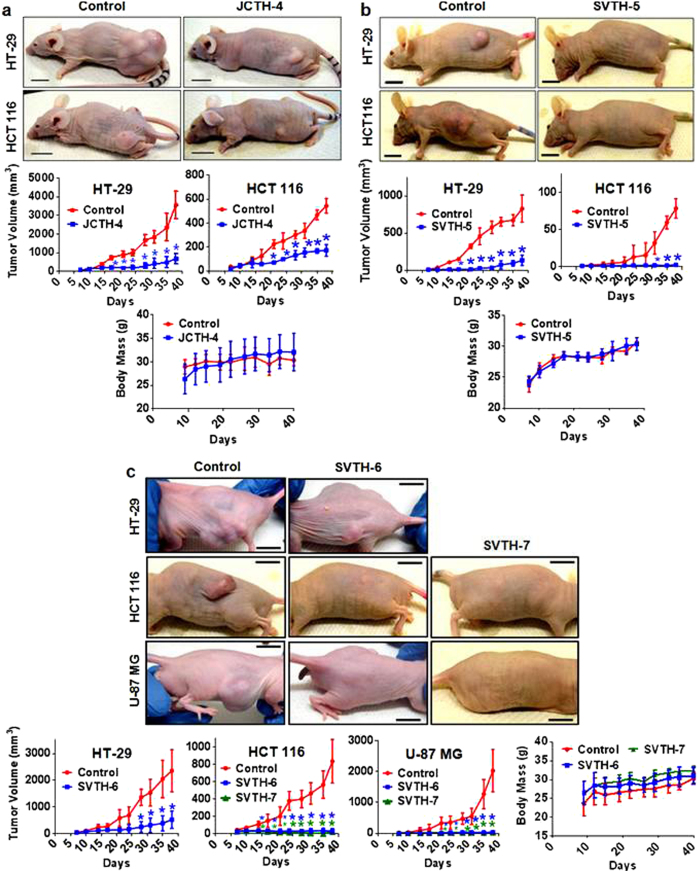
PST Analogs Decrease Growth of Tumors in Xenograft Mouse Models. Cancer cells were injected subcutaneously into the flanks of nude mice to establish tumors (day 0). After palpable tumors were detected (approximately 1 week), mice were treated via intratumoral injection with DMSO vehicle control or 3 mg/kg of **(a)** JCTH-4, **(b)** SVTH-5, **(c)** SVTH-6, and SVTH-7 3x/week for approximately 5 weeks. Scale bar for representative tumor sizes at time of sacrifice = 1 cm. Values for tumor volumes and body weights are expressed as mean ± SD (n = 4–6). *p < 0.05 vs. control. No significant difference in body masses between control and PST analog treated mice was observed.

To evaluate the anti-cancer activity of PST analogs *in vivo*, cancer cells were subcutaneously injected into the flanks of immunocompromised mice. After palpable tumors were established approximately 1 week after injections, mice were treated with 3 mg/kg of PST analogs intratumorally 3 times a week for approximately 5 weeks. JCTH-4 and SVTH-5 were able to reduce the growth of both HCT 116 and HT-29 colorectal tumor xenografts with SVTH-5 showing greater efficacy (Fig. 10a and b). SVTH-6 was also effective in reducing growth of HT-29 tumors (Fig. 10c). Furthermore, SVTH-6 and -7 were very effective in reducing the growth of HCT 116 colorectal cancer and U-87 MG glioblastoma tumor xenongrafts as tumor volumes were drastically smaller than the DMSO solvent control treated tumors (Fig. 10c). Mice treated with JCTH-4, SVTH-5, -6, and -7 all increased in mass throughout the studies and did not significantly differ from the masses of mice treated with DMSO solvent control (Fig. 10a–c). These findings demonstrate that PST analogs are able to decrease the growth of tumors *in vivo* and are well tolerated by mice at their effective doses.

## Discussion

Natural PST has been shown to be a promising anti-cancer agent in our previous work^23^. In this structure-activity relationship analysis, we have discovered that the anti-cancer activity of these analogs of PST is highly dependent on the C-7 hydroxyl group and the functional substitutions on C-1. Three novel synthetic analogs, SVTH-5, -6, and -7, with the full anti-cancer pharmacophore of PST, including the C-7 hydroxyl group (Fig. 1), were evaluated. Accordingly, SVTH-6 and -5 were more potent against cancer cells compared to their related compounds JCTH-4 and -3, respectively, which lack this functional group. Furthermore, the functional group at C-1 dramatically dictates analog potency. For example, JCTH-1 and JCTH-2 are only different from JCTH-4 in their C-1 functional groups and are nearly devoid of any anti-cancer activity (Figs 2 and 3a). Likewise, SVTH-7 differs only in the group at C-1, when compared to SVTH-6 and -5, and has much greater efficacy against most cancer cell lines tested (Figs 2 and 3a). These findings suggest the C-7 hydroxyl and, more heavily, the C-1 functional groups may play a particular role in interaction with the cellular target(s) of interest. The high anti-cancer activity of SVTH-7 may imply an interaction between the C-1 functional group and a specific hydrophobic pocket, as this analog possesses a bulky benzene ring at this position.

Very importantly, PST analogs demonstrated potent anti-cancer activity at low therapeutic doses with minimal effect in normal cells (Figs 2 and 3). In particular, SVTH-7, -6, and -5 demonstrated greater efficacy than the common chemotherapeutics Taxol, DOX, GEM, and Cisplatin in a multitude of cancer cell types including leukemia, triple negative breast cancer, pancreatic cancer, glioblastoma, and non-small cell lung cancer (Figs 2 and 3a). Additionally, these analogs were both effective and selective in inducing apoptosis in cancer cells grown in three-dimensional culture (Fig. 9), demonstrating their ability to penetrate tumor architecture and induce cell death in cells supported by extracellular matrix. More importantly, these analogs were able to reduce growth of tumors *in vivo* without any apparent toxicity to mice as there was no reduction in body mass and decrease in normal activity (Fig. 10). These compounds appear to show anti-cancer efficacy indicating that they are stable in physiological systems. Thus, these novel analogs show greater efficacy and extreme selectively in killing cancer cells than a number of standard chemotherapeutics and could provide safe and more efficacious anti-cancer treatment.

These analogs do not appear to affect tubulin dynamics (Supplemental Fig. 10), as with the chemotherapeutics Taxol and Colchicine, which would otherwise produce detrimental effects in normal fast dividing cells in the body^48^. Our findings indicate that this cancer selectivity may be attributed to the ability of these compounds to specifically target cancer cell mitochondria (Figs 4, 5, 6). The first cellular events observed with PST analog treatment were the formation of ROS and MMP dissipation, which was most evident with SVTH-7 as it was shown to show cause initial MMP dissipation as early as 1 to 3 hours in leukemia cells (Supplemental Fig. 3d and e, Figs 4b and 6a). Complimenting these findings, Casp-9 activation is first observed with mild activation of Casp-3 at 6 hours with no apparent DNA damage. At 12 hours, activation of both of these proteases is more pronounced and accompanied with DNA damage in MV-4-11 leukemia cells (Fig. 4a). This chronology of cellular events indicates that these compounds do not target DNA or cause DNA damage directly, but cause DNA damage as consequence of apoptotic induction. These results suggest that PST analogs act on cancer cell mitochondria to permeabilize these organelles and induce apoptosis. Supporting this rationale, ++Bcl-2 Jurkat cells were much less sensitive to PST analog-induced apoptosis and MMP dissipation compared to their counterparts without the over-expression of the anti-apoptotic protein (Fig. 5c–e). Moreover, PST analogs were able to decrease mitochondrial function as shown with a decrease in oxygen consumption within the first couple hours of treatment on E6-1 and U-937 cells (Fig. 6b). Furthermore, SVTH-6 and -7 were able to cause the phosphorylation of AMPK, a marker for cellular energy homeostasis at 6 hours and a total reduction in the amount of ATP at 12 hours to compliment the decrease in oxygen consumption rate, ultimately indicating mitochondrial dysfunction (Fig. 6c and d). Lastly, these analogs were able to act directly on mitochondria of MV-4-11 cells to cause release of the apoptogenic factor Cyto c (Fig. 6e).

Cancer cells have been shown to fortify their mitochondria with an abundance of anti-apoptotic proteins, including anti-apoptotic members of the Bcl-2 family of proteins, while downregulating pro-apoptotic proteins^13^. Moreover, heavy reliance on glycolysis and having relatively inactive mitochondria limits the generation of ROS by the ETC, further decreasing the likelihood of oxidative stress-induced apoptosis. Inhibiting these anti-apoptotic proteins, mimicking pro-apoptotic proteins, or targeting ETC complexes could be potential mechanisms employed by PST analogs.

Mounting evidence suggests targeting complexes of the ETC to be an effective strategy for targeting cancer cells^15^,^49^. ETC complex manipulation has been shown to increase ROS, which can promote apoptosis selectively as cancer cells have demonstrated to be more sensitive to oxidative stress^50^,^51^. Interestingly, inhibiting ETC complexes II and III abolishes the pro-apoptotic effects of the PST analog SVTH-7 on cancer cells and their mitochondria, with a slightly more pronounced effect with complex II inhibition (Figs 7 and 8). Interestingly, in the presence of several antioxidants, the cleavage of Casp-3 still occurred with the treatment of these analogs, indicating the dependence of the activity of these analogs to require inhibition of complex II activity, but not by the generation of ROS (Supplementary Fig. 11). It may be possible for this PST analog to directly target these complexes, an interacting partner of these complexes, exploit an unidentified feature of the metabolic state created by these functional complexes, or affect pathways downstream of these complexes. One such interacting partner of Complex II is TRAP1. This protein has been shown to have an inhibitory effect on complex II, acting as an anti-oxidant and producing anti-apoptotic effects in tumor cells^52^. Furthermore, succinate dehydrogenase (SDH) or complex II was found to be a mediator of apoptosis, producing ROS for cell death upon intracellular acidification^53^. However, further investigation is required to clarify the role of complex II and III in SVTH-7-mediated apoptosis.

This study comprehensively compares the activity of a number of PST analogs and has shown SVTH-7, followed by SVTH-6 and SVTH-5, to be the most effective against a battery of cancer cell lines, surpassing the anti-cancer activity of natural PST, and most importantly, several standard chemotherapeutics. These analogs were shown to target cancer cell mitochondria and be selective towards cancer cells in cell and animal models. The requirement of functional complex II and III for SVTH-7 to exert its pro-apoptotic effects in cancer cells points to a potential mitochondrial vulnerability in cancer cells that can be further characterized and exploited to strategically devise new treatment regimes. Therefore, this work provides a scaffold for characterizing distinct mitochondrial and metabolic characteristics in cancer cells that may be used to design novel therapeutic strategies and highlights several PST analogs with high therapeutic potential.

## Materials and Methods

### Cell Culture

The E6-1 Jurkat (acute T-cell leukemia) and BCL2 Jurkat (with over-expressed anti-apoptotic protein Bcl-2) cell lines (American Type Culture Collection, Cat. No. TIB-152 & CRL 2899, Manassas, VA, USA), were cultured with RPMI-1640 medium (Sigma-Aldrich Canada, Mississauga, ON, Canada) supplemented with 10% (v/v) fetal bovine serum (FBS) standard (Thermo Scientific, Waltham, MA, USA) and 10 mg/mL gentamicin (Gibco BRL, VWR, Mississauga, ON, Canada). The Jurkat that are dominant negative for the Fas-Associated Death Domain (FADD) protein (DN FADD Jurkat) (American Type Culture Collection, Cat. No. CRL-2572, Manassas, VA, USA) were also cultured as described above. DN FADD Jurkat cell line is a FADD mutant of the wild-type Jurkat cell line A3 (ATCC-CRL-2570) and is resistant to Fas-induced cell death^54^.

The MV-4-11 Chronic myelomonocitic leukemia cell line (ATCC, Cat. No. CRL-9591, Manassas, VA, USA). was cultured with Iscove’s Modified Dulbecco’s Medium (ATCC, Cat. No. 30-2005, Manassas, VA, USA) supplemented with 10% (v/v) FBS standard (Thermo Scientific, Waltham, MA, USA) and 10 mg/mL gentamicin (Gibco BRL, VWR, Mississauga, ON, Canada).

The U-937 histiocytic lymphoma cell line (ATCC, Cat. No. CRL-1593.2, Manassas, VA, USA). was cultured with Iscove’s Modified Dulbecco’s Medium (ATCC, Cat. No. 30-2005, Manassas, VA, USA) supplemented with 10% (v/v) FBS standard (Thermo Scientific, Waltham, MA, USA) and 10 mg/mL gentamicin (Gibco BRL, VWR, Mississauga, ON, Canada).

The MDA-MB-231 and MDA-MB-468 triple negative breast adenocarcinoma cell lines (ATCC, Cat. No. HTB-26 & HTB-132, Manassas, VA, USA) were cultured with Dulbecco’s Modified Eagles Medium HAM F12 (Sigma-Aldrich, Mississauga, ON, Canada) supplemented with 10% (v/v) FBS standard (Thermo Scientific, Waltham, MA, USA) and 10 mg/mL gentamicin (Gibco BRL, VWR, Mississauga, ON, Canada).

The SUM149 inflammatory breast cancer cell line (a generous gift from Dr. Stephen Ethier, Wayne State University, Detroit, MI, USA) was cultured in Dulbecco’s Modified Eagles Medium HAM F12 (Sigma-Aldrich, Mississauga, ON, Canada) supplemented with 5% (v/v) FBS standard (Thermo Scientific, Waltham, MA, USA), 10 mg/mL gentamicin (Gibco BRL, VWR, Mississauga, ON, Canada), 5 μg/ml insulin (Sigma-Aldrich, Mississauga, ON, Canada), and 1 μg/ml hydrocortisone (Sigma-Aldrich, Mississauga, ON, Canada).

The human colorectal cancer cell lines HT-29 and HCT 116 (ATCC, Cat. No. CCL-218 & CCL-247, Manassas, VA, USA) were cultured with McCoy’s Medium 5a (Gibco BRL, VWR, Mississauga, ON, Canada) supplemented with 2 mM L-glutamine, 10% (v/v), FBS (Thermo Scientific, Waltham, MA, USA) and 10 mg/ml gentamicin (Gibco, BRL, VWR, Mississauga, ON, Canada).

The BxPC-3 (ATCC, Cat. No. CRL-1687, Manassas, VA, USA) pancreatic adenocarcinoma cell line was grown in RPMI-1640 medium (Sigma-Aldrich Canada, Mississauga, ON, Canada) supplemented with 10% (v/v) FBS standard (Thermo Scientific, Waltham, MA, USA) and 10 mg/mL gentamicin (Gibco BRL, VWR, Mississauga, ON, Canada).

The PANC-1 (ATCC, Cat. No. CRL-1469, Manassas, VA, USA), epithelioid carcinoma cell line of the pancreas was grown in Dulbecco’s Modified Eagle’s Medium (Sigma-Aldrich Canada, Mississauga, ON, Canada) supplemented with 10% (v/v) FBS standard (Thermo Scientific, Waltham, MA, USA) and 10 mg/mL gentamicin (Gibco BRL, VWR, Mississauga, ON, Canada).

The osteosarcoma cell lines, U-2 OS and Saos-2 (ATCC, Cat. No. HTB-96 & HTB-85, Manassas, VA, USA), were grown in McCoy’s 5 A Medium Modified (Sigma-Aldrich Canada, Mississauga, ON, Canada). The U-2 OS medium was supplemented with 10% (v/v) FBS standard (Thermo Scientific, Waltham, MA, USA) and 10 mg/mL gentamicin (Gibco BRL, VWR, Mississauga, ON, Canada). The Saos-2 medium was supplemented with 15% (v/v) FBS standard (Thermo Scientific, Waltham, MA, USA) and 10 mg/mL gentamicin (Gibco BRL, VWR, Mississauga, ON, Canada).

The U-87 MG glioblastoma cell line (ATCC, Cat. No. HTB-14, Manassas, VA, USA) was grown with Eagle’s Minimum Essential Medium (Sigma-Aldrich Canada, Mississauga, ON, Canada) supplemented with 10% FBS standard (Thermo Scientific, Waltham, MA, USA) and 10 mg/mL gentamicin (Gibco BRL, VWR, Mississauga, ON, Canada).

The NCI-H23 non-small cell lung cancer cell line (ATCC, Cat. No. CRL-5800, Manassas, VA, USA) was grown and cultured in RPMI-1640 medium (Sigma-Aldrich Canada, Mississauga, ON, Canada) supplemented with 10% (v/v) FBS standard (Thermo Scientific, Waltham, MA, USA) and 10 mg/mL gentamicin (Gibco BRL, VWR, Mississauga, ON, Canada).

The A549 non-small cell lung cancer cell line (ATCC, Cat. No. CRM-CCL-185, Manassas, VA, USA) was grown and cultured in F-12K medium (ATCC, Cat. No. 30-2004, Manassas, VA, USA) supplemented with 10% (v/v) FBS standard (Thermo Scientific, Waltham, MA, USA) and 10 mg/mL gentamicin (Gibco BRL, VWR, Mississauga, ON, Canada).

The OVCAR-3 ovarian adenocarcinoma cell line (ATCC, Cat. No. HTB-161, Manassas, VA, USA) was grown and cultured in RPMI-1640 medium (Sigma-Aldrich Canada, Mississauga, ON, Canada) supplemented with 0.01 mg/mL bovine insulin, 20% (v/v) FBS standard (Thermo Scientific, Waltham, MA, USA) and 10 mg/mL gentamicin (Gibco BRL, VWR, Mississauga, ON, Canada).

The MCF7 human breast adenocarcinoma cell line (ATCC, Cat. No. HTB-22, Manassas, VA, USA) was grown in RPMI-1640 medium (Sigma-Aldrich Canada, Mississauga, ON, Canada) supplemented with 10% FBS standard (Thermo Scientific, Waltham, MA) and 10 mg/mL gentamicin (Gibco BRL, VWR, Mississauga, ON, Canada).

The G-361 malignant melanoma cell line (ATCC, Cat. No. CRL-1424, Manassas, VA, USA), was grown in McCoy’s 5 A Medium Modified (Sigma-Aldrich Canada, Mississauga, ON, Canada) supplemented with 10% (v/v) FBS standard (Thermo Scientific, Waltham, MA, USA) and 10 mg/mL gentamicin (Gibco BRL, VWR, Mississauga, ON, Canada).

The DU 145 prostate carcinoma cell line (ATCC, Cat. No. HTB-81, Manassas, VA, USA) was grown with Eagle’s Minimum Essential Medium (Sigma-Aldrich Canada, Mississauga, ON, Canada) supplemented with 10% FBS standard (Thermo Scientific, Waltham, MA, USA) and 10 mg/mL gentamicin (Gibco BRL, VWR, Mississauga, ON, Canada).

The Hep G2 human hepatoma cell line (ATCC, Cat. No. HB-8065, Manassas, VA, USA) was grown with Eagle’s Minimum Essential Medium (Sigma-Aldrich Canada, Mississauga, ON, Canada) supplemented with 10% FBS standard (Thermo Scientific, Waltham, MA, USA) and 10 mg/mL gentamicin (Gibco BRL, VWR, Mississauga, ON, Canada).

The AG09309 normal human skin fibroblasts (Coriell Institute for Medical Research, Cat. No. AG09309, Camden, NJ, USA) was grown in Dulbecco’s Modified Eagle’s Medium, High Glucose (Thermo Scientific, Waltham, MA, USA) supplemented with 15% (v/v) FBS and 10 mg/mL gentamicin (Gibco BRL, VWR, Mississauga, ON, Canada).

The CCD-18Co normal colon fibroblasts (ATCC, Cat. No. CRL-1459, Manassas, VA, USA) was grown with Eagle’s Minimum Essential Medium (Sigma-Aldrich Canada, Mississauga, ON, Canada) supplemented with 10% FBS standard (Thermo Scientific, Waltham, MA, USA) and 10 mg/mL gentamicin (Gibco BRL, VWR, Mississauga, ON, Canada).

The normal-derived colon mucosa (NCM460) cell line (INCELL Corporation, LLC., San Antonio, TX, USA) was grown in INCELL’s M3Base^TM^ medium (INCELL Corporation, LLC., Cat. No. M300A500) supplemented with 10% (v/v) FBS and 10 mg/mL gentamicin (Gibco BRL, VWR, Mississauga, ON, Canada).

The HEK-293 human embryonic kidney cell line (ATCC, Cat. No. CRL-1573, Manassas, VA, USA) was grown with Eagle’s Minimum Essential Medium (Sigma-Aldrich Canada, Mississauga, ON, Canada) supplemented with 10% FBS standard (Thermo Scientific, Waltham, MA, USA) and 10 mg/mL gentamicin (Gibco BRL, VWR, Mississauga, ON, Canada).

All cells were grown in optimal growth conditions of 37 °C and 5% CO_2_. Furthermore, all cells were cultured and passaged for less than 6 months and no authentication of cell lines was performed by the author.

### Isolation and Culture of Peripheral Blood Mononuclear cells (PBMCs)

All methods involving human experiments were performed in accordance with the relevant guidelines and regulations. All experiments involving human subjects (healthy volunteers donating blood) were done with prior approval of Research Ethics Board of the University of Windsor (protocol # REB #04-147), with informed consent obtained from all subjects. Peripheral blood mononuclear cells (PBMCs) were collected and isolated from healthy volunteers. In brief, whole blood was collected in BD Vacutainer^®^CPT^TM^ Tubes with Sodium Heparin^N^ (Becton, Dickinson and Company, Cat. No. 362753, Franklin Lakes, NJ, USA) at room temperature. Tubes were immediately inverted 5 times and centrifuged for 30 minutes at room temperature at 1500–1800 × g. The layer of PBMCs under the plasma layer in each tube was collected, pooled together, resuspended in 50 mL of PBS, and centrifuged at room temperature at 300 × g for 15 minutes. The supernatant was methodically aspirated without disturbing the pellet and PBMCs were resuspended and cultured in RPMI-1640 medium (Sigma-Aldrich Canada, Mississauga, ON, Canada), supplemented with 10% (v/v) FBS standard (Thermo Scientific, Waltham, MA, USA) and 10 mg/mL gentamicin (Gibco BRL, VWR, Mississauga, ON, Canada) at 37 °C and at 5% CO_2_. PBMCs from healthy volunteers 1, 2, 3, and 4 (PBMCs V1, PBMCs V2, PBMCs V3, PBMCs V4) were taken from a healthy 28 year old female, a healthy 18 year old male, a healthy 48 year old male, and a healthy 31 year old female respectively. To induce proliferation of PBMCs, they were treated with various doses of concanavalin a (Con A) (eBioscience, Cat. No. 00-4978-03, San Diego, CA, USA).

### Chemicals and Cell Treatment

Cells were treated with PST, PST Analogs, Taxol (Sigma-Aldrich Canada, Cat. No. T7402, Mississauga, ON, Canada), Staurosporine (STS) (Sigma-Aldrich Canada, Cat. No. S4400, Mississauga, ON, Canada), Doxorubicin (DOX) (Sigma-Aldrich Canada, Cat. No. D1515, Mississauga, ON, Canada), Gemcitabine (GEM) (Sigma-Aldrich Canada, Cat. No. G6423, Mississauga, ON, Canada), piperlongumine (PL) (INDOFINE Chemical Company, Inc., Cat. No. P-004, Hillsborough, NJ, USA), the broad spectrum caspase inhibitor, Z-VAD-FMK (EMD Chemicals, Gibbstown, NJ, USA), Antimycin A (AMA) (Sigma-Aldrich Canada, Cat. No. A8674, Mississauga, ON, Canada), Thenoyltrifluoroacetone (TTFA) (Sigma-Aldrich Canada, Cat. No. T27006, Mississauga, ON, Canada), rotenone (ROT) (Sigma-Aldrich Canada, Cat. No. R8875, Mississauga, ON, Canada), carbonyl cyanide-4-(trifluoromethoxy)phenylhydrazone (FCCP) (Sigma-Aldrich Canada, Cat. No. C2920, Mississauga, ON, Canada), mitoTEMPO (Sigma-Aldrich Canada, Cat. No. SML0737, Mississauga, ON, Canada) and EM20-25 (Sigma-Aldrich Canada, Cat. No. SML0183, Mississauga, ON, Canada) dissolved in DMSO stock solutions. *N*-Acetyl-L-cysteine (NAC) (Sigma-Aldrich Canada, Cat. No. A7250) was dissolved in double distilled water. PST analogs were produced by synthesis from bromobenzene^24^,^55^.

### WST-1 Assay for Cell Viability

The WST-1 based colorimetric assay (Roche Applied Science, Indianapolis, IN, USA) was performed to quantify cell viability via as a function of cellular metabolism. Cells were seeded in 96-well clear bottom tissue culture plates and grown for 24 hours. Subsequently, cells were treated with the indicated concentrations of chemicals for the indicated time durations. WST-1 reagent was incubated for 4 hours at 37 °C with 5% CO_2_. In actively metabolizing cells, the WST-1 reagent is cleaved by cellular enzymes to produce formazan. The presence of formazan was quantified via absorbance readings at 450 nm on a Wallac Victor^3^ ^TM^ 1420 Multilabel Counter (PerkinElmer, Woodbridge, ON, Canada). Absorbance readings were expressed as percentages of the solvent treated control group. Inhibitory dose-response curves (log(inhibitor) vs. response – Variable slope (four parameters)) were calculated using GraphPad Prism 6.

### Cell Death Analysis: Annexin V Binding Assay and Propidium Iodide (PI) Staining

The Annexin V binding assay and propidium iodide staining was done in parallel to monitor the externalization of phosphatidylserine on the outer cellular surface, a marker of early apoptosis, and cell permeabilization, a marker of necrotic or late apoptotic cell death, respectively. Cells were washed with phosphate buffer saline (PBS) and suspended in Annexin V binding buffer (10 mM HEPES, 140 mM NaCl, 2.5 mM CaCl2, pH 7.4) with green fluorescent Annexin V AlexaFluor-488 (1:20) (Life Technologies Inc, Cat. No. A13201, Burlington, ON, Canada) and 0.01 mg/mL of red fluorescent PI (Life Technologies Inc, Cat. No. P3566, Burlington, ON, Canada) for 15 minutes at 37 °C protected from light. The percentage of early (green), late apoptotic cells (green and red), and necrotic cells (red) were quantified using image-based cytometry with a Tali^®^ Image-Based Cytometer (Life Technologies Inc, Cat. No. T10796, Burlington, ON, Canada). Cells from at least 18 random fields were analyzed using both the green (ex. 458 nm; em. 525/20 nm) and red (ex. 530 nm; em. 585 nm) channels. Fluorescent micrographs were taken at 400x magnification using LAS AF6000 software with a Leica DMI6000 fluorescent microscope (Wetzlar, Germany). Cells monitored with microscopy were counterstained with Hoechst 33342 (Molecular Probes, Eugene, OR, USA) to visualize nuclei using a final concentration of 10 μM during the 15 minute incubation.

### Quantitation of Reactive Oxygen Species (ROS)

The small molecule 2′, 7′-dicholorofluorescin diacetate (H_2_DCFDA) was used to montitor whole cell ROS generation. H_2_DCFDA enters the cell and is deacetylated by esterases and oxidized by ROS to the highly fluorescent 2′, 7′-dicholorofluorescein (DCF) (excitation 495 nm; emission 529 nm). Cells were pretreated with 20 μM H_2_DCFDA (Sigma-Aldrich Canada, Cat. No. D6883, Mississauga, ON, Canada) for 30 minutes at 37 °C protected from light at 5% CO_2_. Cells were treated for the indicated durations, centrifuged at 600 × g for 5 minutes and suspended in PBS. Percentage of DCF positive cells was quantified using the Tali^®^ Image-Based Cytometer (Life Technologies Inc, Cat. No. T10796, Burlington, ON, Canada) using 12 random fields per group with the green channel (excitation 458 nm; emission 525/20 nm).

### Tetramethylrhodamine Methyl Ester (TMRM) Detection of Mitochondrial Membrane Potential

Tetramethylrhodamine methyl ester (TMRM) (Gibco BRL, VWR, Mississauga, ON, Canada) was used for detecting mitochondrial membrane potential (MMP), an indicator of healthy intact mitochondria. Treated cells were incubated with 100 nM TMRM in growth medium for 45 minutes at 37 °C and 5% CO_2_ protected from light. The percentage of TMRM cells was quantified using image-based cytometry with a Tali^®^ Image-Based Cytometer (Life Technologies Inc, Cat. No. T10796, Burlington, ON, Canada). Cells from at least 18 random fields were analyzed using the red channel (ex. 530 nm; em. 585 nm). Fluorescent micrographs were taken at 400x magnification using LAS AF6000 software with a Leica DMI6000 fluorescent microscope (Wetzlar, Germany). Cells monitored with microscopy were counterstained with Hoechst 33342 (Molecular Probes, Eugene, OR, USA) to visualize nuclei using a final concentration of 10 μM during the 45 minute incubation.

### Mitochondrial Isolation

To isolate mitochondria, cells were washed once in cold PBS, re-suspended in hypotonic buffer (1 mM EDTA, 5 mM Tris–HCl, 210 mM mannitol, 70 mM sucrose, 10 μM Leu-pep and Pep-A, 100 μM PMSF) and subjected to manual homogenization with a glass tissue grinder. Homogenized cells were centrifuged at 600 × g for 5 minutes at 4 °C. The supernatant was centrifuged at 15000 × g for 15 minutes at 4 °C and the mitochondrial pellet was suspended in cold reaction buffer (2.5 mM malate, 10 mM succinate, 10 μM Leu-pep and Pep-A, 100 μM PMSF in PBS).

### Treatment of Isolated Mitochondria & Evaluation of Apoptogenic Factor Release

Isolated mitochondria were treated with drugs at the indicated concentrations and incubated for 2 hours in cold reaction buffer (2.5 mM malate, 10 mM succinate, 10 μM Leu-pep, 10 μM Pep-A, and 100 μM PMSF in PBS). Following treatment, mitochondria samples were vortexed and centrifuged at 15,000 × g for 15 minutes at 4 °C. Western Blot analysis was performed on the resulting supernatants and mitochondrial pellets suspended in cold reaction buffer to screen for release or retention of apoptogenic factors.

### Western Blot Analyses

Protein samples were subjected to SDS-PAGE, transferred onto a PVDF membrane, and blocked with 5% w/v milk or BSA in TBST (Tris-Buffered Saline Tween-20) solution for 1 hour. Membranes were incubated with primary antibodies overnight at 4 °C: anti-caspase-8 antibody (1:1000) raised in mouse (Cell Signalling, Cat. No. 9746 S, Danvers, MA, USA), anti-caspase-9 antibody (1:1000) raised in rabbit (Cell Signalling, Cat. No. 9502, Danvers, MA, USA), anti-caspase-3 antibody (1:2000) (Novus Biologicals, Cat. No. NB100-56709V2, Littleton, CO, USA), anti-β-actin antibody (1:1000) (Santa Cruz Biotechnology, Inc., Cat. No. sc-81178, Paso Robles, CA, USA), anti-p-Histone H2A.X (Ser 139) (γ-H2AX) antibody (Santa Cruz Biotechnology, Inc., Cat. No. sc-101696, Paso Robles, CA, USA), anti-Bcl-2 antibody (Santa Cruz Biotechnology, Inc., Cat. No. sc-7382, Paso Robles, CA, USA), anti-cytochrome c (Cyto c) antibody (1:1000) raised in mice (Abcam, Cat. No. ab13575, Cambridge, MA, USA), anti-succinate dehydrogenase subunit A antibody (1:1000) raised in mice (Santa Cruz Biotechnology, Inc., sc-59687, Paso Robles, CA, USA), anti-phospho-AMPKα (Thr172) (p-AMPKα; 1:1000) raised in rabbit (Cell Signalling, Ca. No. 40H9, Danvers, MA, USA), anti-AMPKα 1 (1:1000) raised in rabbit (Abcam Canada, Cat. No. ab32047, Toronto, ON, Canada). Membranes were quickly rinsed twice, washed once for 15 minutes, and then washed twice for 5 minutes in TBST. Membranes were incubated with horseradish peroxidase-conjugated secondary antibodies for 1 hour: goat anti-mouse antibody (1:2000) (Novus Biologicals, Cat. No. NBP2- 30347H, Littleton, CO, USA), goat anti-rabbit antibody (1:2000) (Novus Biologicals, Cat. No. NBP2-30348H, Littleton, CO, USA). Membranes were quickly rinsed twice and washed for three 5 minute washes with TBST. Chemiluminescence reagent (Thermo Fisher Scientific, Cat. No. 34095, Rockford, IL, USA) was used for band visualization. Densitometry analyses were performed using ImageJ software.

### Oxygen Consumption Quantitation

The MitoXpress^®^ Xtra - Oxygen Consumption Assay [HS Method] (Luxcel Biosciences Ltd., Cat. No. MX-200, Cork, Ireland). 1 000 000 cells/well were seeded in a 96-well black clear bottom tissue culture plate and incubated for an hour at 37 °C and 5% CO_2_. On a heat pack, 10 μL of MitoXpress^®^ reagent was added to each well excluding the blanks, cells were treated with test compounds, the plate was shaken with a plate shaker, and 2 drops of pre-warmed high sensitivity mineral oil was added to each well to seal off the air supply. Bottom read fluorescence measurements were taken at Ex. 380 nm and Em. 650, every 2 minutes for 2 hours at 37 °C using a SpectraMax Gemini XS multi-well plate reader (Molecular Devices, Sunnyvale, CA, USA). Increases in fluorescence are indicative of oxygen consumption. Oxygen consumption rates were determined by calculating the slope of the linear regions of the oxygen consumption curves using GraphPad Prism 6 software.

### ATP Quantitation

Cellular ATP was quantified with a luciferase-luciferin ATP determination assay (Life Technologies Inc, Cat No. A22066, Burlington, ON, Canada). Cells were harvested and lysed with 0.1% NP40 lysis buffer (0.1% (v/v) NP40, 20nMTris HCl, 100 mM NaCl, 5 mM EDTA). Lsyate was centrifuged at 600 × g for 5 minutes at 4 °C, pellets were discarded and 10 μL of lysate was loaded into the wells of a white clear bottom 96-well microplate. Reaction solution (1 mM DTT, 0.5 mM D-luciferin, 1.25 μg/mL fire flu luciferase in 1X kit reaction buffer) was added to a total volume of 100 μL/well and incubated at 28 °C for 15 minutes protected from light. Luminescence was measured with a SpectraMax M5e (Molecular Devices, Sunnyvale, CA, USA) at 560 nm and the amount of ATP was determined with a standard curve made from known concentrations of ATP. Amount of ATP was expressed as a number of moles of ATP over μg of protein.

### Three-Dimensional Spheroid Culture and Assays

To establish three dimensional spheroid cultures, tissue culture plates were coated with basement membrane extract (BMX). Prior to coating, 3-D Culture Matrix™ Basement Membrane Extract Reduced Growth Factor (phenol red free) (Trevigen, Inc., Cat No. 3445-005-01, Gaithersburg, MD, USA) was thawed at 4 °C overnight. As BMX forms a solid gel at room temperature, clear 96-well tissue culture plates, 35 mm glass bottom tissue culture dishes (MatTek Corporation, Cat No. P35G-0-14-C, Ashland, MA, USA) and pipette tips were chilled at -20 °C overnight prior to use. On ice and in sterile conditions, clear 96-well tissue culture plate wells or the 14 mm diameter microwells of chilled 35 mm glass bottom tissue culture dishes, were coated with 35 μL and 50 μL BMX respectively, with chilled pipette tips and incubated for 10 minutes at 37 °C. Approximately 2000–4000 and 5000 cells were seeded in clear 96-well plate wells and microwells of glass bottom dishes respectively and grown in medium with 2% (v/v) BMX for 48 hours with 5% CO_2_ at 37 °C. Following 48 hours of incubation, medium was replaced with fresh medium supplemented with 2% (v/v) BMX and spheroids were treated with test compounds for 72 hours. Spheroids grown in 96-well plates and glass bottom dishes were subjected to the WST-1 Assay and confocal fluorescence microscopy, respectively. Confocal micrographs were taken with an Olympus Fluoview FV1000 confocal microscope (Olympus Corporation, Shinjuku, Tokyo, Japan) using a UPLSAPO 20X, 0.75 numerical aperture dry objective (Olympus Corporation, Shinjuku, Tokyo, Japan). Cells were counterstained with NucRed Live 647 ReadyProbes^®^ Reagent (Life Technologies Inc, Cat. No. R37106, Burlington, ON, Canada) to visualize nuclei.

### *In Vivo* Xenograft Models

All experiments involving animals (mice) and animal protocols were approved by the University of Windsor Animal Care Committee (AUPP # 14–15) in accordance with the Canadian Animal Care committee. Immunocompromised CD-1 nu/nu male mice (Charles River Laboratories, Cat. No. 086, Sherbrooke, QC, Canada) were housed in laboratory conditions of a 12-hour light/dark cycle, in accordance with the animal care protocols outlined in the University of Windsor Animal Care Committee (AUPP # 14–15). Mice were injected with 2 × 10^6^ HT-29, HCT 116, or U-87 MG cancer cells suspended in 200 μL of PBS using a 23-gauge needle and 1 mL syringe subcutaneously in the hind flanks of the mice. After establishment of palpable tumors (approximately 1 week), mice were treated by intratumoral injection (4–6 mice per group) three times a week for five weeks of 3 mg/kg of PST analogs or DMSO dissolved in 200 μL of PBS. Tumors were measured with calipers and volumes were calculated using the ellipsoid formula п/6 × length × width × height. Changes in body mass were measured with a scale to assess if treatments were well tolerated.

### Statistical Analysis

All statistics were performed by GraphPad Prism 6 statistical software. A p-value below 0.05 was considered significant. For the experiments with single variable measurements, which include quantification of MMP, and whole cell ROS, a One-Way ANOVA (nonparametric) was conducted and each sample’s mean was compared to the mean of the negative control (DMSO vehcile) unless otherwise specified. For experiments that contained multi-variables (e.g. multiple group comparisions), such as the quantification of live and dead cells, Two-Way ANOVA (nonparametric) was used and each sample’s mean was compared to the mean of the negative control (DMSO vehcile) unless otherwise specified.

## Additional Information

**How to cite this article**: Ma, D. *et al*. Cancer Cell Mitochondria Targeting by Pancratistatin Analogs is Dependent on Functional Complex II and III. *Sci. Rep.* **7**, 42957; doi: 10.1038/srep42957 (2017).

**Publisher's note:** Springer Nature remains neutral with regard to jurisdictional claims in published maps and institutional affiliations.

## Supplementary Material

Supplementary Data

## Figures and Tables

**Table 1:** IC_50_ Values of PST, PST Analogs, and Standard Chemotherapeutics.

Cell Line	IC_50_ Values (μM)								
	JCTH-3	JCTH-4	SVTH-5	SVTH-6	SVTH-7	PST	TAXOL	GEM	DOX
**MV-4-11**	0.67 ± 0.09	0.066 ± 0.003	0.11 ± 0.005	0.010 ± 0.002	0.0020 ± 0.0004	0.060 ± 0.09	0.085 ± 0.03		
**E6-1**	0.34 ± 0.01	0.19 ± 0.01	0.019 ± 0.006	0.052 ± 0.006	0.023 ± 0.001	0.068 ± 0.002			
**U-937**			0.16 ± 0.06	0.079 ± 0.02	0.00976 ± 0.0026	0.38 ± 0.25			
**MDA-MB -231**			0.24 ± 0.03	0.27 ± 0.1	0.089 ± 0.02		0.64 ± 0.2		2.03 ± 0.7
**MDA-MB-468**	0.35 ± 0.1	0.14 ± 0.02	0.11 ± 0.03	0.070 ± 0.01	0.35 ± 0.07	0.15 ± 0.03	1.15 ± 0.2		1.12 ± 0.2
**SUM149**	0.72 ± 0.07	0.44 ± 0.09	0.11 ± 0.007	0.28 ± 0.07	0.12 ± 0.01	0.17 ± 0.04	0.12 ± 0.04		
**HCT 116**	0.22 ± 0.02	0.35 ± 0.03	0.081 ± 0.006	0.095 ± 0.004	0.033 ± 0.002	0.12 ± 0.001			
**BxPC-3**		0.26 ± 0.002	0.17 ± 0.01	0.11 ± 0.01	0.0068 ± 0.0005			0.12 ± 0.02	
**PANC-1**			0.44 ± 0.03	0.62 ± 0.07	0.95 ± 0.3			>10.0	
**SAOS-2**	0.34 ± 0.02	0.33 ± 0.04	0.12 ± 0.003	0.074 ± 0.001	0.057 ± 0.001				
**U-2 OS**	0.50 ± 0.1	0.23 ± 0.02	0.13 ± 0.006	0.070 ± 0.02					
**U-87 MG**		0.22 ± 0.05	0.15 ± 0.008	0.088 ± 0.003	0.0090 ± 0.002	0.20 ± 0.03	>10.0		
**NCI-H23**		1.4 ± 0.6	1.2 ± 0.3	0.39 ± 0.1	0.80 ± 0.6				
**A549**	1.1 ± 0.03	1.2 ± 0.3	0.61 ± 0.2	0.60 ± 0.4	0.23 ± 0.04	0.47 ± 0.2			
**OVCAR-3**	0.39 ± 0.03	0.34 ± 0.02	0.054 ± 0.005	0.11 ± 0.006					
**MCF7**	1.0 ± 0.06	1.2 ± 0.1	0.10 ± 0.01	0.74 ± 0.1	0.15 ± 0.01	0.12 ± 0.04	>10.0		
**G-361**	1.6 ± 0.3	0.20 ± 0.007	0.11 ± 0.03	0.055 ± 0.0007	0.093 ± 0.01				
**DU 145**			0.14 ± 0.02	0.075 ± 0.006					
**AG09309**	>2.5	>2.5	>2.5	>2.5	>2.5	>2.5			
**CCD -18Co**			>2.5	>2.5	0.37 ± 0.1	>2.5			

Values are given in units of μM expressed as mean ± SD of at least three independent experiments.

## References

[b1] Tait S. W. G. & Green D. R. Mitochondria and cell death: outer membrane permeabilization and beyond. Nat. Rev. Mol. Cell Biol. 11, 621–32 (2010). 20683470 10.1038/nrm2952

[b2] Fulda S. & Debatin K.-M. Extrinsic versus intrinsic apoptosis pathways in anticancer chemotherapy. Oncogene 25, 4798–811 (2006). 16892092 10.1038/sj.onc.1209608

[b3] Brown J. M. & Attardi L. D. The role of apoptosis in cancer development and treatment response. Nat. Rev. Cancer 5, 231–7 (2005). 15738985 10.1038/nrc1560

[b4] Baig S. . Potential of apoptotic pathway-targeted cancer therapeutic research: Where do we stand? Cell Death Dis. 7, e2058 (2016). 26775709 10.1038/cddis.2015.275 PMC4816162

[b5] Vander Heiden M. G., Cantley L. C. & Thompson C. B. Understanding the Warburg effect: the metabolic requirements of cell proliferation. Science 324, 1029–33 (2009). 19460998 10.1126/science.1160809 PMC2849637

[b6] DeBerardinis R. J., Lum J. J., Hatzivassiliou G. & Thompson C. B. The biology of cancer: metabolic reprogramming fuels cell growth and proliferation. Cell Metab. 7, 11–20 (2008). 18177721 10.1016/j.cmet.2007.10.002

[b7] Gogvadze V., Zhivotovsky B. & Orrenius S. The Warburg effect and mitochondrial stability in cancer cells. Mol. Aspects Med. 31, 60–74 (2010). 19995572 10.1016/j.mam.2009.12.004

[b8] Plas D. R. & Thompson C. B. Cell metabolism in the regulation of programmed cell death. Trends Endocrinol. Metab. 13, 75–8 (2002). 11854022 10.1016/s1043-2760(01)00528-8

[b9] Schulze A. & Harris A. L. How cancer metabolism is tuned for proliferation and vulnerable to disruption. Nature 491, 364–73 (2012). 23151579 10.1038/nature11706

[b10] Chen G., Wang F., Trachootham D. & Huang P. Preferential killing of cancer cells with mitochondrial dysfunction by natural compounds. Mitochondrion 10, 614–25 (2010). 20713185 10.1016/j.mito.2010.08.001 PMC3085019

[b11] Ralph S. J., Low P., Dong L., Lawen A. & Neuzil J. Mitocans: mitochondrial targeted anti-cancer drugs as improved therapies and related patent documents. Recent Pat. Anticancer. Drug Discov. 1, 327–46 (2006). 18221044 10.2174/157489206778776952

[b12] Rohlena J., Dong L.-F. & Neuzil J. Targeting the mitochondrial electron transport chain complexes for the induction of apoptosis and cancer treatment. Curr. Pharm. Biotechnol. 14, 377–89 (2013). 22201598 10.2174/1389201011314030011

[b13] Yip K. W. & Reed J. C. Bcl-2 family proteins and cancer. Oncogene 27, 6398–406 (2008). 18955968 10.1038/onc.2008.307

[b14] Yan B. . Mitochondrially targeted vitamin E succinate efficiently kills breast tumour-initiating cells in a complex II-dependent manner. BMC Cancer 15, 401 (2015). 25967547 10.1186/s12885-015-1394-7 PMC4494715

[b15] Rohlenova K. . Selective disruption of respiratory supercomplexes as a new strategy to suppress Her2-high breast cancer. Antioxid. Redox Signal, doi: 10.1089/ars.2016.6677 (2016). PMC5206771 27392540

[b16] Neuzil J., Dong L.-F., Rohlena J., Truksa J. & Ralph S. J. Classification of mitocans, anti-cancer drugs acting on mitochondria. Mitochondrion 13, 199–208 (2013). 22846431 10.1016/j.mito.2012.07.112

[b17] Kekre N., Griffin C., McNulty J. & Pandey S. Pancratistatin causes early activation of caspase-3 and the flipping of phosphatidyl serine followed by rapid apoptosis specifically in human lymphoma cells. Cancer Chemother. Pharmacol. 56, 29–38 (2005). 15726366 10.1007/s00280-004-0941-8

[b18] McLachlan A., Kekre N., McNulty J. & Pandey S. Pancratistatin: a natural anti-cancer compound that targets mitochondria specifically in cancer cells to induce apoptosis. Apoptosis 10, 619–30 (2005). 15909123 10.1007/s10495-005-1896-x

[b19] Siedlakowski P. . Synergy of Pancratistatin and Tamoxifen on breast cancer cells in inducing apoptosis by targeting mitochondria. Cancer Biol. Ther. 7, 376–84 (2008). 18075307 10.4161/cbt.7.3.5364

[b20] Chatterjee S. J., McNulty J. & Pandey S. Sensitization of human melanoma cells by tamoxifen to apoptosis induction by pancratistatin, a nongenotoxic natural compound. Melanoma Res, doi: 10.1097/CMR.0b013e328337abff (2010). 20300039

[b21] Griffin C., Hamm C., McNulty J. & Pandey S. Pancratistatin induces apoptosis in clinical leukemia samples with minimal effect on non-cancerous peripheral blood mononuclear cells. Cancer Cell Int. 10, 6 (2010). 20205924 10.1186/1475-2867-10-6 PMC2845577

[b22] Griffin C., McNulty J. & Pandey S. Pancratistatin induces apoptosis and autophagy in metastatic prostate cancer cells. Int. J. Oncol. 38, 1549–56 (2011). 21424119 10.3892/ijo.2011.977

[b23] Griffin C., Karnik A., McNulty J. & Pandey S. Pancratistatin selectively targets cancer cell mitochondria and reduces growth of human colon tumor xenografts. Mol. Cancer Ther. 10, 57–68 (2011). 21220492 10.1158/1535-7163.MCT-10-0735

[b24] Vshyvenko S., Scattolon J., Hudlicky T., Romero A. E. & Kornienko A. Synthesis of C-1 homologues of pancratistatin and their preliminary biological evaluation. Bioorg. Med. Chem. Lett. 21, 4750–2 (2011). 21757350 10.1016/j.bmcl.2011.06.068 PMC3205978

[b25] Ma D. . Selective cytotoxicity against human osteosarcoma cells by a novel synthetic C-1 analogue of 7-deoxypancratistatin is potentiated by curcumin. PLoS One 6, e28780 (2011). 22205968 10.1371/journal.pone.0028780 PMC3244407

[b26] Ma D. . A novel synthetic C-1 analogue of 7-deoxypancratistatin induces apoptosis in p53 positive and negative human colorectal cancer cells by targeting the mitochondria: enhancement of activity by tamoxifen. Invest. New Drugs 30, 1012–27 (2012). 21494837 10.1007/s10637-011-9668-7

[b27] Ma D., Collins J., Hudlicky T. & Pandey S. Enhancement of apoptotic and autophagic induction by a novel synthetic C-1 analogue of 7-deoxypancratistatin in human breast adenocarcinoma and neuroblastoma cells with tamoxifen. J. Vis. Exp, doi: 10.3791/3586 (2012). PMC3468192 22688195

[b28] Lips E. H. . Next generation sequencing of triple negative breast cancer to find predictors for chemotherapy response. Breast Cancer Res. 17, 134 (2015). 26433948 10.1186/s13058-015-0642-8 PMC4592753

[b29] Berlin J. & Benson A. B. Chemotherapy: Gemcitabine remains the standard of care for pancreatic cancer. Nat. Rev. Clin. Oncol. 7, 135–7 (2010). 20190797 10.1038/nrclinonc.2010.16

[b30] Fadok V. A., Bratton D. L., Frasch S. C., Warner M. L. & Henson P. M. The role of phosphatidylserine in recognition of apoptotic cells by phagocytes. Cell Death Differ. 5, 551–62 (1998). 10200509 10.1038/sj.cdd.4400404

[b31] Poon I. K. H., Hulett M. D. & Parish C. R. Molecular mechanisms of late apoptotic/necrotic cell clearance. Cell Death Differ. 17, 381–97 (2010). 20019744 10.1038/cdd.2009.195

[b32] Belmokhtar C. A., Hillion J. & Ségal-Bendirdjian E. Staurosporine induces apoptosis through both caspase-dependent and caspase-independent mechanisms. Oncogene 20, 3354–62 (2001). 11423986 10.1038/sj.onc.1204436

[b33] Shi Y. A structural view of mitochondria-mediated apoptosis. Nat. Struct. Biol. 8, 394–401 (2001). 11323712 10.1038/87548

[b34] Ow Y.-L. P., Green D. R., Hao Z. & Mak T. W. Cytochrome c: functions beyond respiration. Nat. Rev. Mol. Cell Biol. 9, 532–42 (2008). 18568041 10.1038/nrm2434

[b35] Fischer U., Jänicke R. U. & Schulze-Osthoff K. Many cuts to ruin: a comprehensive update of caspase substrates. Cell Death Differ. 10, 76–100 (2003). 12655297 10.1038/sj.cdd.4401160 PMC7091709

[b36] Kabeya Y. . LC3, a mammalian homologue of yeast Apg8p, is localized in autophagosome membranes after processing. EMBO J. 19, 5720–8 (2000). 11060023 10.1093/emboj/19.21.5720 PMC305793

[b37] Gamen S. . Doxorubicin treatment activates a Z-VAD-sensitive caspase, which causes deltapsim loss, caspase-9 activity, and apoptosis in Jurkat cells. Exp. Cell Res. 258, 223–35 (2000). 10912804 10.1006/excr.2000.4924

[b38] Milanesi E. . The mitochondrial effects of small organic ligands of BCL-2: sensitization of BCL-2-overexpressing cells to apoptosis by a pyrimidine-2,4,6-trione derivative. J. Biol. Chem. 281, 10066–72 (2006). 16481323 10.1074/jbc.M513708200

[b39] Lin M. T. & Beal M. F. Mitochondrial dysfunction and oxidative stress in neurodegenerative diseases. Nature 443, 787–95 (2006). 17051205 10.1038/nature05292

[b40] Raj L. . Selective killing of cancer cells by a small molecule targeting the stress response to ROS. Nature 475, 231–4 (2011). 21753854 10.1038/nature10167 PMC3316487

[b41] Cochemé H. M. & Murphy M. P. Complex I is the major site of mitochondrial superoxide production by paraquat. J. Biol. Chem. 283, 1786–98 (2008). 18039652 10.1074/jbc.M708597200

[b42] Brand M. D. & Nicholls D. G. Assessing mitochondrial dysfunction in cells. Biochem. J. 435, 297–312 (2011). 21726199 10.1042/BJ20110162 PMC3076726

[b43] Chen Y., McMillan-Ward E., Kong J., Israels S. J. & Gibson S. B. Mitochondrial electron-transport-chain inhibitors of complexes I and II induce autophagic cell death mediated by reactive oxygen species. J. Cell Sci. 120, 4155–66 (2007). 18032788 10.1242/jcs.011163

[b44] Spinazzi M., Casarin A., Pertegato V., Salviati L. & Angelini C. Assessment of mitochondrial respiratory chain enzymatic activities on tissues and cultured cells. Nat. Protoc. 7, 1235–46 (2012). 22653162 10.1038/nprot.2012.058

[b45] Imamura Y. . Comparison of 2D- and 3D-culture models as drug-testing platforms in breast cancer. Oncol. Rep. 33, 1837–43 (2015). 25634491 10.3892/or.2015.3767

[b46] Lee G. Y., Kenny P. A., Lee E. H. & Bissell M. J. Three-dimensional culture models of normal and malignant breast epithelial cells. Nat. Methods 4, 359–65 (2007). 17396127 10.1038/nmeth1015 PMC2933182

[b47] Kroemer G., Galluzzi L. & Brenner C. Mitochondrial membrane permeabilization in cell death. Physiol. Rev. 87, 99–163 (2007). 17237344 10.1152/physrev.00013.2006

[b48] Jordan M. A. & Wilson L. Microtubules as a target for anticancer drugs. Nat. Rev. Cancer 4, 253–265 (2004). 15057285 10.1038/nrc1317

[b49] Neuzil J., Wang X.-F., Dong L.-F., Low P. & Ralph S. J. Molecular mechanism of ‘mitocan’-induced apoptosis in cancer cells epitomizes the multiple roles of reactive oxygen species and Bcl-2 family proteins. FEBS Lett. 580, 5125–9 (2006). 16979626 10.1016/j.febslet.2006.05.072

[b50] Trachootham D. . Selective killing of oncogenically transformed cells through a ROS-mediated mechanism by β-phenylethyl isothiocyanate. Cancer Cell 10, 241–252 (2006). 16959615 10.1016/j.ccr.2006.08.009

[b51] Trachootham D., Alexandre J. & Huang P. Targeting cancer cells by ROS-mediated mechanisms: a radical therapeutic approach? Nat. Rev. Drug Discov. 8, 579–91 (2009). 19478820 10.1038/nrd2803

[b52] Guzzo G., Sciacovelli M., Bernardi P. & Rasola A. Inhibition of succinate dehydrogenase by the mitochondrial chaperone TRAP1 has anti-oxidant and anti-apoptotic effects on tumor cells. Oncotarget 5, 11897–908 (2014). 25564869 10.18632/oncotarget.2472 PMC4323003

[b53] Lemarie A., Huc L., Pazarentzos E., Mahul-Mellier A.-L. & Grimm S. Specific disintegration of complex II succinate:ubiquinone oxidoreductase links pH changes to oxidative stress for apoptosis induction. Cell Death Differ. 18, 338–49 (2011). 20706275 10.1038/cdd.2010.93 PMC3044456

[b54] Juo P., Kuo C. J., Yuan J. & Blenis J. Essential requirement for caspase-8/FLICE in the initiation of the Fas-induced apoptotic cascade. Curr. Biol. 8, 1001–8 (1998). 9740801 10.1016/s0960-9822(07)00420-4

[b55] Collins J. . Chemoenzymatic synthesis of Amaryllidaceae constituents and biological evaluation of their C-1 analogues. The next generation synthesis of 7-deoxypancratistatin and trans-dihydrolycoricidine. J. Org. Chem. 75, 3069–84 (2010). 20373760 10.1021/jo1003136 PMC2872072

