# Corrigendum: Cancer Cell Mitochondria Targeting by Pancratistatin Analogs is Dependent on Functional Complex II and III

**DOI:** 10.1038/srep46998

**Published:** 2018-07-04

**Authors:** Dennis Ma, Christopher Pignanelli, Daniel Tarade, Tyler Gilbert, Megan Noel, Fadi Mansour, Scott Adams, Alexander Dowhayko, Kyle Stokes, Sergey Vshyvenko, Jonathan Collins, Tomas Hudlicky, James McNulty, Siyaram Pandey

Scientific Reports 7; Article number: 4295710.1038/srep42957; published online: 02 21 2017; updated: 07 04 2018

Jonathan Collins was omitted from the author list in the original version of this Article. This has been corrected in the PDF and HTML versions of the Article, and in the accompanying Supplementary Data file.

The Author Contributions section now reads:

“Dennis Ma and Siyaram Pandey contributed to the conception and design of experiments, execution of experiments, analysis of data, and preparation of the manuscript. Daniel Tarade, Tyler Gilbert, Christopher Pignanelli, Fadi Mansour, Scott Adams, Kyle Stokes, Alexander Dowhayko, and Megan Noel contributed to the execution of experiments and preparation of the manuscript. Sergey Vshyvenko, Jonathan Collins and Tomas Hudlicky synthesized and provided PST analogs for this study and contributed to the preparation of the manuscript. James McNulty provided natural PST for this study and contributed to the preparation of the manuscript”.

