# Supplementary material for: Cancer Cell Mitochondria Targeting by Pancratistatin Analogs is Dependent on Functional Complex II and III
Source: Sci Rep. 2017 Feb 21;7:42957. doi: 10.1038/srep42957 (PMC5318952; doi:10.1038/srep42957)
Supplement: Supplementary Data [file srep42957-s1.doc]

**Cancer Cell Mitochondria Targeting by Pancratistatin Analogs is Dependent on Functional Complex II and III**

**Author list:**

Dennis Ma^1^, Christopher Pignanelli^1^, Daniel Tarade^1^, Tyler Gilbert^1^, Megan Noel^1^, Fadi Mansour^1^, Scott Adams^1^, Alexander Dowhayko^1^, Kyle Stokes^1^, Sergey Vshyvenko^2^, Tomas Hudlicky^2^, James McNulty^3^, and Siyaram Pandey^1^

^1*^Department of Chemistry and Biochemistry, University of Windsor,

401 Sunset Avenue, Windsor, Ontario N9B 3P4, Canada

Phone: +519-253-3000, ext. 3701

spandey@uwindsor.ca

^2^ Chemistry Department and Centre for Biotechnology, Brock University, 500 Glenridge Avenue, St. Catharines, Ontario L2S 3A1, Canada

thudlicky@brocku.ca

^3^ Department of Chemistry, McMaster University, 1280 Main Street West, Hamilton, Ontario L8S 4M1, Canada

jmcnult@mcmaster.ca

**Running Title:**

Synthetic Pancratistatin Analogs for Cancer Therapy

**Key Words:**

Cancer, mitochondria, apoptosis, cancer therapy, pancratistatin, synthetic analogs, drug discovery, xenograft models, spheroid culture, electron transport chain.


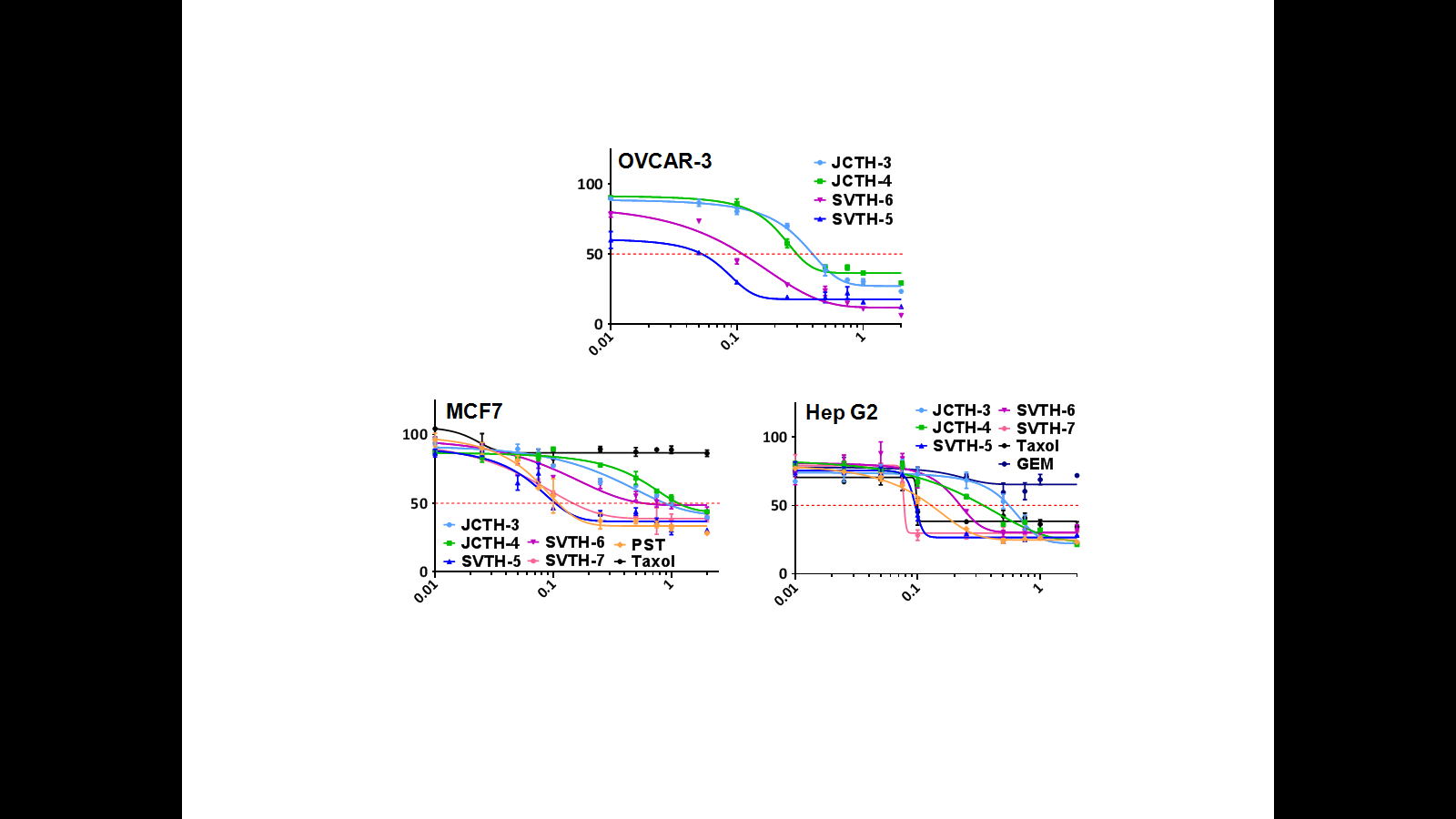


**Supplemental Figure 1a. PST Analogs & PST Decrease Viability of Cancer Cells in a Dose Dependent Manner.** The WST-1 colorimetric assay was performed on OVCAR-3 ovarian adenocarcinoma, MCF7 breast adenocarcinoma, and Hep G2 hepatoma cells treated with the indicated concentrations of compounds for 48 hours. The WST-1 reagent was added and the absorbance of the processed WST-1 reagent formazan, used to quantify cell viability, was read at 450 nm and expressed as a percent of solvent control (DMSO). Values are expressed as mean ± SD from quadruplicates of 3 independent experiments. X-axis = Concentration (μM); Y-axis = Absorbance at 450 (% of DMSO Control).


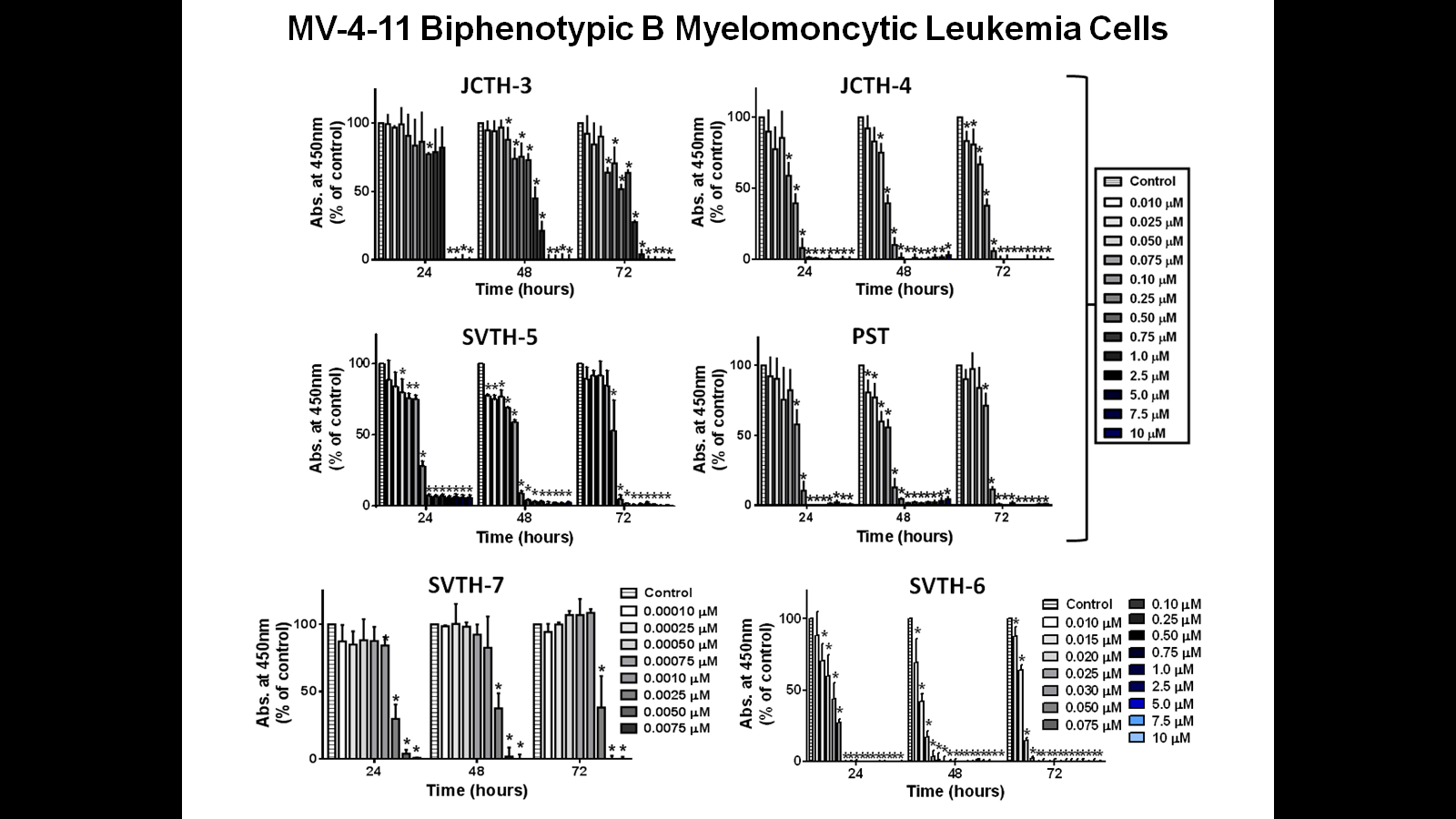


**Supplemental Figure 1b. PST Analogs & PST Decrease Viability of MV-4-11 Leukemia Cells in a Time and Dose Dependent Manner.** The WST-1 colorimetric assay was performed on MV-4-11 cells treated with the indicated concentrations of compounds for the indicated durations. The WST-1 reagent was added and the absorbance of the processed WST-1 reagent formazan, used to quantify cell viability, was read at 450 nm and expressed as a percent of solvent control (DMSO). Values are expressed as mean ± SD from quadruplicates of 3 independent experiments. **p*<0.05 vs. solvent control (DMSO).


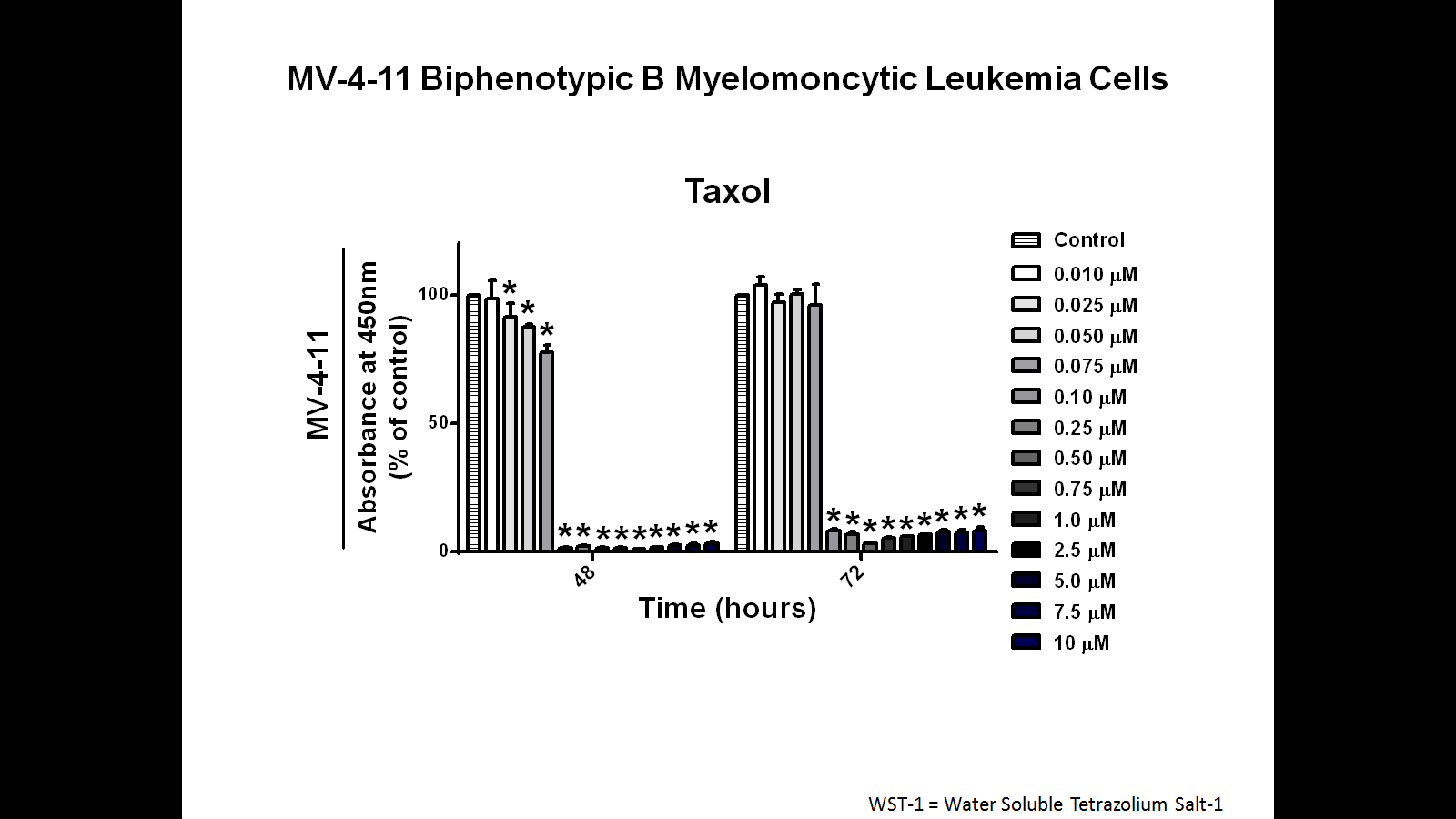


**Supplemental Figure 1c. Taxol Decreases Viability of MV-4-11 Leukemia Cells.** The WST-1 colorimetric assay was performed on MV-4-11 cells treated with the indicated concentrations of Taxol for the indicated durations. The WST-1 reagent was added and the absorbance of the processed WST-1 reagent formazan, used to quantify cell viability, was read at 450 nm and expressed as a percent of solvent control (DMSO). Values are expressed as mean ± SD from quadruplicates of 3 independent experiments. **p*<0.05 vs. solvent control (DMSO).


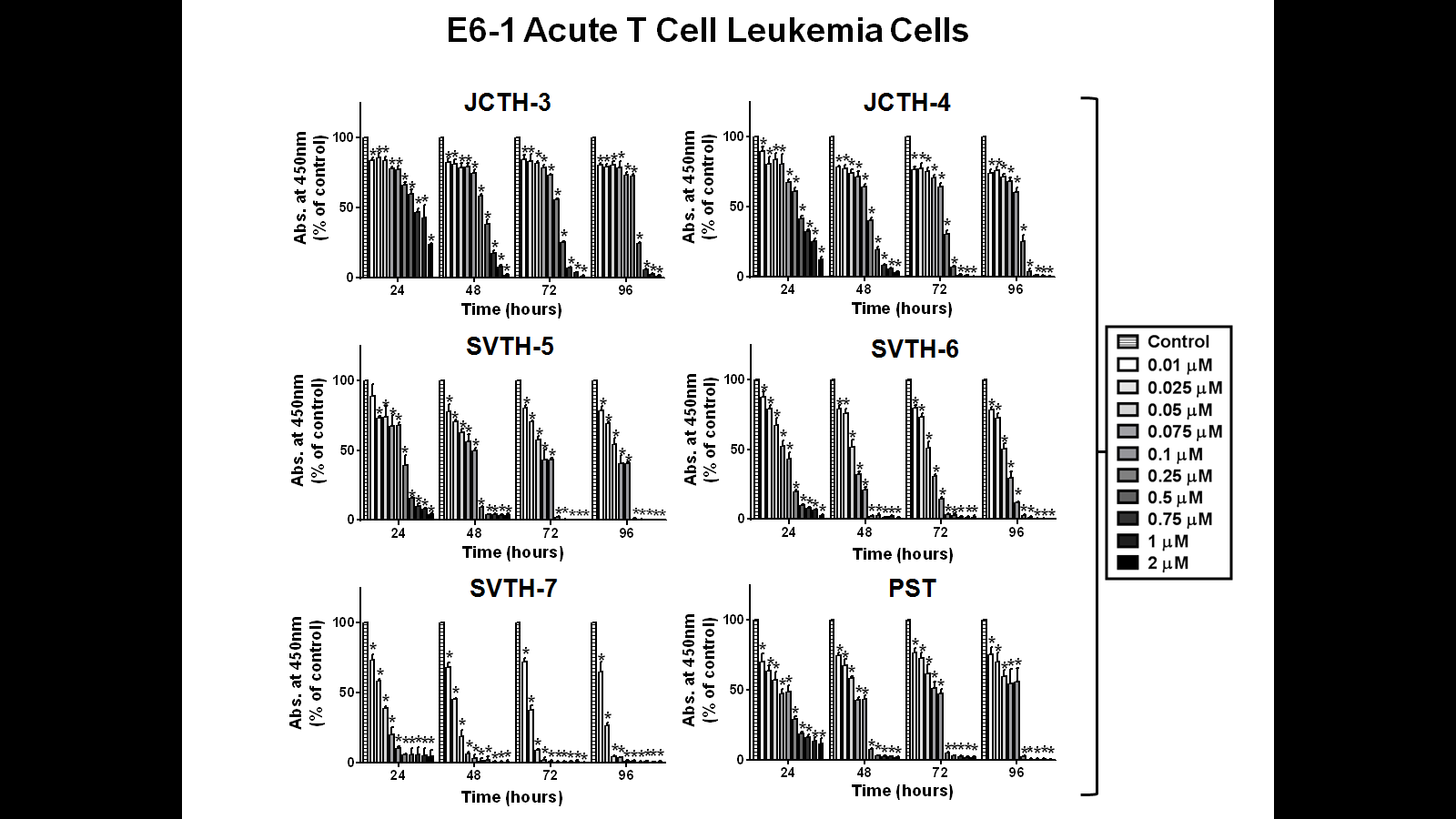


**Supplemental Figure 1d. PST Analogs & PST Decrease Viability of E6-1 Leukemia Cells in a Time and Dose Dependent Manner.** The WST-1 colorimetric assay was performed on E6-1 leukemai cells treated with the indicated concentrations of compounds for the indicated durations. The WST-1 reagent was added and the absorbance of the processed WST-1 reagent formazan, used to quantify cell viability, was read at 450 nm and expressed as a percent of solvent control (DMSO). Values are expressed as mean ± SD from quadruplicates of 3 independent experiments. **p*<0.05 vs. solvent control (DMSO).


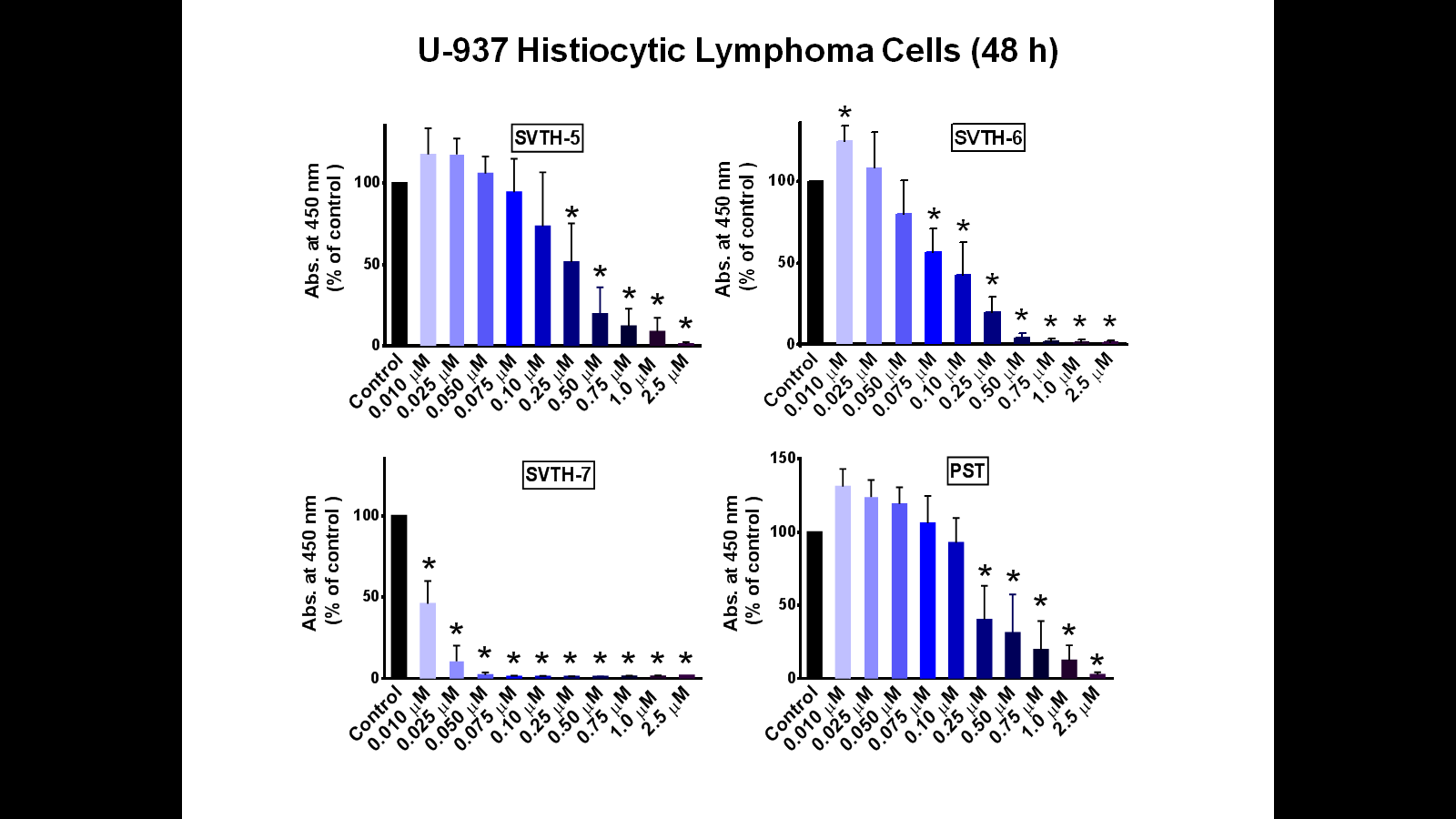


**Supplemental Figure 1e. PST Analogs & PST Decrease Viability of U-937 Leukemia Cells in a Dose Dependent Manner.** The WST-1 colorimetric assay was performed on U-937 lymphoma cells treated with the indicated concentrations of compounds for 48 hours. The WST-1 reagent was added and the absorbance of the processed WST-1 reagent formazan, used to quantify cell viability, was read at 450 nm and expressed as a percent of solvent control (DMSO). Values are expressed as mean ± SD from quadruplicates of 3 independent experiments. **p*<0.05 vs. solvent control (DMSO).


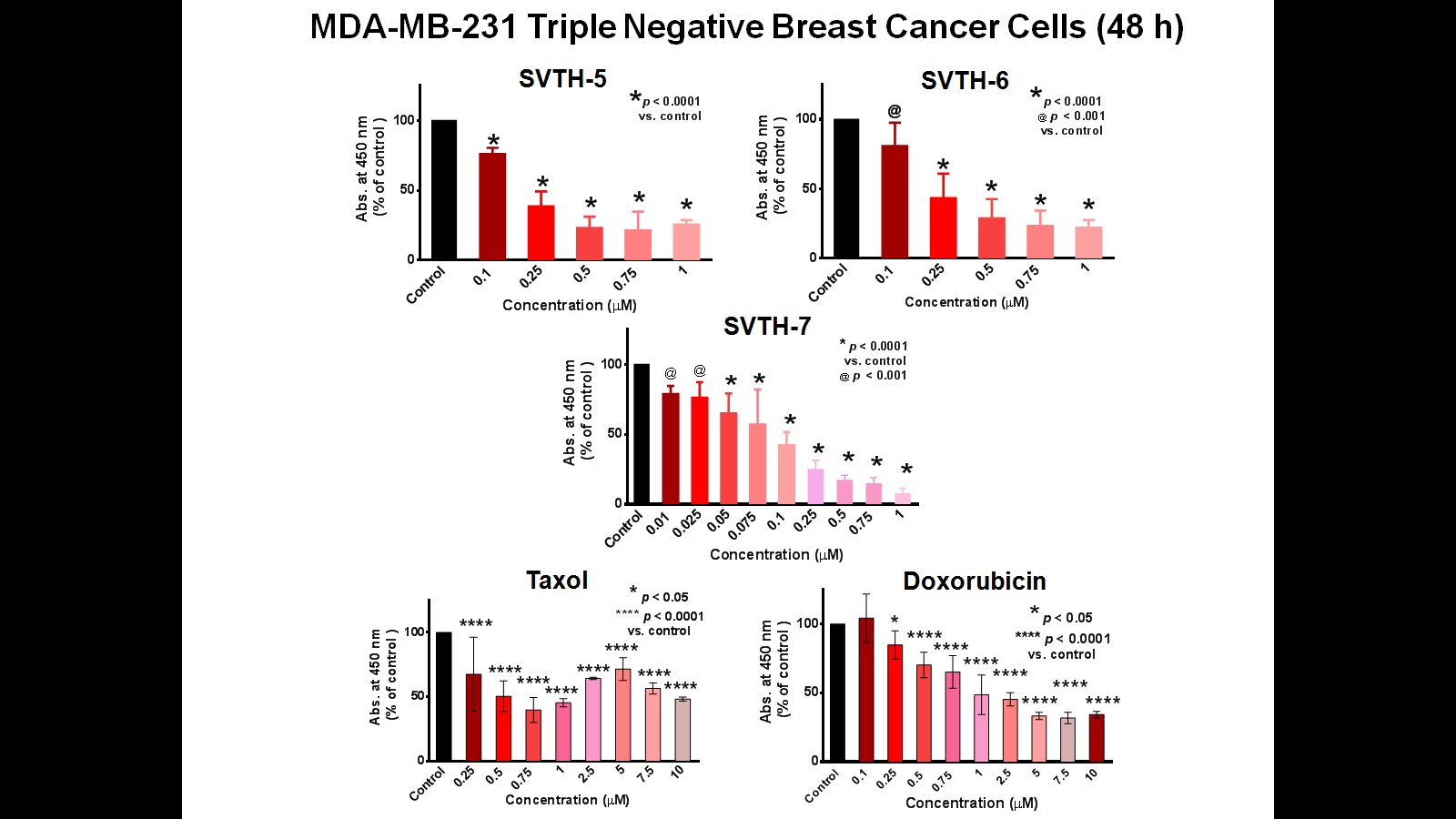


**Supplemental Figure 1f. PST Analogs Decrease Viability of MDA-MB-231 Triple Negative Breast Cancer Cells in a Dose Dependent Manner with Greater Efficacy than Taxol and Doxorubicin.** The WST-1 colorimetric assay was performed on MDA-MB-231 triple negative breast cancer cells treated with the indicated concentrations of compounds for 48 hours. The WST-1 reagent was added and the absorbance of the processed WST-1 reagent formazan, used to quantify cell viability, was read at 450 nm and expressed as a percent of solvent control (DMSO). Values are expressed as mean ± SD from quadruplicates of 3 independent experiments.


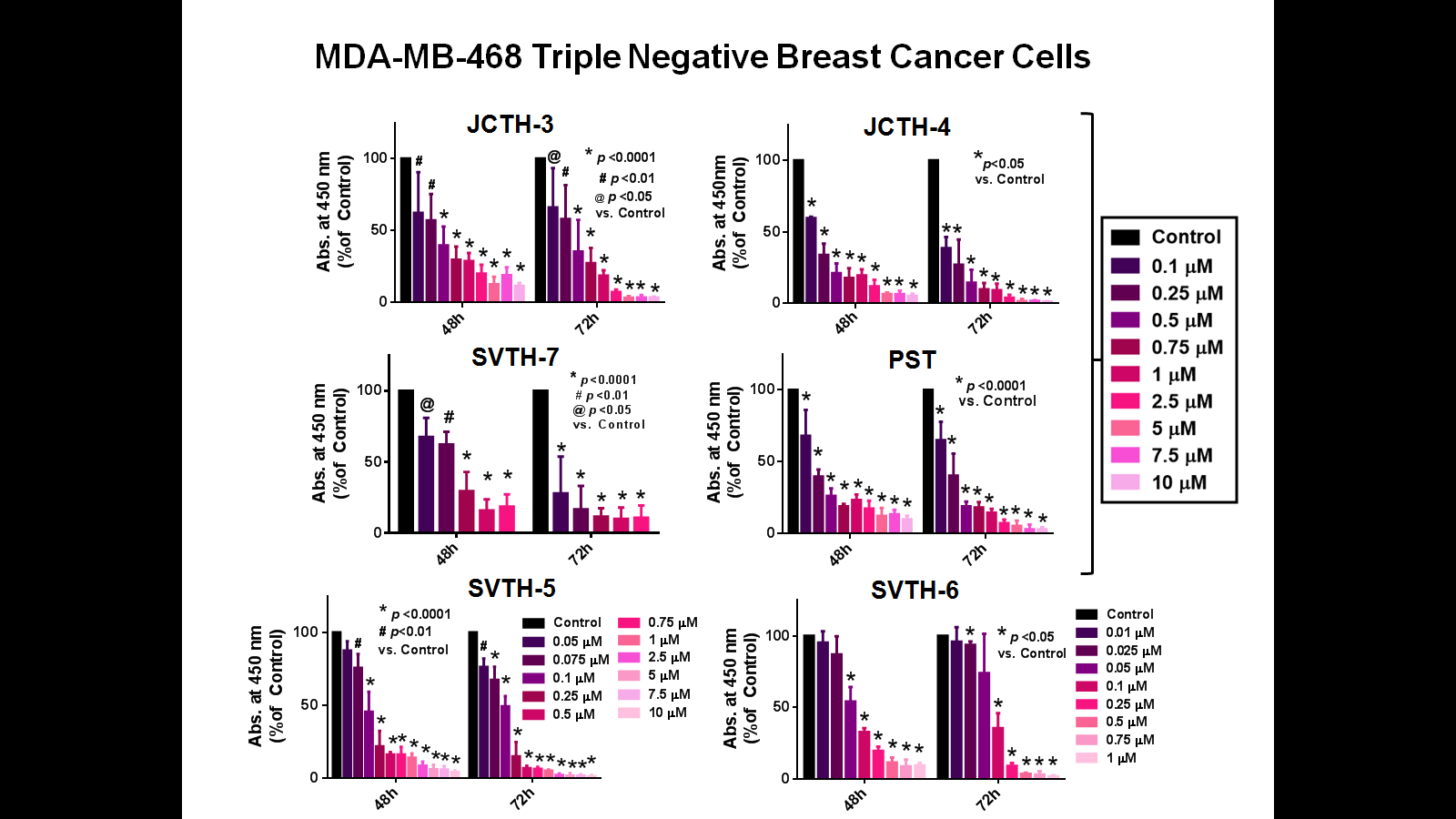


**Supplemental Figure 1g. PST Analogs & PST Decrease Viability of MDA-MB-468 Triple Negative Breast Cancer Cells in a Time and Dose Dependent Manner.** The WST-1 colorimetric assay was performed on MDA-MB-468 triple negative breast cancer cells treated with the indicated concentrations of compounds for the indicated durations. The WST-1 reagent was added and the absorbance of the processed WST-1 reagent formazan, used to quantify cell viability, was read at 450 nm and expressed as a percent of solvent control (DMSO). Values are expressed as mean ± SD from quadruplicates of 3 independent experiments.


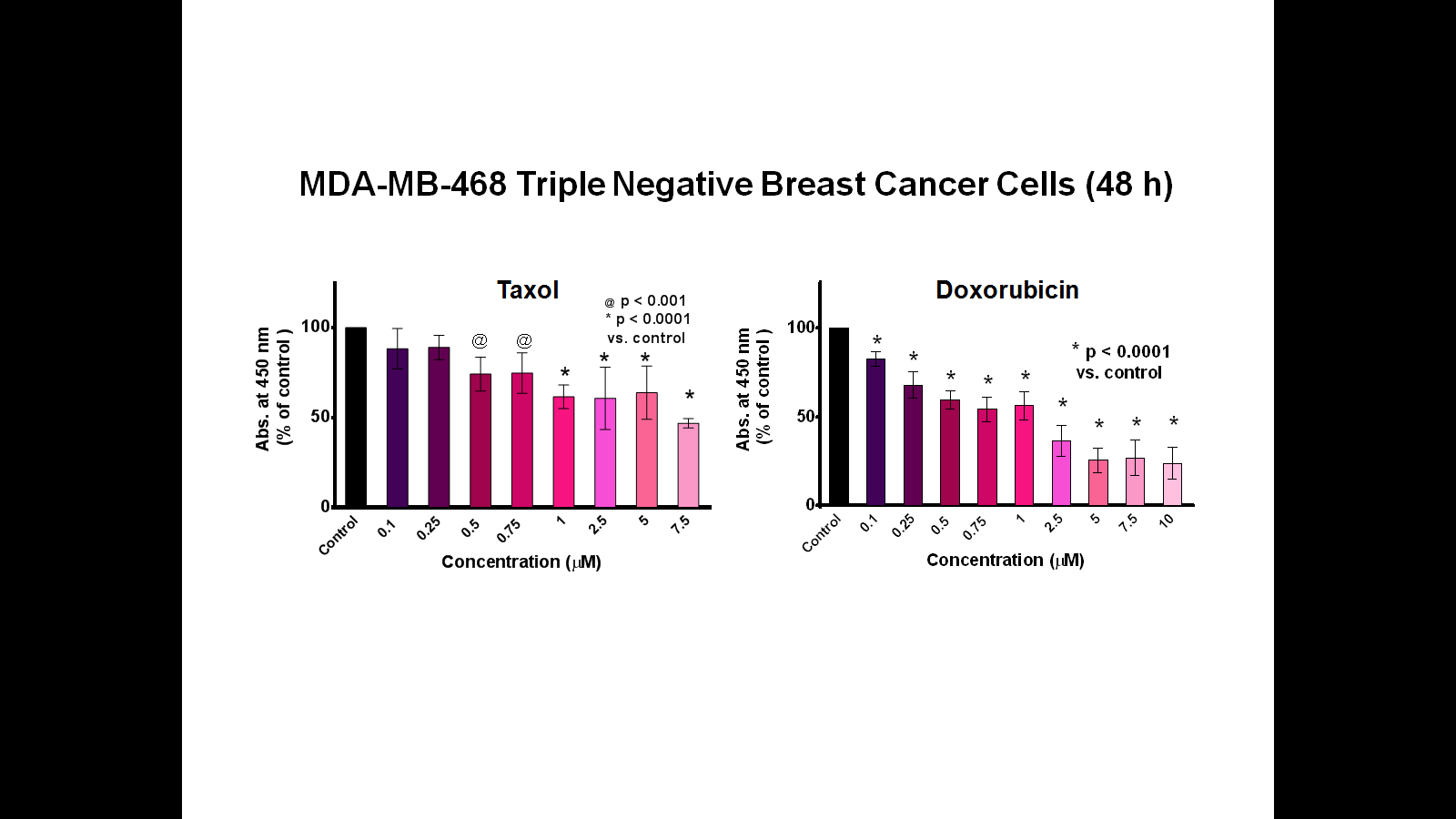


**Supplemental Figure 1h. Taxol and Doxorubicin Decrease Viability of MDA-MB-468 Triple Negative Breast Cancer Cells with Less Efficacy than PST Analogs.** The WST-1 colorimetric assay was performed on MDA-MB-468 triple negative breast cancer cells treated with the indicated concentrations of compounds for 48 hours. The WST-1 reagent was added and the absorbance of the processed WST-1 reagent formazan, used to quantify cell viability, was read at 450 nm and expressed as a percent of solvent control (DMSO). Values are expressed as mean ± SD from quadruplicates of 3 independent experiments.


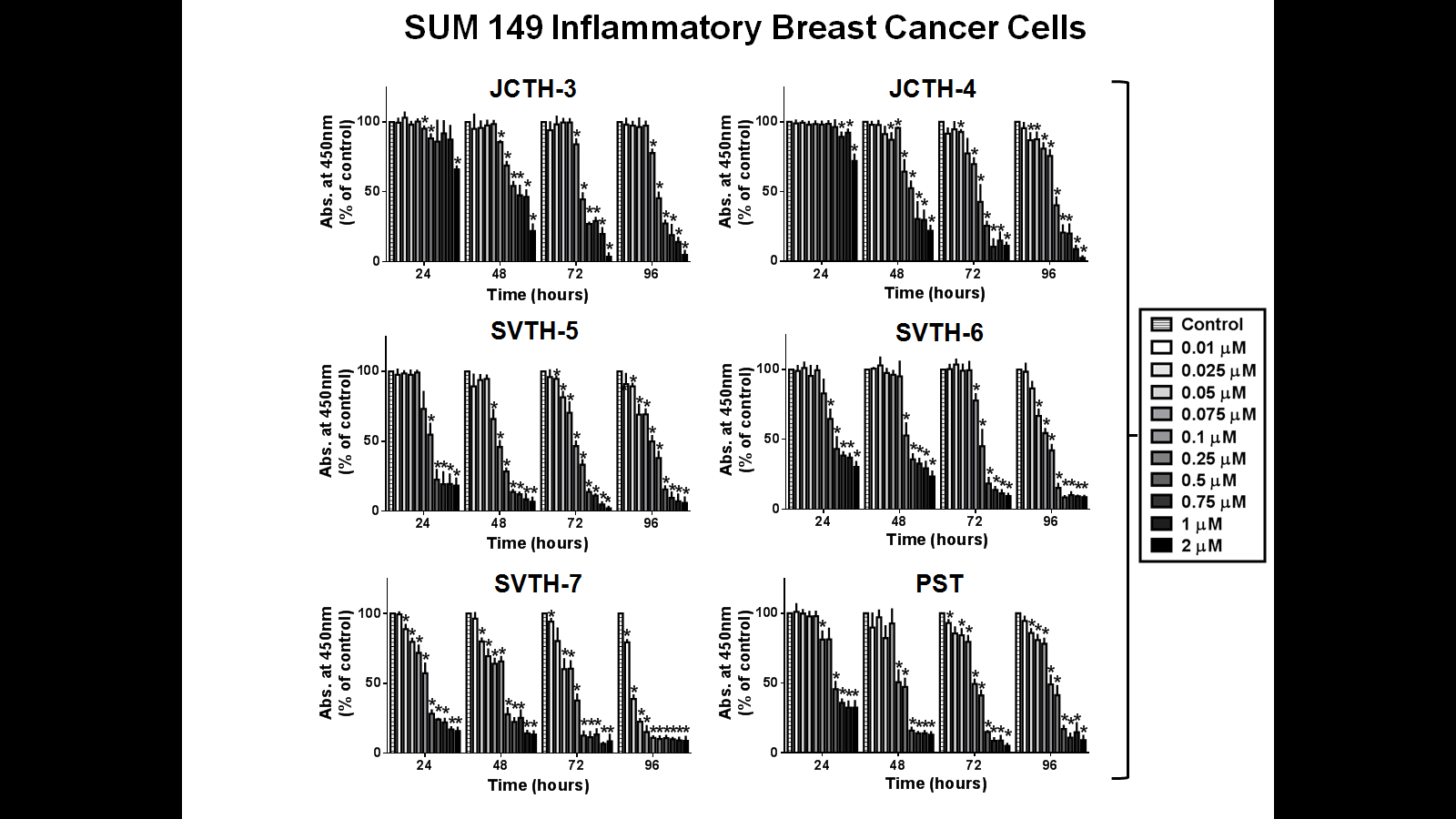


**Supplemental Figure 1i. PST Analogs & PST Decrease Viability of SUM 149 Breast Cancer Cells in a Time and Dose Dependent Manner.** The WST-1 colorimetric assay was performed on SUM 149 breast cancer cells treated with the indicated concentrations of compounds for the indicated durations. The WST-1 reagent was added and the absorbance of the processed WST-1 reagent formazan, used to quantify cell viability, was read at 450 nm and expressed as a percent of solvent control (DMSO). Values are expressed as mean ± SD from quadruplicates of 3 independent experiments. **p*<0.05 vs. solvent control (DMSO).


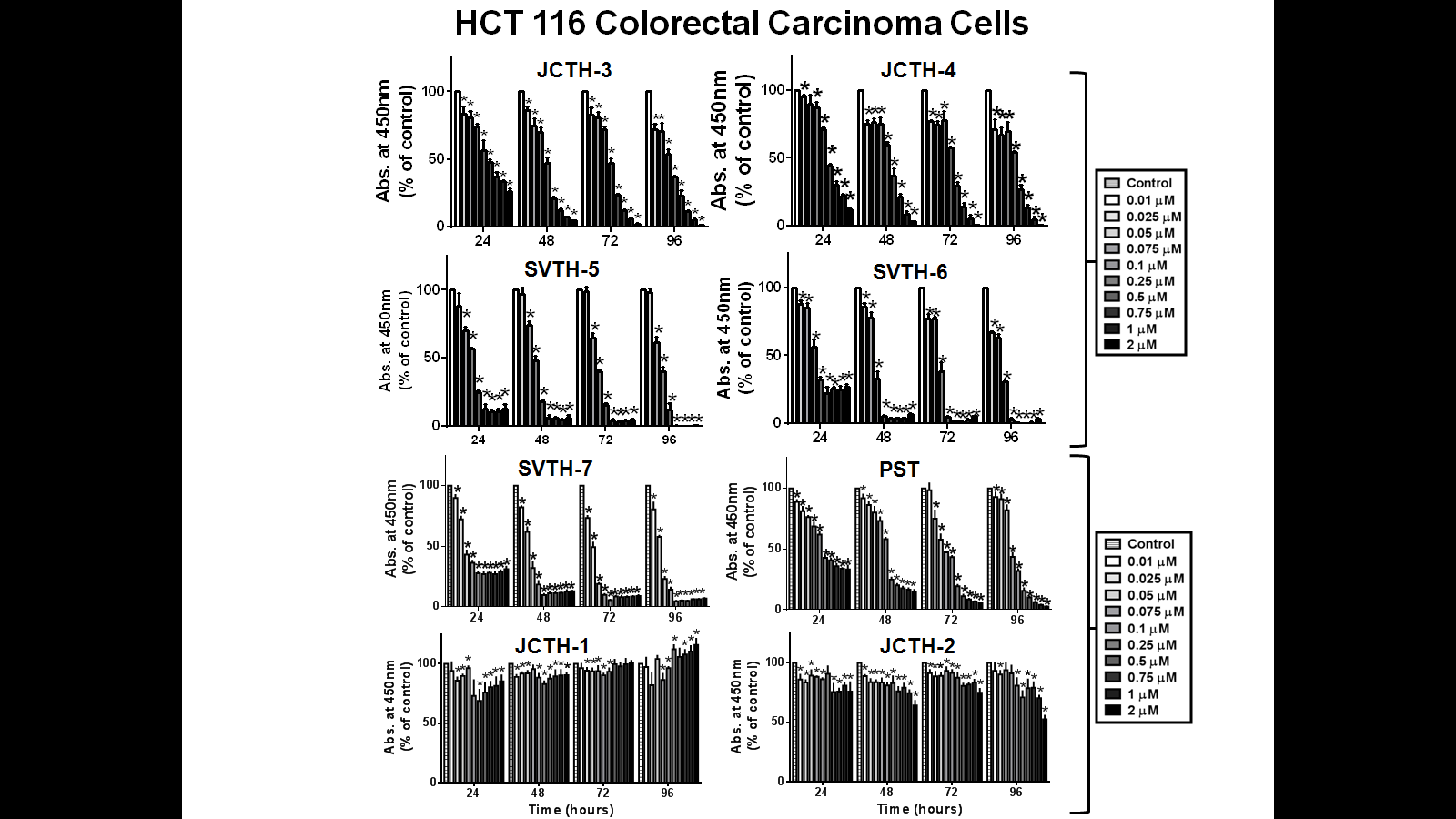


**Supplemental Figure 1j. PST Analogs & PST Decrease Viability of HCT 116 Colorectal Carcinoma Cells in a Time and Dose Dependent Manner.** The WST-1 colorimetric assay was performed on HCT 116 colorectal cells treated with the indicated concentrations of compounds for the indicated durations. The WST-1 reagent was added and the absorbance of the processed WST-1 reagent formazan, used to quantify cell viability, was read at 450 nm and expressed as a percent of solvent control (DMSO). Values are expressed as mean ± SD from quadruplicates of 3 independent experiments. **p*<0.05 vs. solvent control (DMSO).


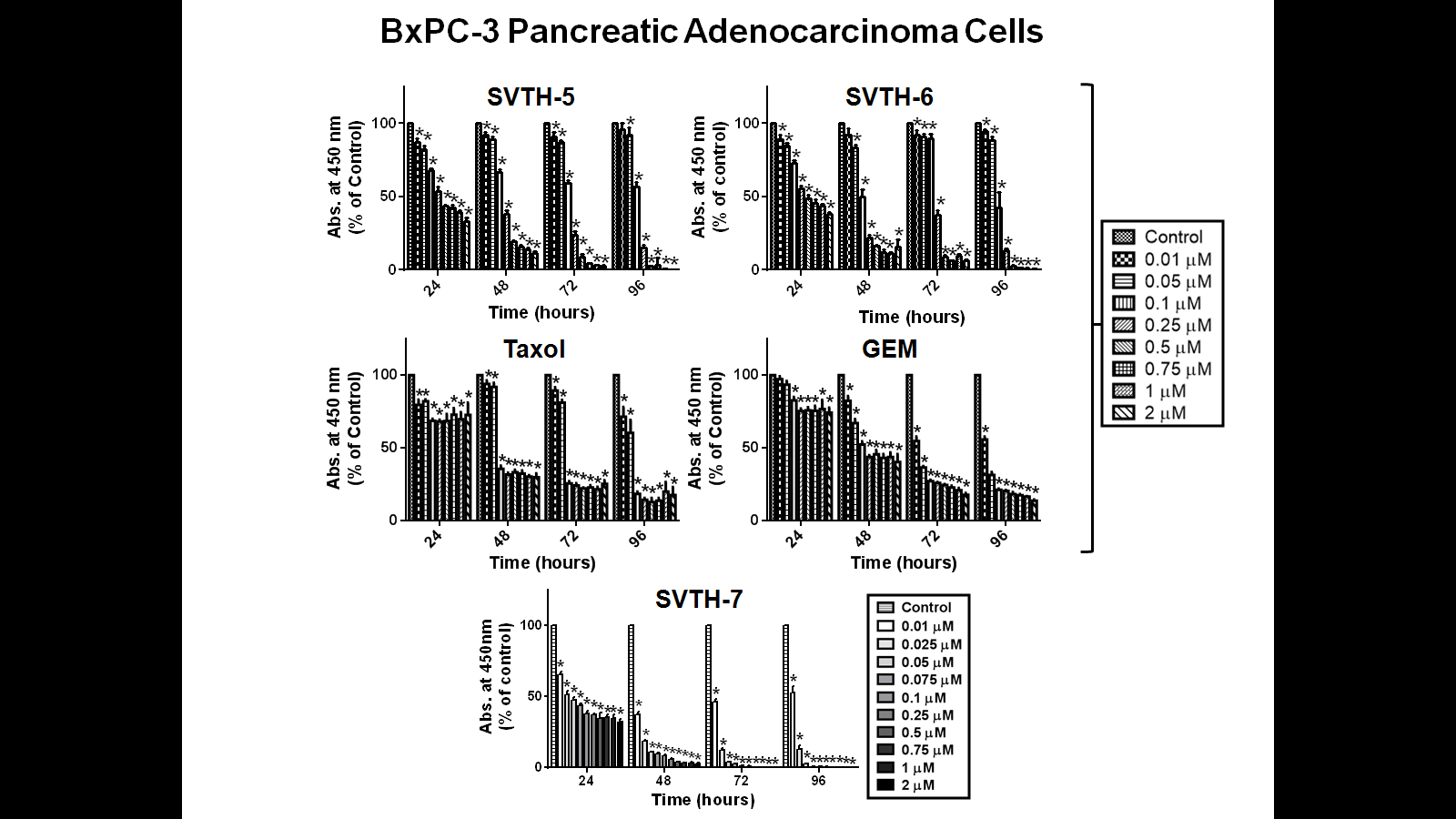


**Supplemental Figure 1k. PST Analogs Decrease Viability of BxPC-3 Pancreatic Adenocarcinoma Cells in a Time and Dose Dependent Manner with Greater Efficacy than Gemcitabine (GEM) and Taxol.** The WST-1 colorimetric assay was performed on BxPC-3 pancreatic adenocarcinoma cells treated with the indicated concentrations of compounds for the indicated durations. The WST-1 reagent was added and the absorbance of the processed WST-1 reagent formazan, used to quantify cell viability, was read at 450 nm and expressed as a percent of solvent control (DMSO). Values are expressed as mean ± SD from quadruplicates of 3 independent experiments. **p*<0.05 vs. solvent control (DMSO).


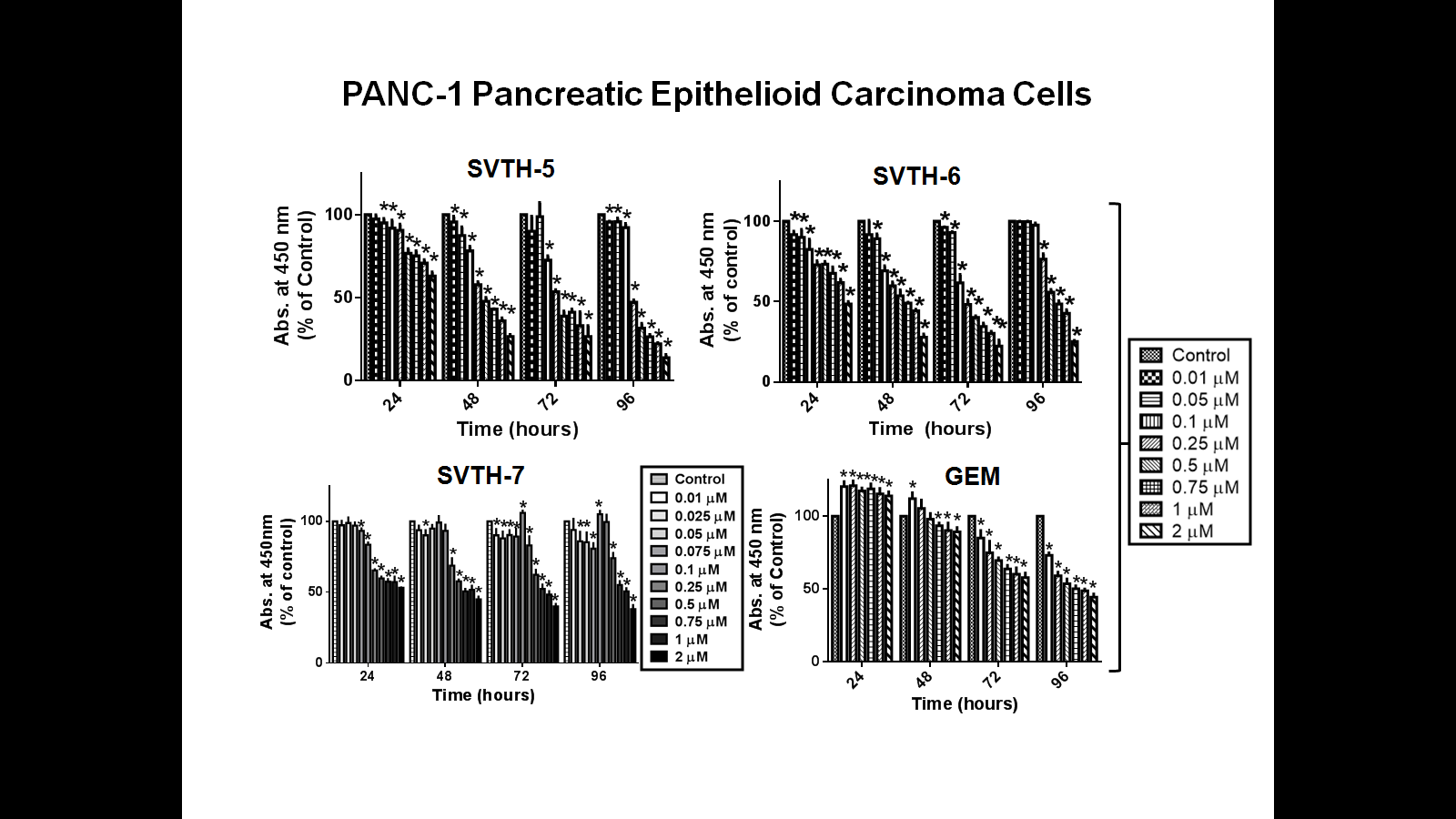


**Supplemental Figure 1l. PST Analogs Decrease Viability of PANC-1 Pancreatic Cancer Cells in a Time and Dose Dependent Manner with Greater Efficacy than Gemcitabine (GEM).** The WST-1 colorimetric assay was performed on PANC-1 pancreatic cancer cells treated with the indicated concentrations of compounds for the indicated durations. The WST-1 reagent was added and the absorbance of the processed WST-1 reagent formazan, used to quantify cell viability, was read at 450 nm and expressed as a percent of solvent control (DMSO). Values are expressed as mean ± SD from quadruplicates of 3 independent experiments. **p*<0.05 vs. solvent control (DMSO).


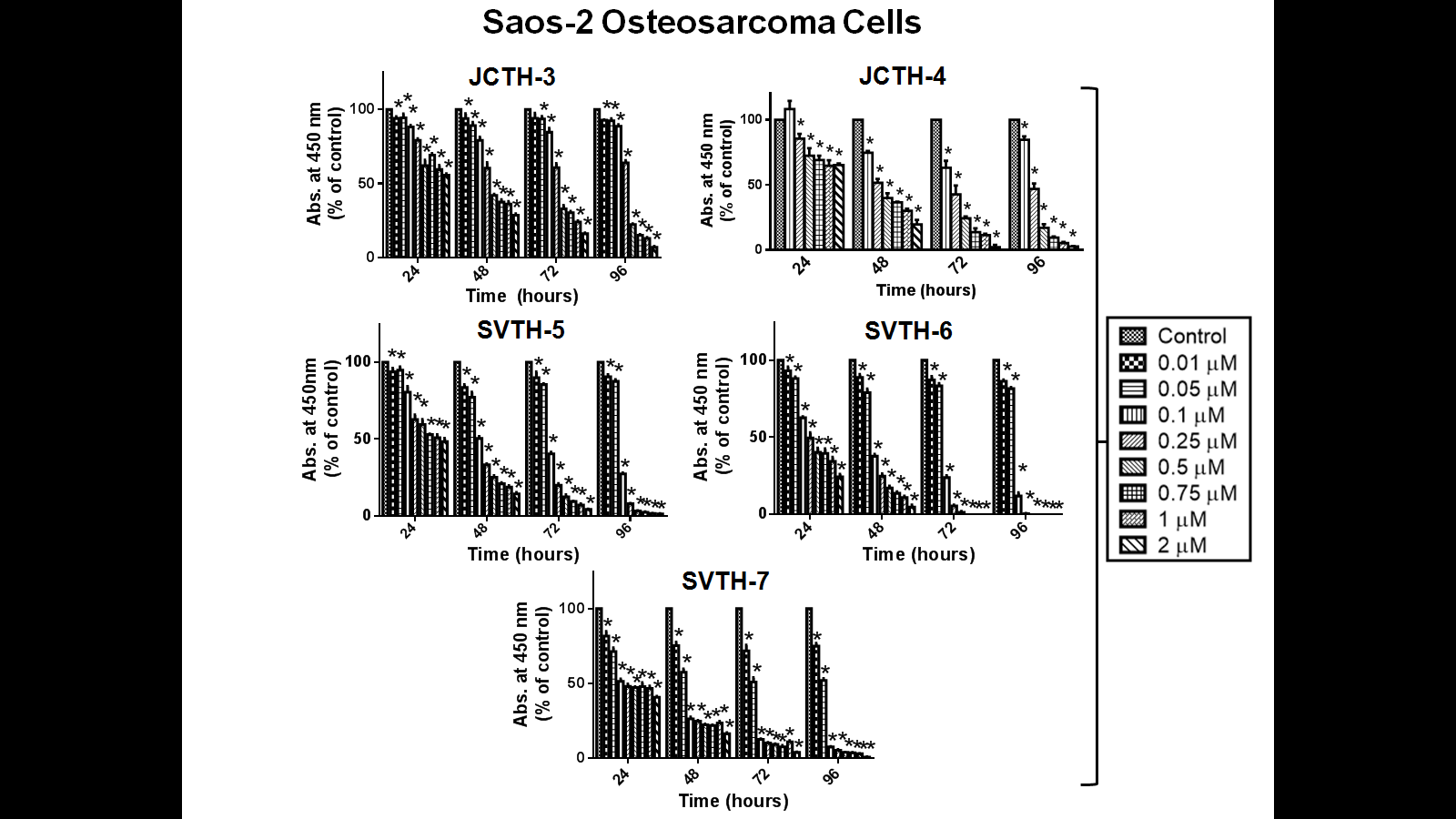


**Supplemental Figure 1m. PST Analogs Decrease Viability of Saos-2 Osteosarcoma Cells in a Time and Dose Dependent Manner.** The WST-1 colorimetric assay was performed on Saos-2 osteosarcoma cells treated with the indicated concentrations of compounds for the indicated durations. The WST-1 reagent was added and the absorbance of the processed WST-1 reagent formazan, used to quantify cell viability, was read at 450 nm and expressed as a percent of solvent control (DMSO). Values are expressed as mean ± SD from quadruplicates of 3 independent experiments. **p*<0.05 vs. solvent control (DMSO).


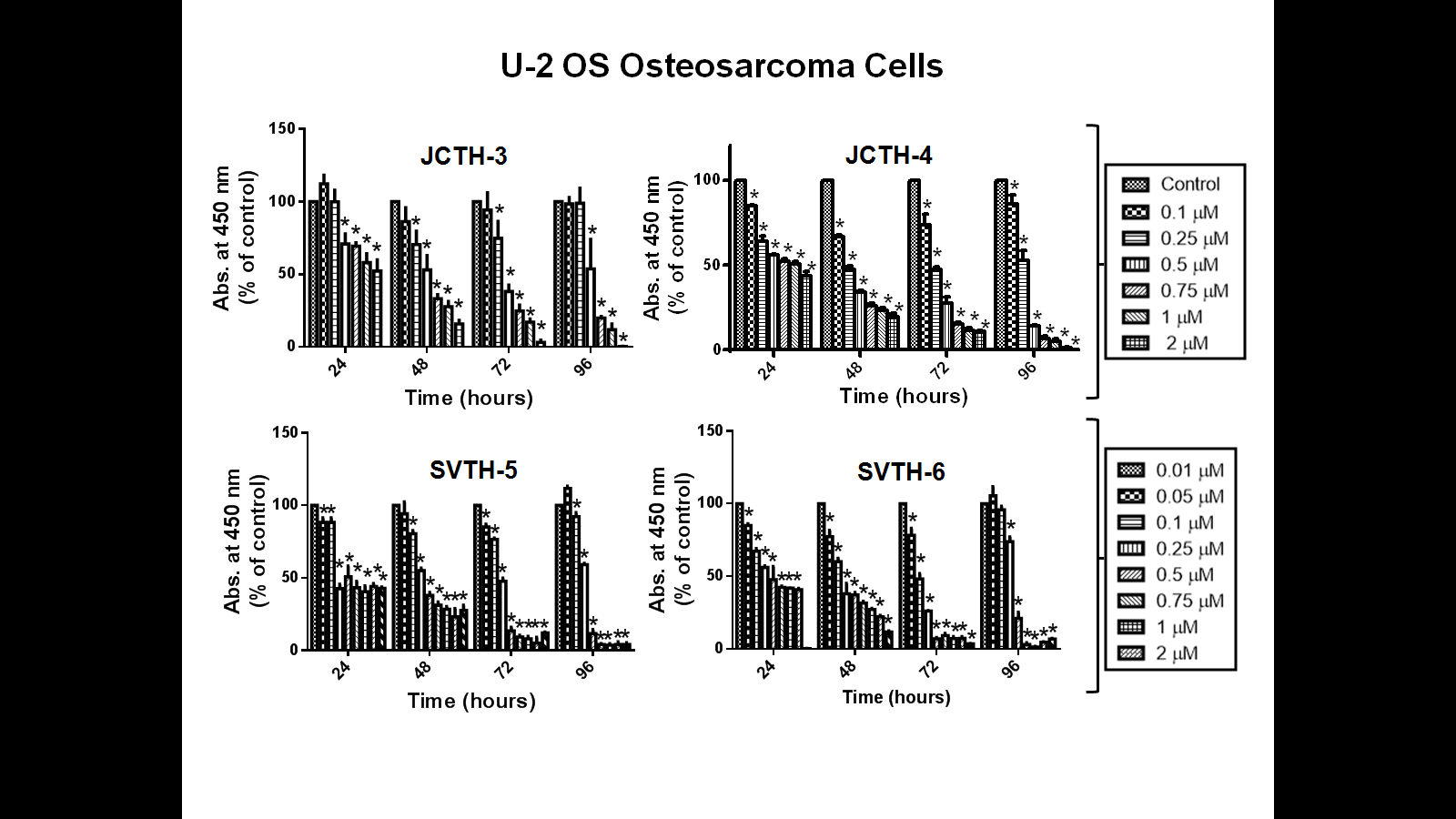


**Supplemental Figure 1n. PST Analogs Decrease Viability of U-2 OS Osteosarcoma Cells in a Time and Dose Dependent Manner.** The WST-1 colorimetric assay was performed on U-2 OS osteosarcoma cells treated with the indicated concentrations of compounds for the indicated durations. The WST-1 reagent was added and the absorbance of the processed WST-1 reagent formazan, used to quantify cell viability, was read at 450 nm and expressed as a percent of solvent control (DMSO). Values are expressed as mean ± SD from quadruplicates of 3 independent experiments. **p*<0.05 vs. solvent control (DMSO).


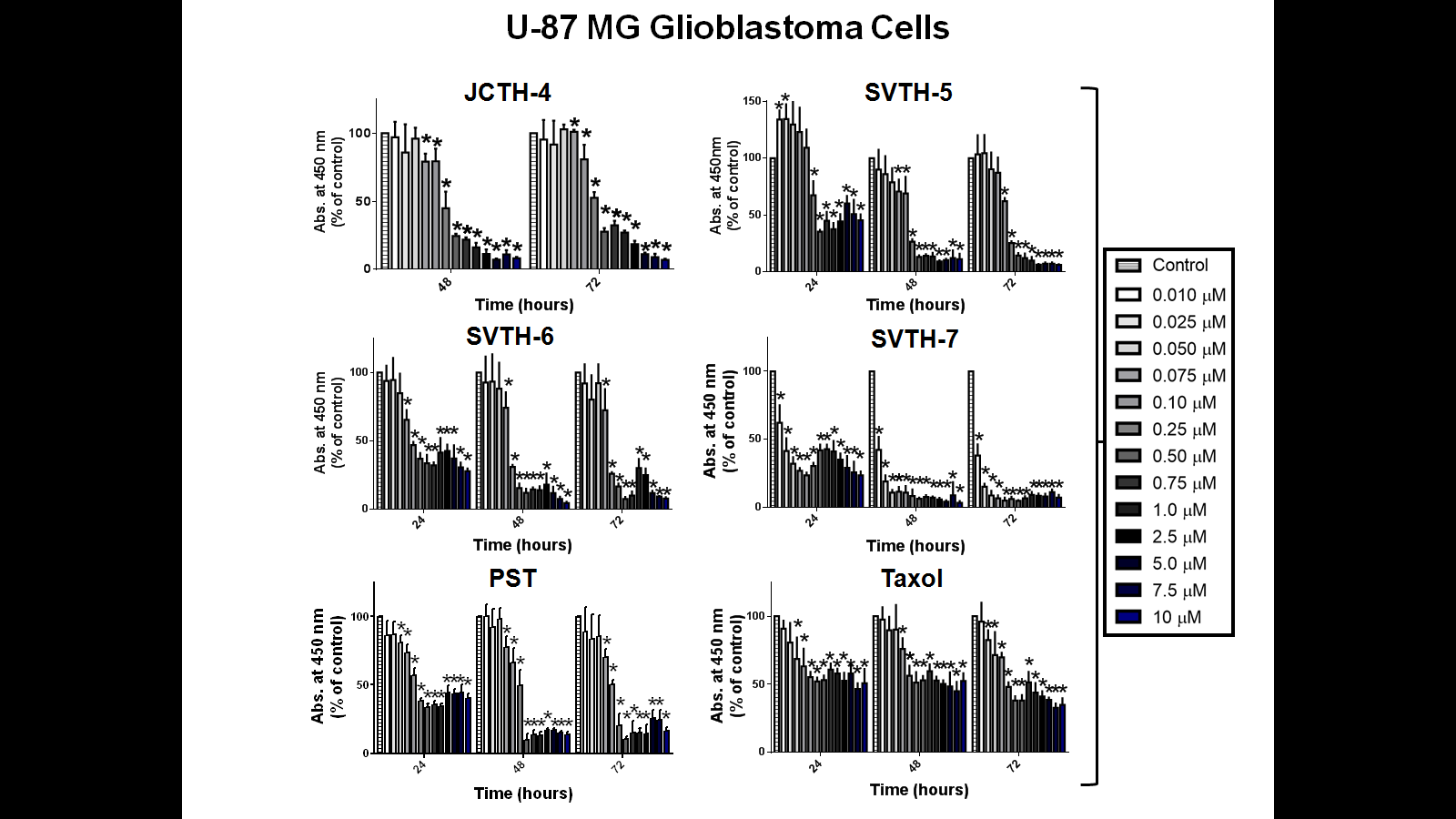


**Supplemental Figure 1o. PST Analogs & PST Decrease Viability of U-87 MG Glioblastoma Cells in a Time and Dose Dependent Manner with Greater Efficacy than Taxol.** The WST-1 colorimetric assay was performed on U-87 MG glioblastoma cells treated with the indicated concentrations of compounds for the indicated durations. The WST-1 reagent was added and the absorbance of the processed WST-1 reagent formazan, used to quantify cell viability, was read at 450 nm and expressed as a percent of solvent control (DMSO). Values are expressed as mean ± SD from quadruplicates of 3 independent experiments. **p*<0.05 vs. solvent control (DMSO).


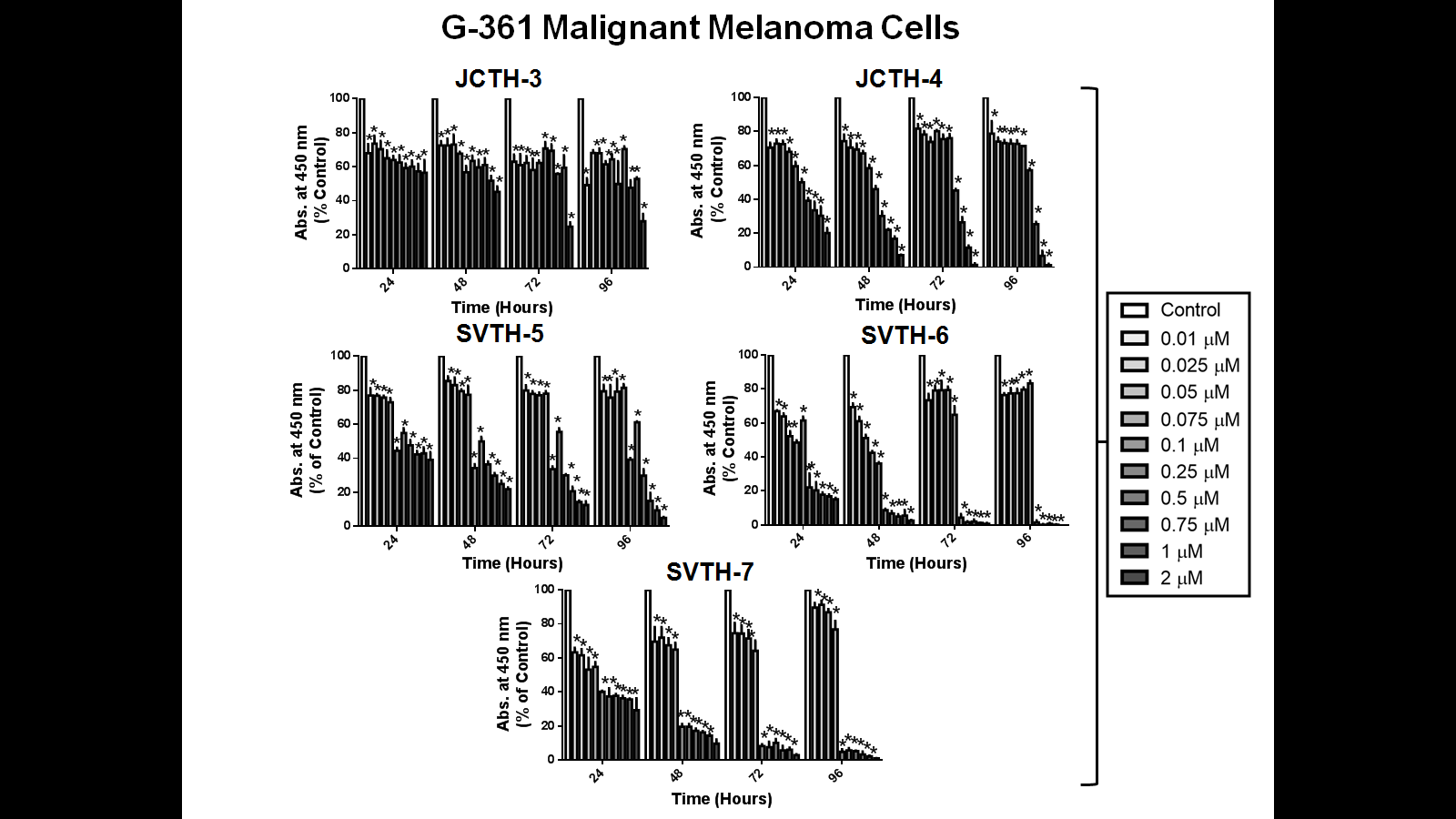


**Supplemental Figure 1p. PST Analogs Decrease Viability of G-361 Malignant Melanoma Cells in a Time and Dose Dependent Manner.** The WST-1 colorimetric assay was performed on G-361 malignant melanoma cells treated with the indicated concentrations of compounds for the indicated durations. The WST-1 reagent was added and the absorbance of the processed WST-1 reagent formazan, used to quantify cell viability, was read at 450 nm and expressed as a percent of solvent control (DMSO). Values are expressed as mean ± SD from quadruplicates of 3 independent experiments. **p*<0.05 vs. solvent control (DMSO).


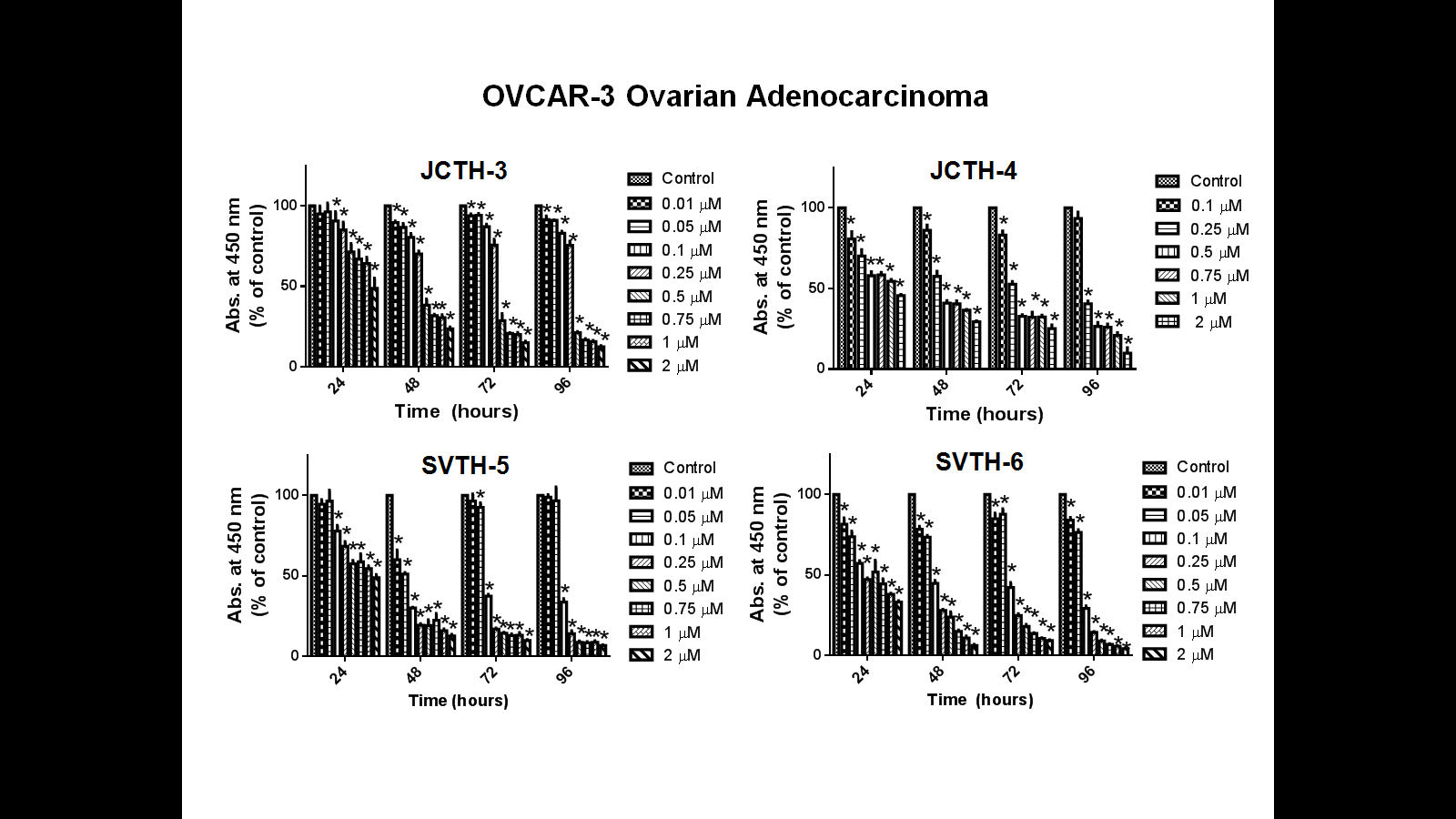


**Supplemental Figure 1q. PST Analogs Decrease Viability of OVCAR-3 Ovarian Adenocarcinoma Cells in a Time and Dose Dependent Manner.** The WST-1 colorimetric assay was performed on OVCAR-3 ovarian adenocarcinoma cells treated with the indicated concentrations of compounds for the indicated durations. The WST-1 reagent was added and the absorbance of the processed WST-1 reagent formazan, used to quantify cell viability, was read at 450 nm and expressed as a percent of solvent control (DMSO). Values are expressed as mean ± SD from quadruplicates of 3 independent experiments. **p*<0.05 vs. solvent control (DMSO).


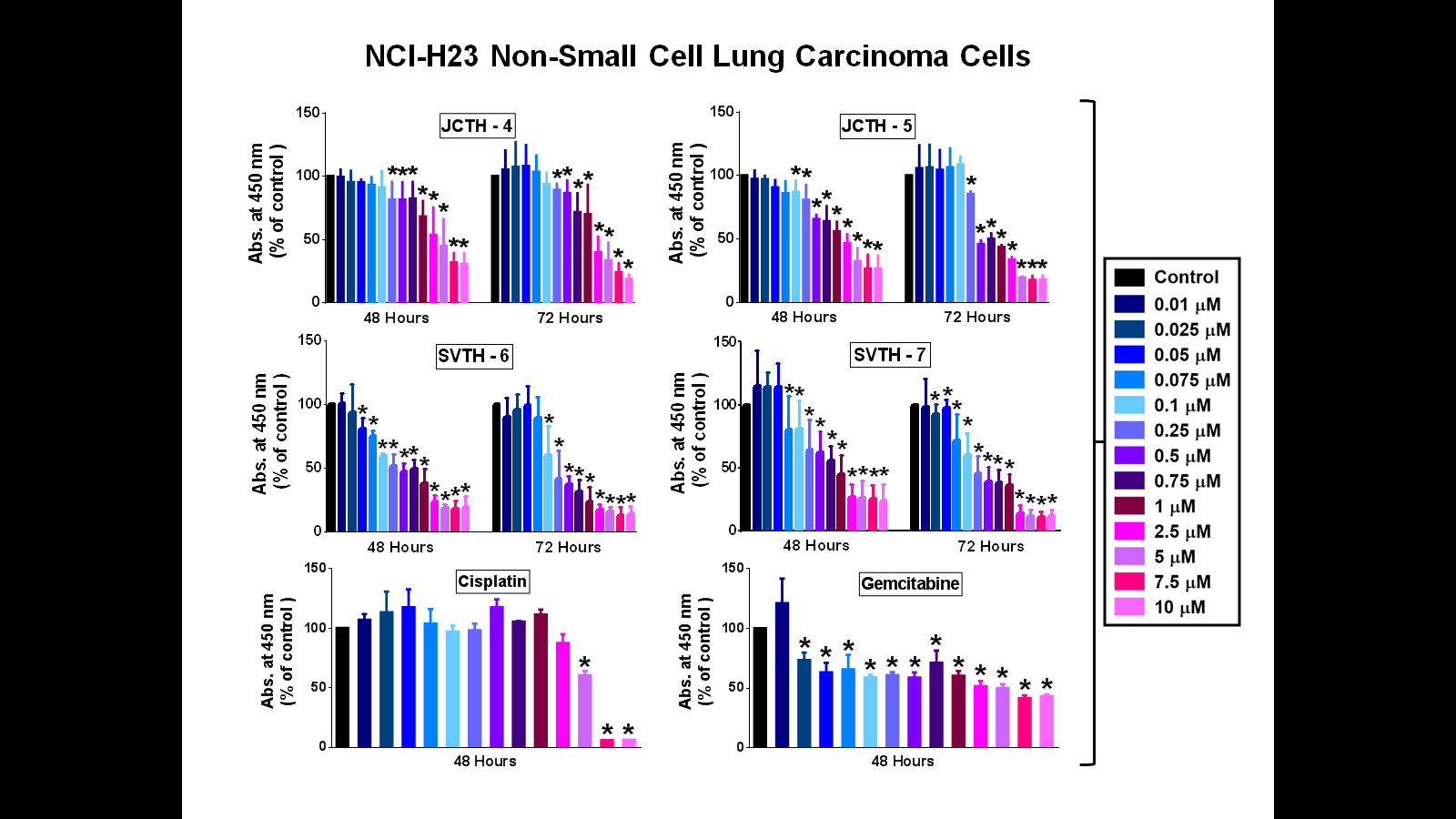


**Supplemental Figure 1r. PST Analogs Decrease Viability of NCI-H23 Non-Small Cell Lung Cancer Cells in a Time and Dose Dependent Manner with Greater Efficacy than Cisplatin and Gemcitabine.** The WST-1 colorimetric assay was performed on NCI-H23 non-small cell lung cancer cells treated with the indicated concentrations of compounds for the indicated durations. The WST-1 reagent was added and the absorbance of the processed WST-1 reagent formazan, used to quantify cell viability, was read at 450 nm and expressed as a percent of solvent control (DMSO). Values are expressed as mean ± SD from quadruplicates of 3 independent experiments. **p*<0.05 vs. solvent control (DMSO).


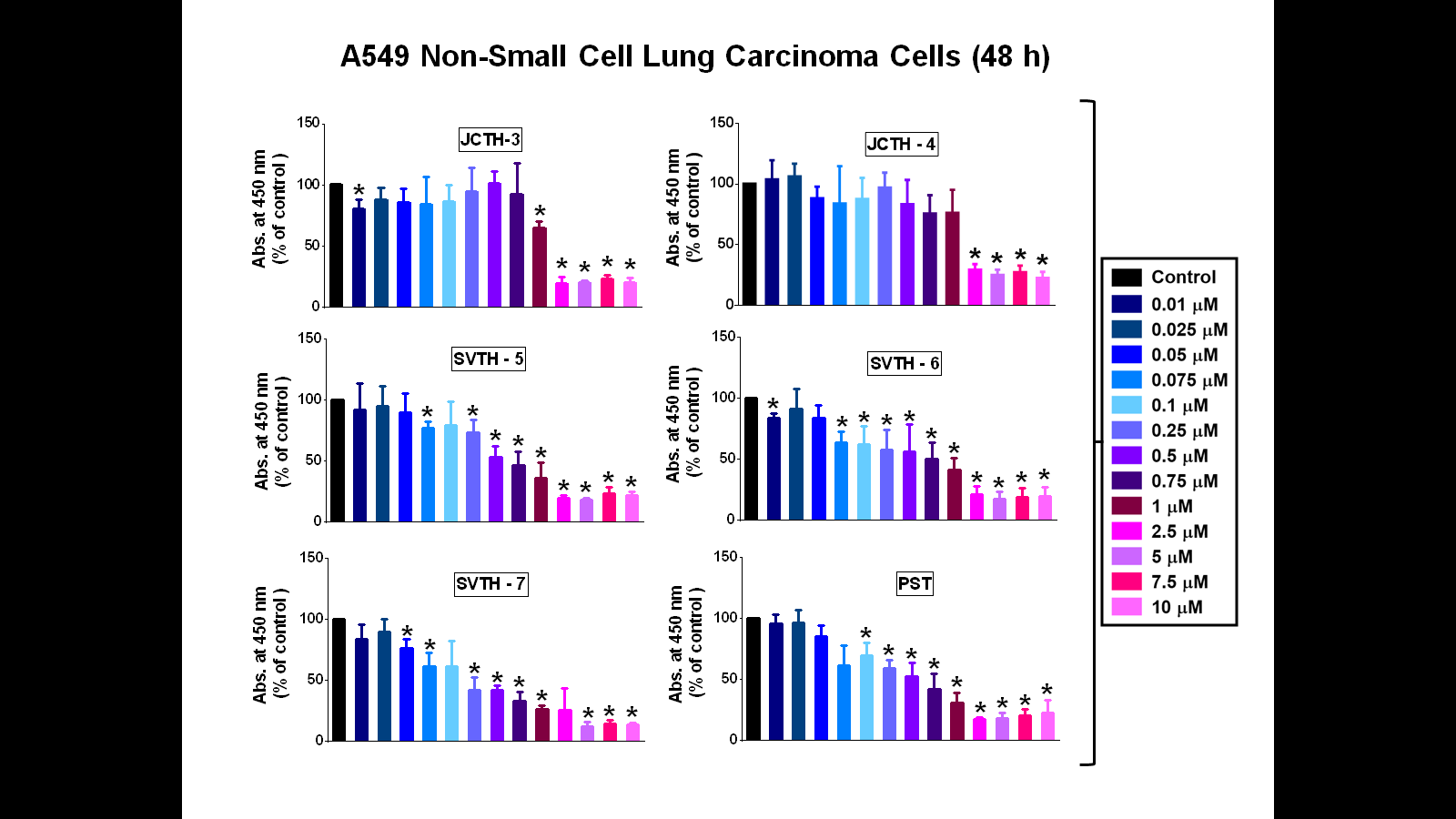


**Supplemental Figure 1s. PST Analogs Decrease Viability of A549 Non-Small Cell Lung Cancer Cells in a Dose Dependent Manner.** The WST-1 colorimetric assay was performed on A549 non-small cell lung cancer cells treated with the indicated concentrations of compounds for 48 hours. The WST-1 reagent was added and the absorbance of the processed WST-1 reagent formazan, used to quantify cell viability, was read at 450 nm and expressed as a percent of solvent control (DMSO). Values are expressed as mean ± SD from quadruplicates of 3 independent experiments. **p*<0.05 vs. solvent control (DMSO).


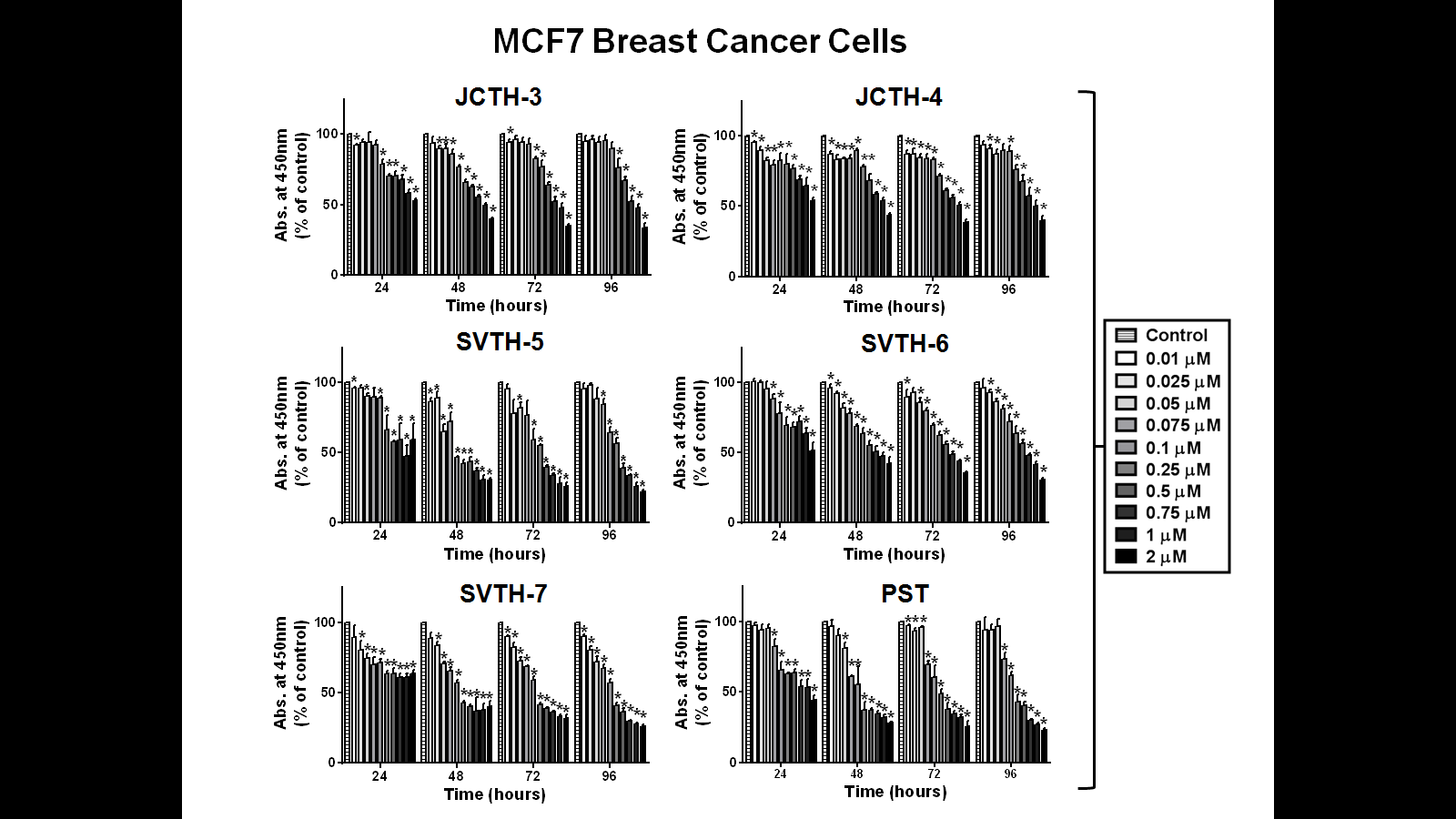


**Supplemental Figure 1t. PST Analogs & PST Decrease Viability of MCF7 Breast Cancer Cells in a Time and Dose Dependent Manner.** The WST-1 colorimetric assay was performed on MCF7 breast cancer cells treated with the indicated concentrations of compounds for the indicated durations. The WST-1 reagent was added and the absorbance of the processed WST-1 reagent formazan, used to quantify cell viability, was read at 450 nm and expressed as a percent of solvent control (DMSO). Values are expressed as mean ± SD from quadruplicates of 3 independent experiments. **p*<0.05 vs. solvent control (DMSO).


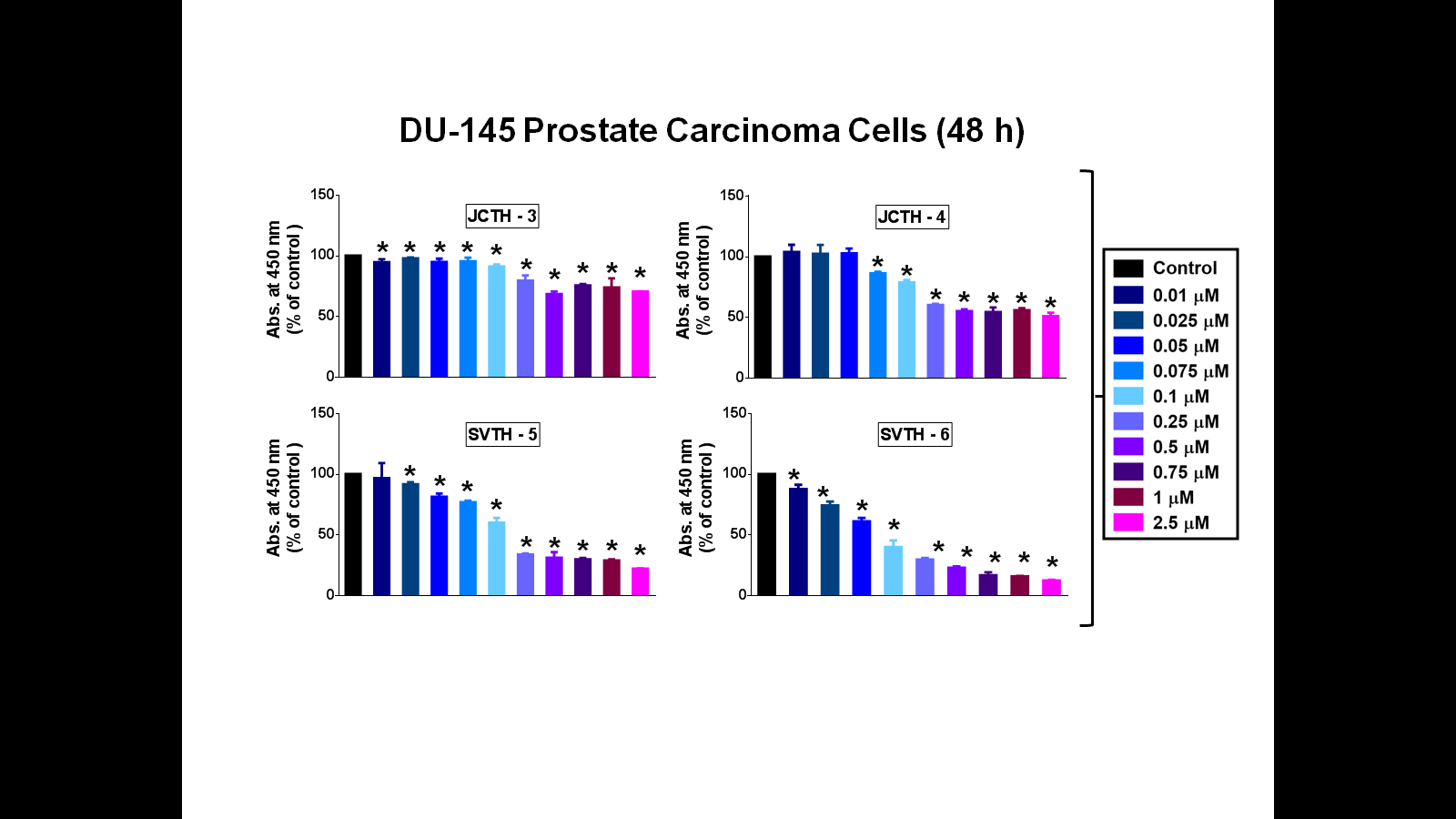


**Supplemental Figure 1u. PST Analogs Decrease Viability of DU-145 Prostate Carcinoma Cells in a Dose Dependent Manner.** The WST-1 colorimetric assay was performed on DU-145 prostate cancer cells treated with the indicated concentrations of compounds for 48 hours. The WST-1 reagent was added and the absorbance of the processed WST-1 reagent formazan, used to quantify cell viability, was read at 450 nm and expressed as a percent of solvent control (DMSO). Values are expressed as mean ± SD from quadruplicates of 3 independent experiments. **p*<0.05 vs. solvent control (DMSO).


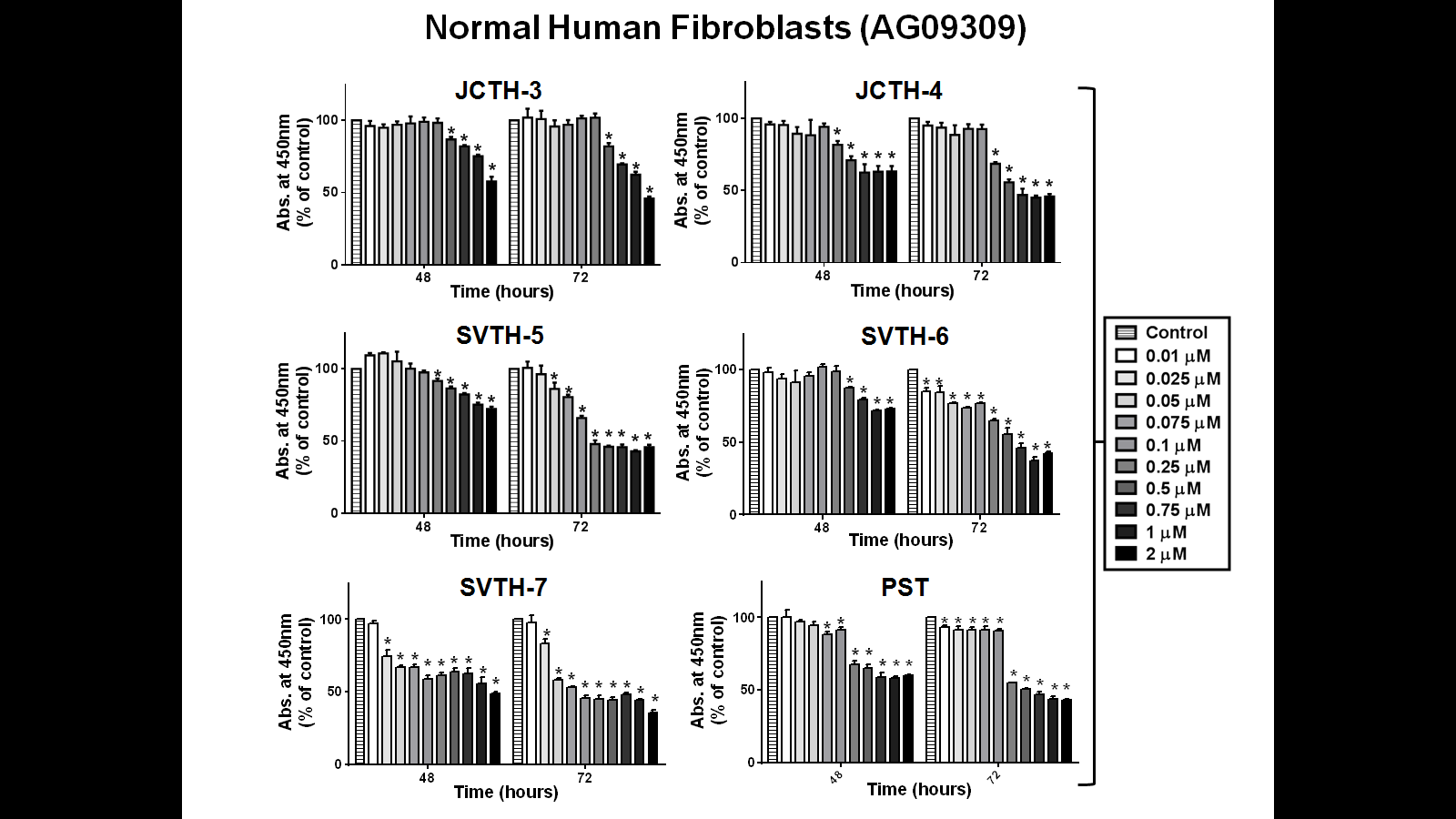


**Supplemental Figure 1v. PST Analogs & PST have Minimal Toxicity on AG09309 Normal Human Fibroblasts.** The WST-1 colorimetric assay was performed on AG09309 normal human fibroblasts treated with the indicated concentrations of compounds for 48 and 72 hours. The WST-1 reagent was added and the absorbance of the processed WST-1 reagent formazan, used to quantify cell viability, was read at 450 nm and expressed as a percent of solvent control (DMSO). Values are expressed as mean ± SD from quadruplicates of 3 independent experiments. **p*<0.05 vs. solvent control (DMSO).


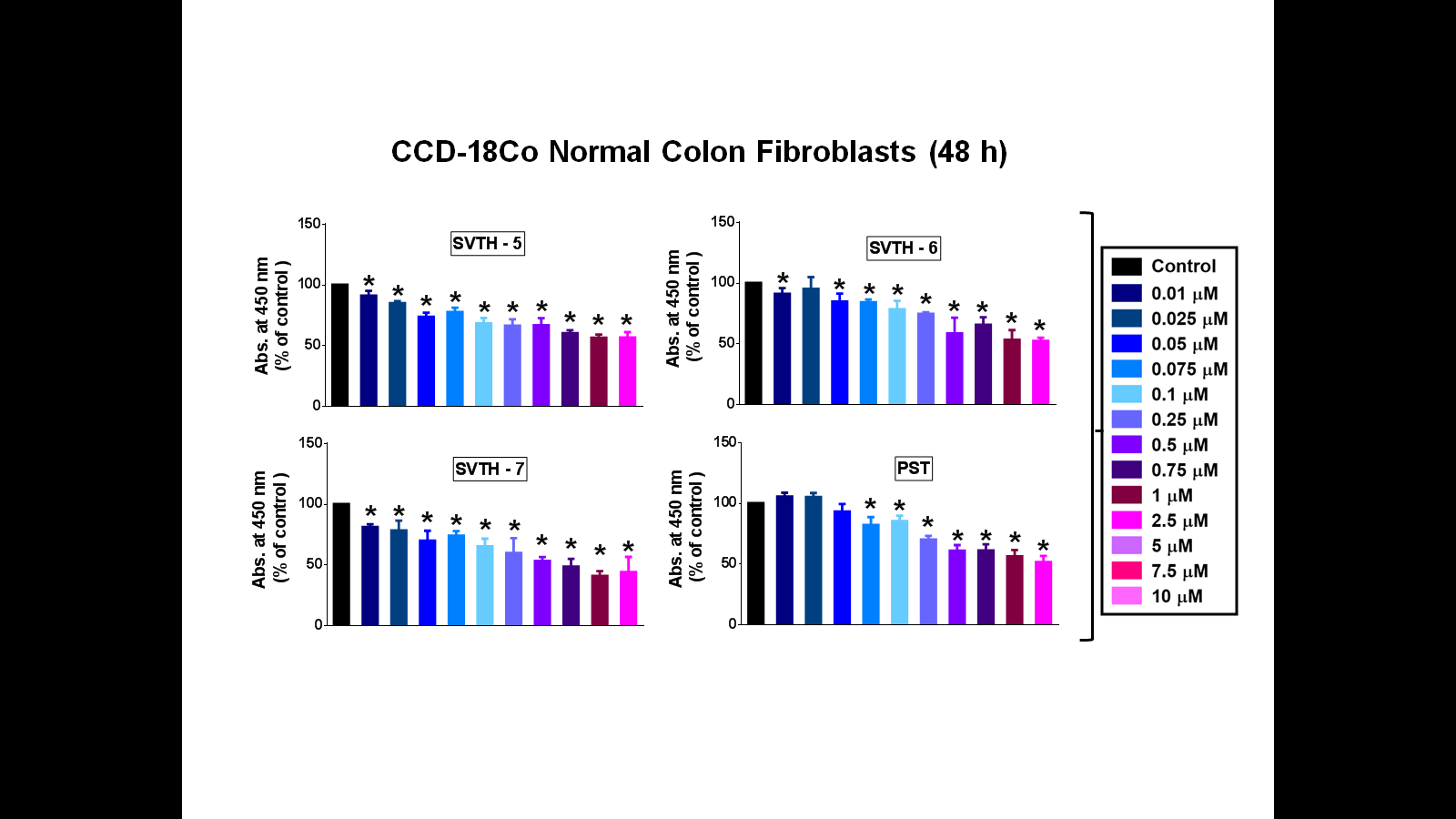


**Supplemental Figure 1w. PST Analogs & PST have Minimal Toxicity on CCD-18Co Normal Colon Fibroblasts.** The WST-1 colorimetric assay was performed on CCD-18Co normal human colon fibroblasts treated with the indicated concentrations of compounds for 48 hours. The WST-1 reagent was added and the absorbance of the processed WST-1 reagent formazan, used to quantify cell viability, was read at 450 nm and expressed as a percent of solvent control (DMSO). Values are expressed as mean ± SD from quadruplicates of 3 independent experiments. **p*<0.05 vs. solvent control (DMSO).


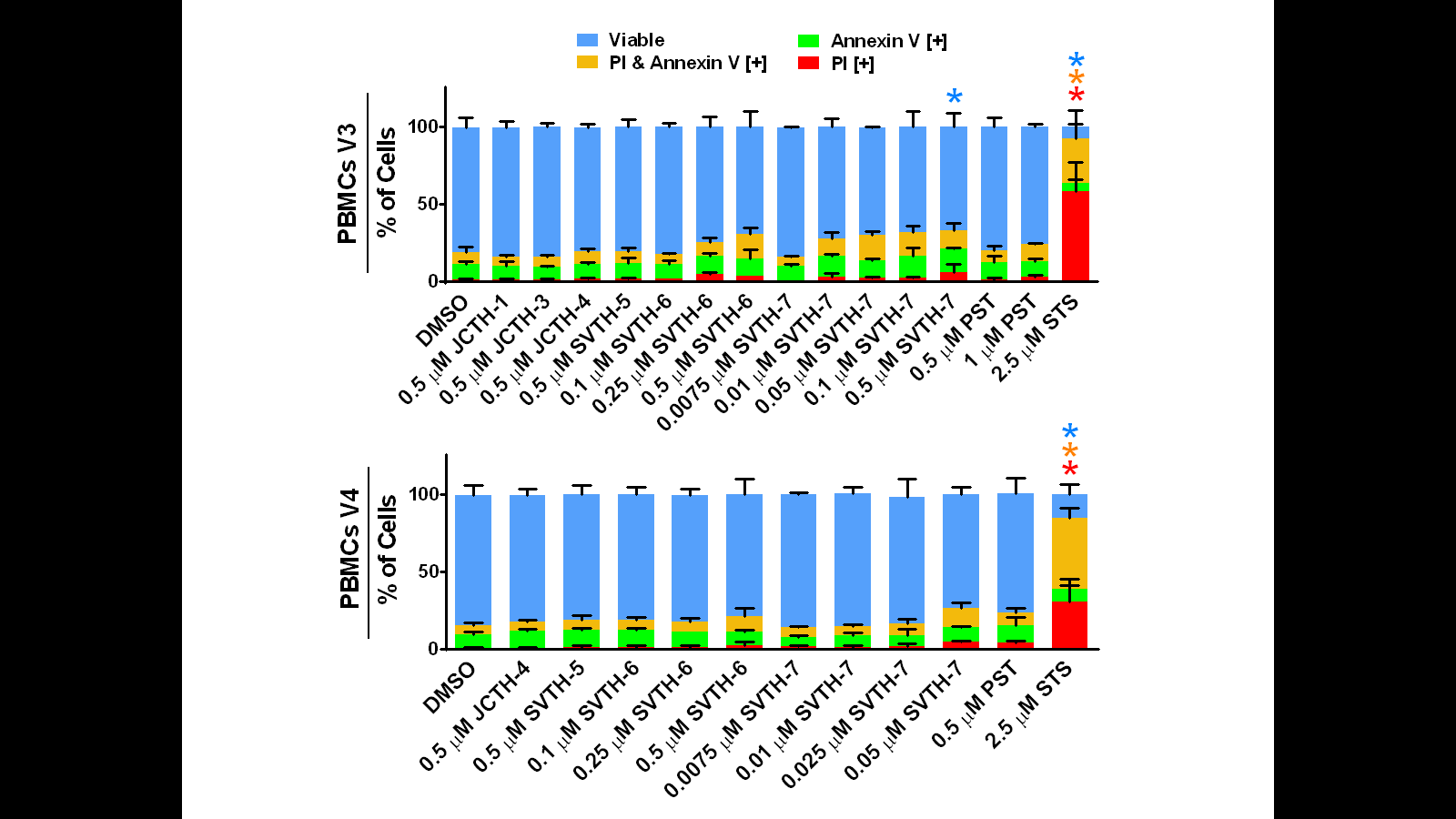


**Supplemental Figure 2a. PST Analogs & PST Induce Apoptosis Selectively in Cancer Cells: Minimal Induction of Apoptosis was Induced in Non-Cancerous Peripheral Blood Mononuclear Cells.** Annexin V binding and PI staining of peripheral blood mononuclear cells from healthy volunteer 3 (PBMCs V3) and 4 (PBMCs V4) treated for 48 hours was monitored with image-based cytometry. Values are expressed as mean ± SD from at least 3 independent experiments. **p*<0.01 vs. DMSO control.


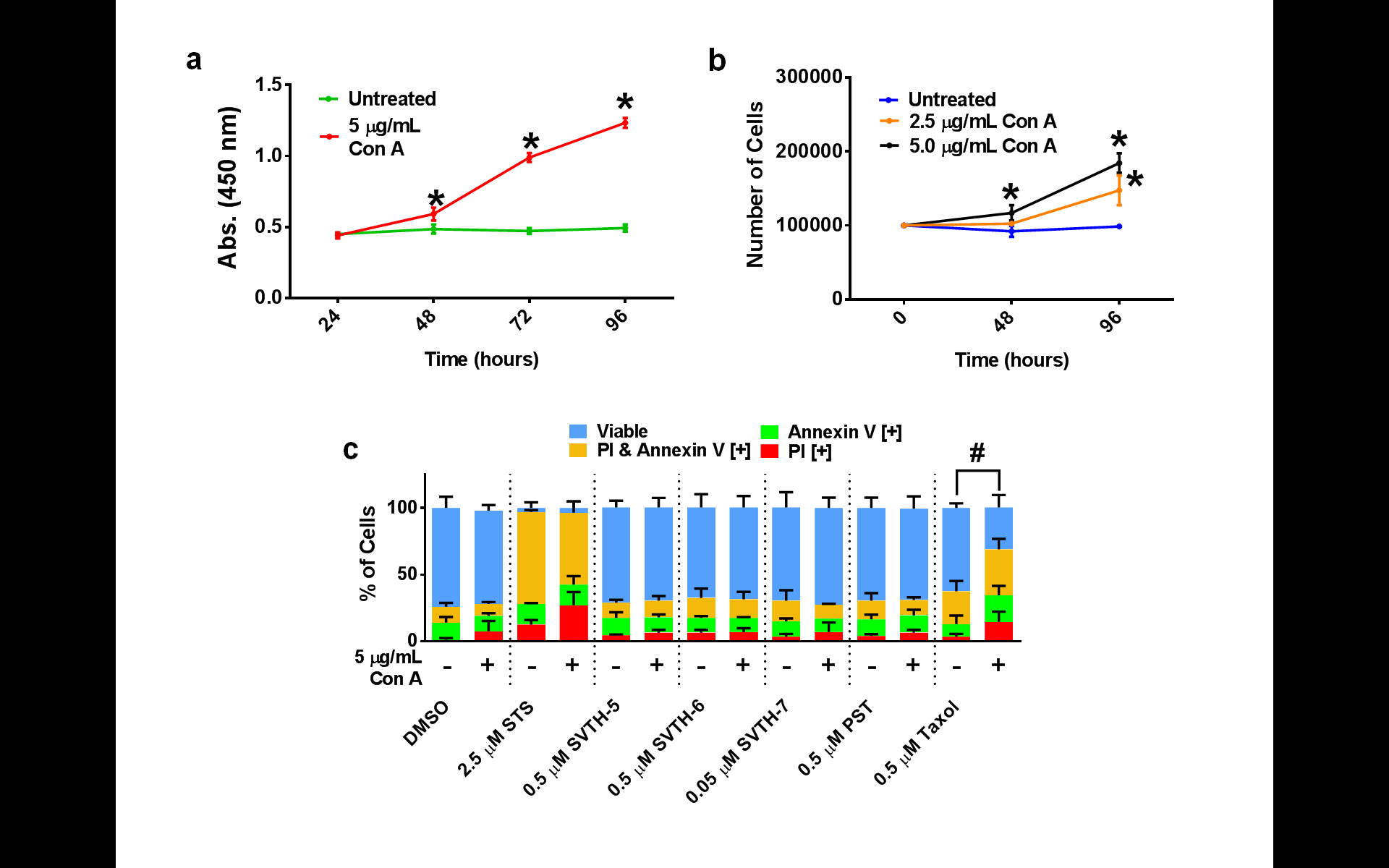


**Supplemental Figure 2b. PST Analogs & PST Induce Apoptosis Selectively in Cancer Cells Independent of Rate of Cell Division: Minimal Induction of Apoptosis was Induced in Actively Dividing Non-Cancerous Peripheral Blood Mononuclear Cells.** Peripheral blood mononuclear cells from healthy volunteer 1 (PBMCs V1) were treated with concanavalin a (Con A), a known inducer of proliferation of peripheral blood mononuclear cells. Time kinetics of PBMCs V1 treated with Con A with **(a)** the WST-1 assay and **(b)** cell counting using the Tali Image-based Cytometer. **p*<0.01 vs. control. After it was established that these cells were actively dividing at 48 hours, PBMCs V1 were treated with 5 μg/mL Con A for 48 hours, and then treated with PST, PST analogs, staurosporine (STS), and Taxol for an additional 48 hours. Annexin V binding and PI staining was monitored with image-based cytometry. Values are expressed as mean ± SD from at least 3 independent experiments. Only Taxol was observed to have an increase in cell death with Con A treatment. #*p*<0.01 vs. respective treatment group without Con A treatment (comparison of viable cells only indicated in blue).


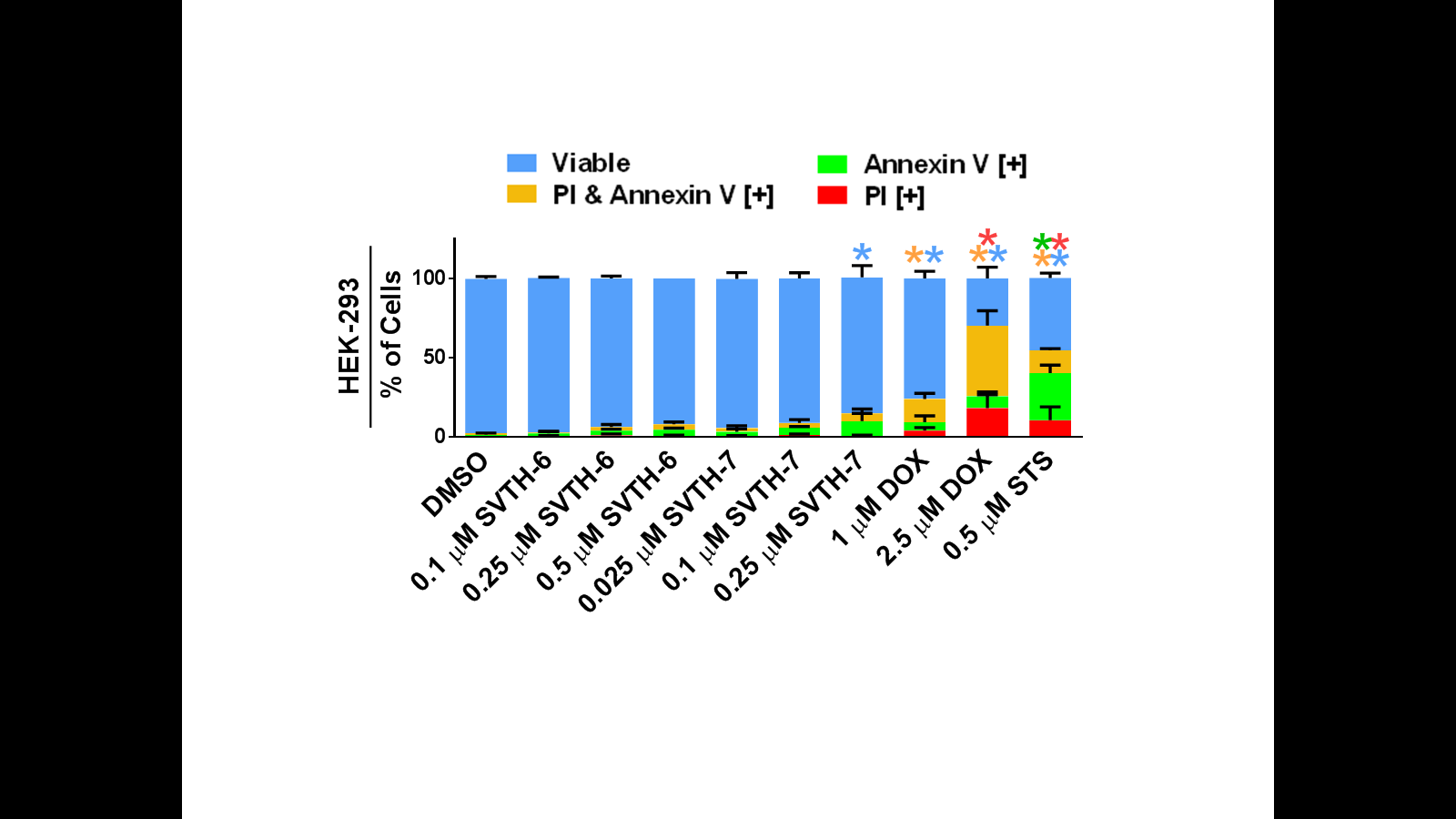


**Supplemental Figure 2c. PST Analogs & PST Induce Apoptosis Selectively in Cancer Cells: Minimal Induction of Apoptosis was Induced in HEK 293 Human Embryonic Kidney Cells.** Annexin V binding and PI staining of HEK 293 cells treated for 48 hours was monitored with image-based cytometry. Values are expressed as mean ± SD from at least 3 independent experiments. **p*<0.01 vs. DMSO control.


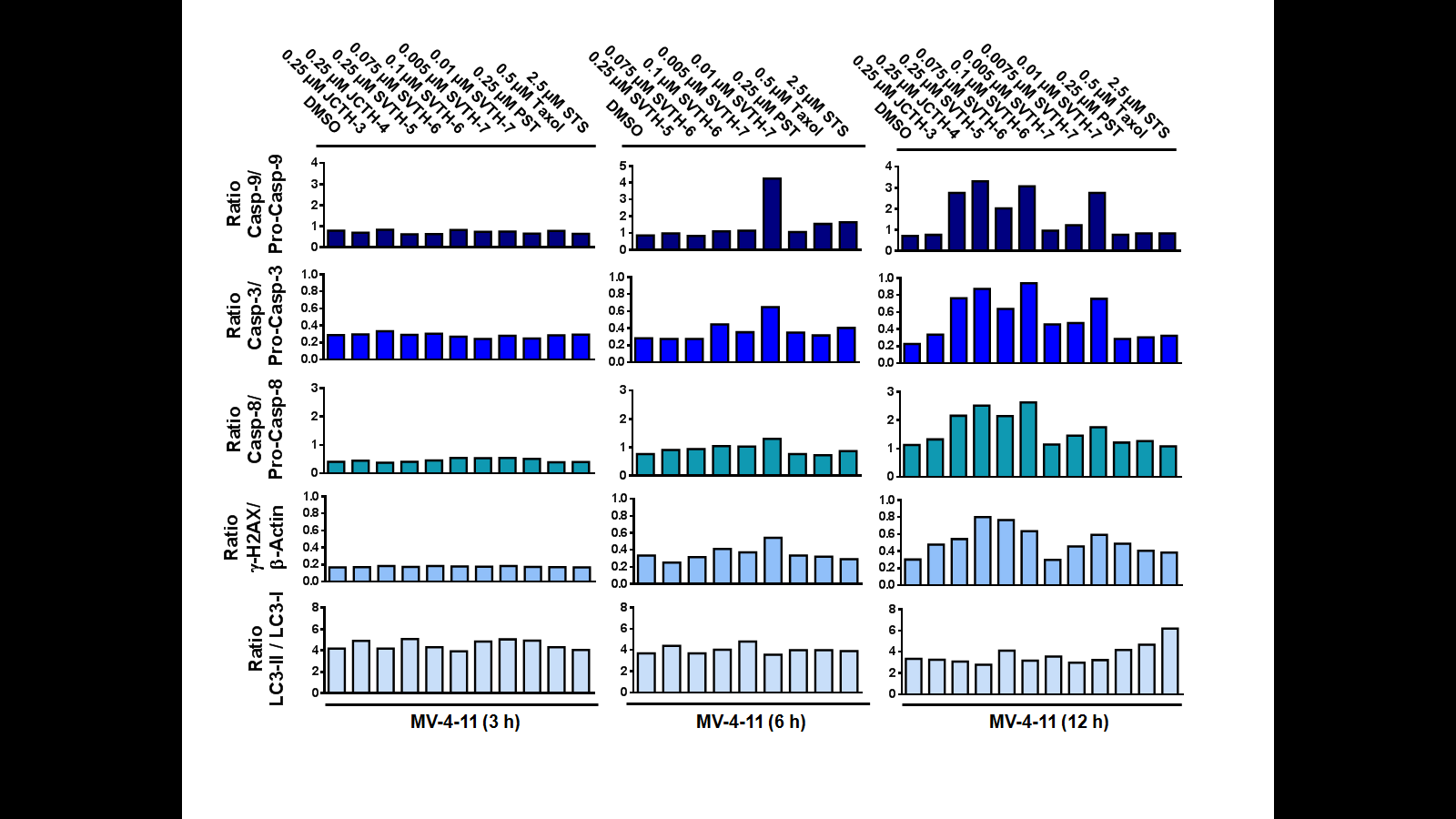


**Supplemental Figure 3a. PST Analogs & PST Induce the Intrinsic Pathway of Apoptosis in MV-4-11 Leukemia Cells: Densitometric Analysis of Western blots from Figure 4A.** Densitometric Analysis was performed on Western blots in Figure 4A of cell lysates of MV-4-11 Leukemia cells treated with PST, PST Analogs, Taxol, and staurosporine (STS) for 3, 6 and 12 hours. All values are representative from at least 3 independent experiments. As observed with 0.01 µM SVTH-7 treatment, Caspase-9 (Casp-9) is prominently activated at 6 hours with some activation of Caspase-3 (Casp-3) and minimal activation of Caspase-8 (Casp-8). Activation of Casp-9/3/8 was observed at 12 hours with treatment of JCTH-4, SVTH-5, SVTH-6, and SVTH-7.


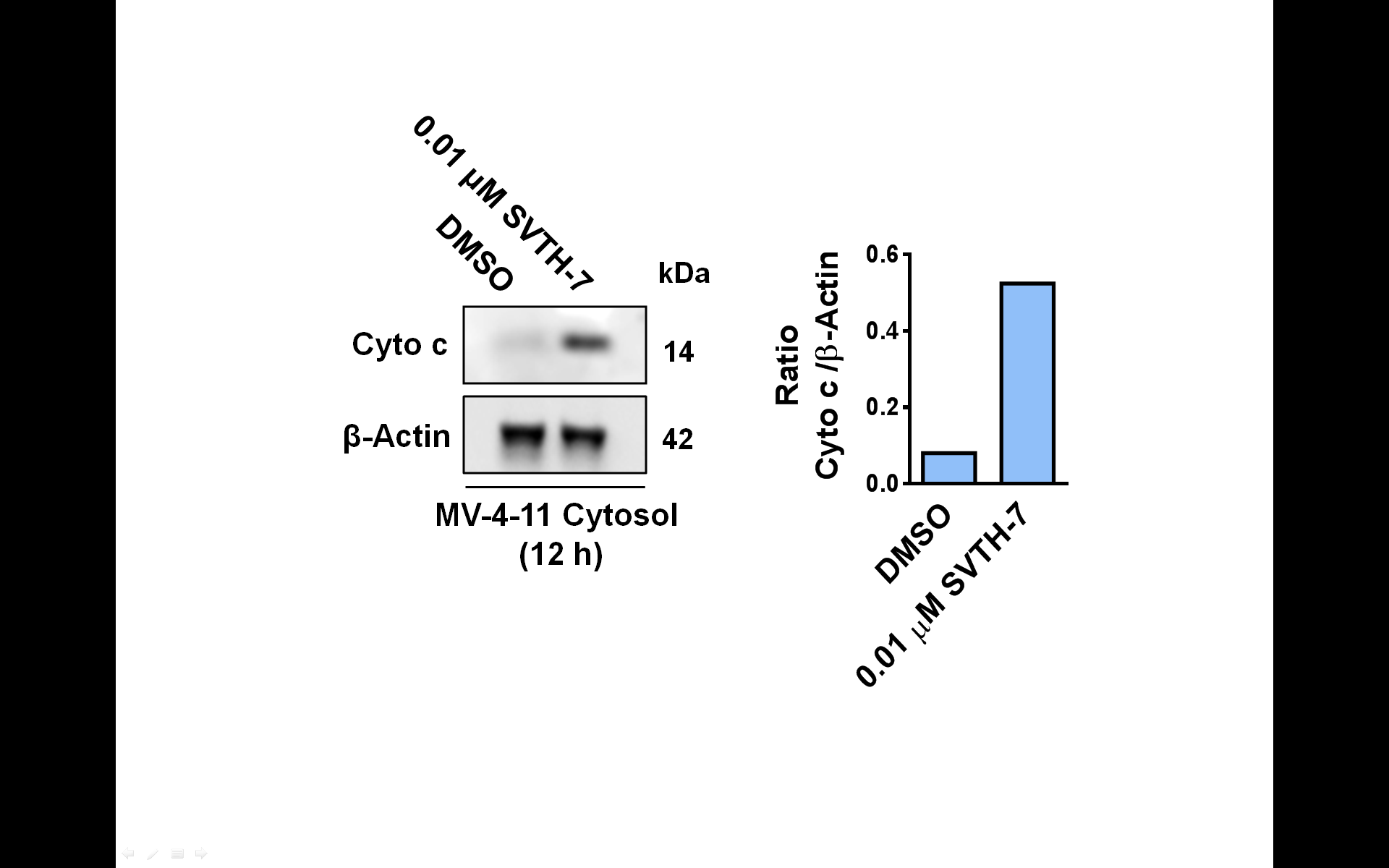


**Supplemental Figure 3b. PST Analog Causes Release of Apoptogenic Factor Cytochrome c (Cyto c) from Mitochondria of MV-4-11 Leukemia Cells.** MV-4-11 leukemia cells were treated with SVTH-7 or DMSO for 12 hours. Cells were lysed with a glass tissue grinder and mitochondrial and cytosolic fractions were separated as described in the materials and methods. Western blot analysis for Cyto c and β-Actin was performed on the cytosolic fractions. Densitometric analysis was performed and the levels of Cyto c were normalized with β-Actin. Images and values are representative of 3 independent experiments demonstrating similar trends.


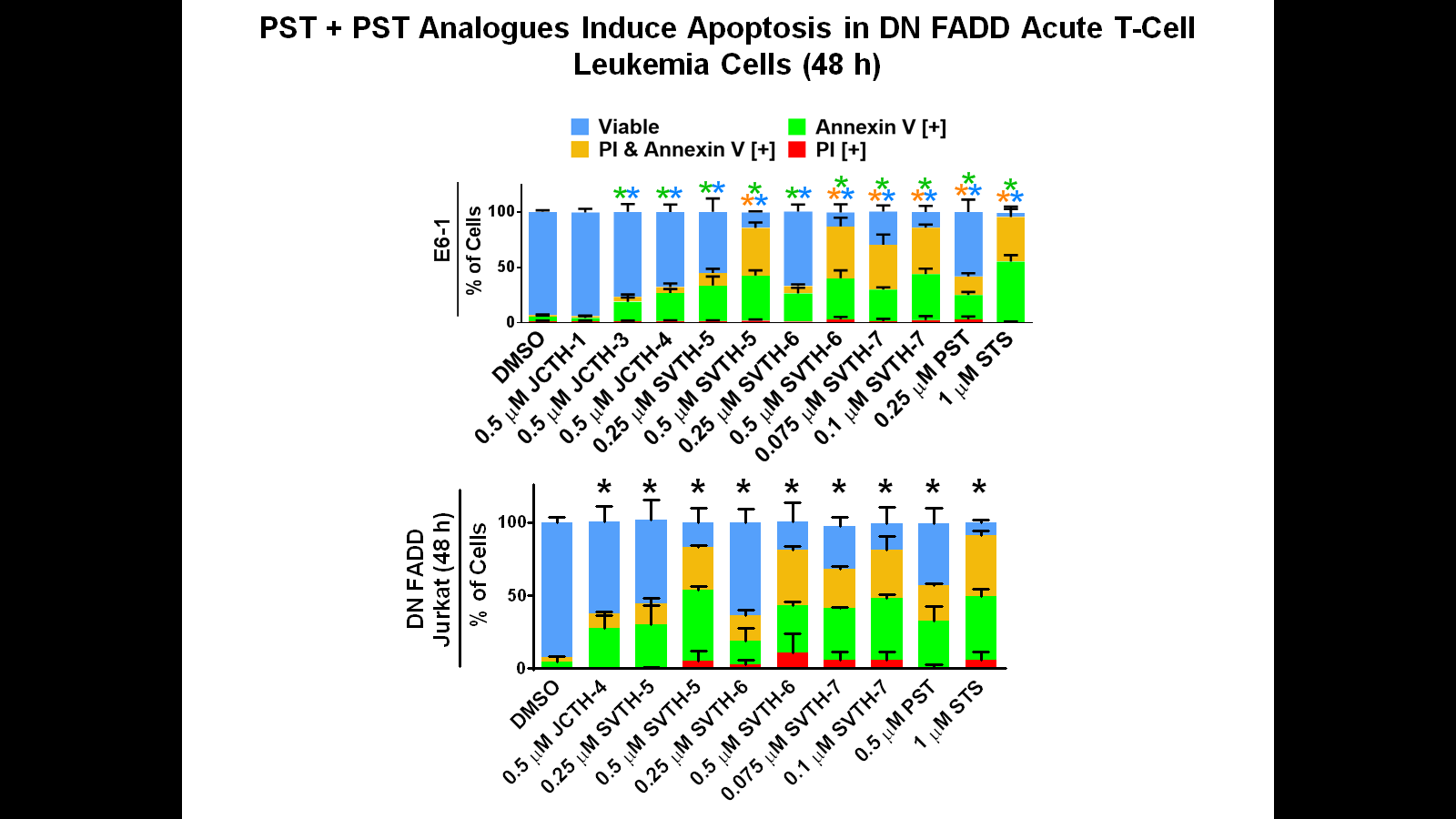


**Supplemental Figure 3c. PST Analog-Induced Cell Death in Leukemia Cells is Not Highly Dependent on the Extrinsic Pathway of Apoptosis.** Jurkat cells (E6-1 leukemia cells) and dominant negative FADD (Fas-Associated Death Domain) jurkat cells (DN FADD Jurkat) were treated with PST analogs and staurosporine (STS) for 48 hours. Annexin V binding and PI staining was monitored with image-based cytometry. Values are expressed as mean ± SD from at least 3 independent experiments. **p*<0.01 vs. DMSO control (comparison of viable cells only for DN FADD Jurkat). No observable difference in the percentage of viable cells was observed between Jurkat and DN FADD Jurkat cells with the PST and PST analog treatment.


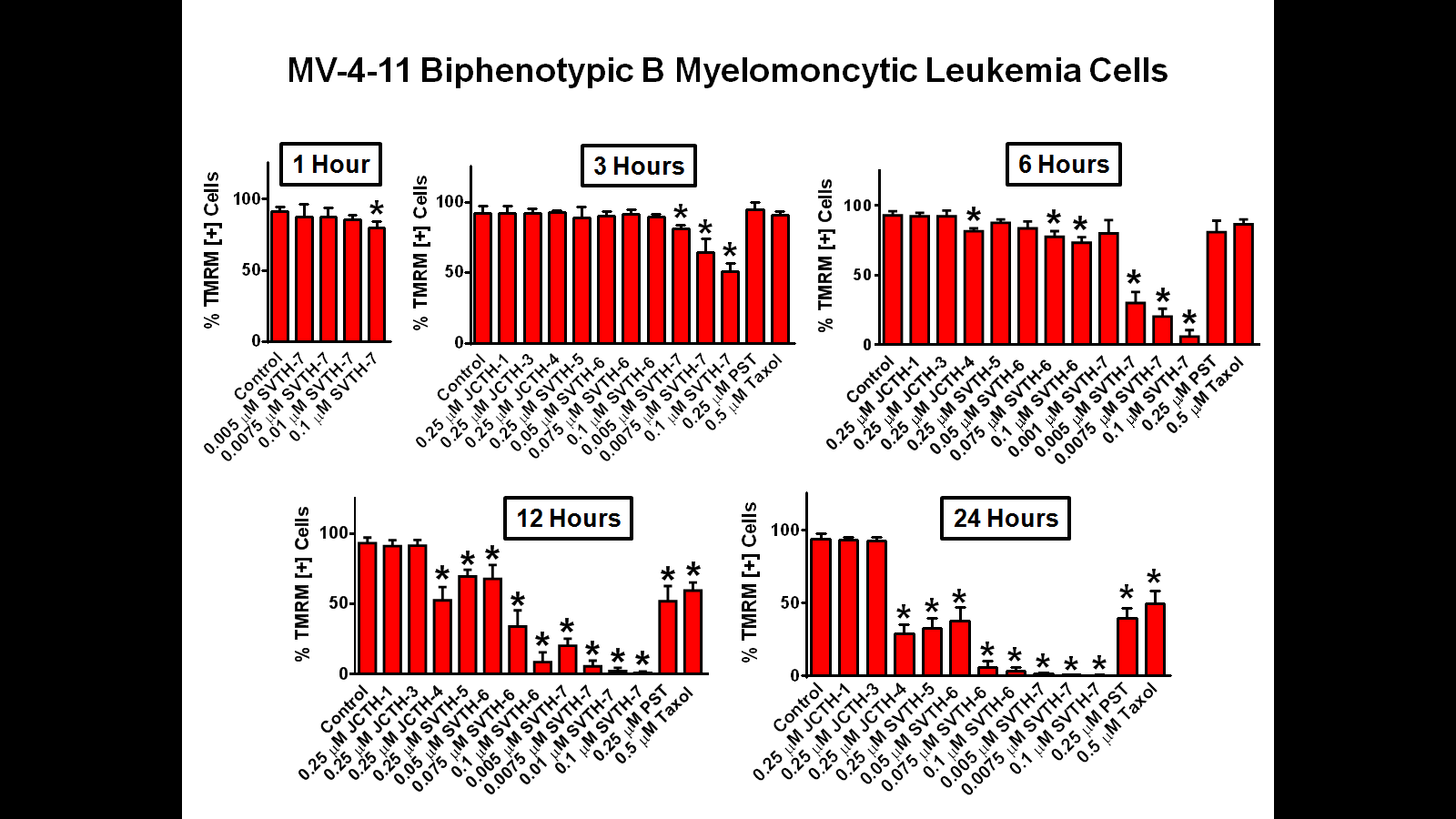


**Supplemental Figure 3d. PST Analogs & PST Cause Mitochondrial Membrane Potential (MMP) Collapse in a Time Dependent Manner in MV-4-11 Leukemia Cells.** TMRM was used to monitor MMP in MV-4-11 leukemia cells with image-based cytometry at the indicated times. **p*<0.01 vs. DMSO control. All quantitative values are expressed as mean ± SD from at least 3 independent experiments. MMP collapse is first observed at 1 and 3 hours with SVTH-7, and at 6 hours with JCTH-4 and SVTH-6. Complete or more pronounced MMP collapse was observed at 12 and 24 hours with JCTH-4 and with SVTH-5, -6, and -7. Taxol was used as a positive control.


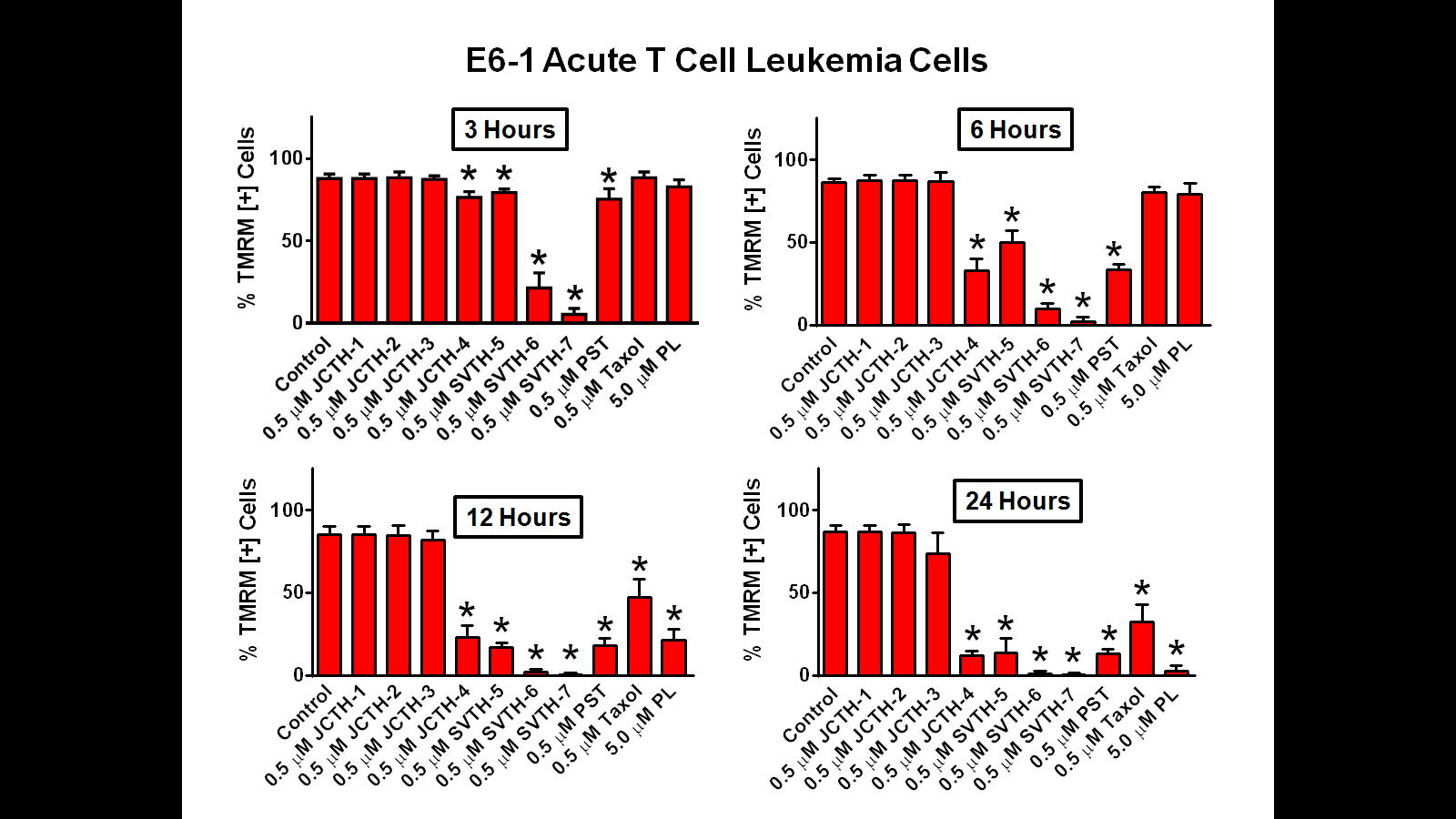


**Supplemental Figure 3e. PST Analogs & PST Cause Mitochondrial Membrane Potential (MMP) Collapse in a Time Dependent Manner in E6-1 Leukemia Cells.** TMRM was used to monitor MMP in E6-1 leukemia cells with image-based cytometry at the indicated times. **p*<0.01 vs. DMSO control. All quantitative values are expressed as mean ± SD from at least 3 independent experiments. MMP collapse is first observed at 3 hours with a more pronounced effect at 6, 12 and 24 hours with JCTH-4 and with SVTH-5, -6, and -7. Taxol and piperlongumine (PL) was used as positive controls.


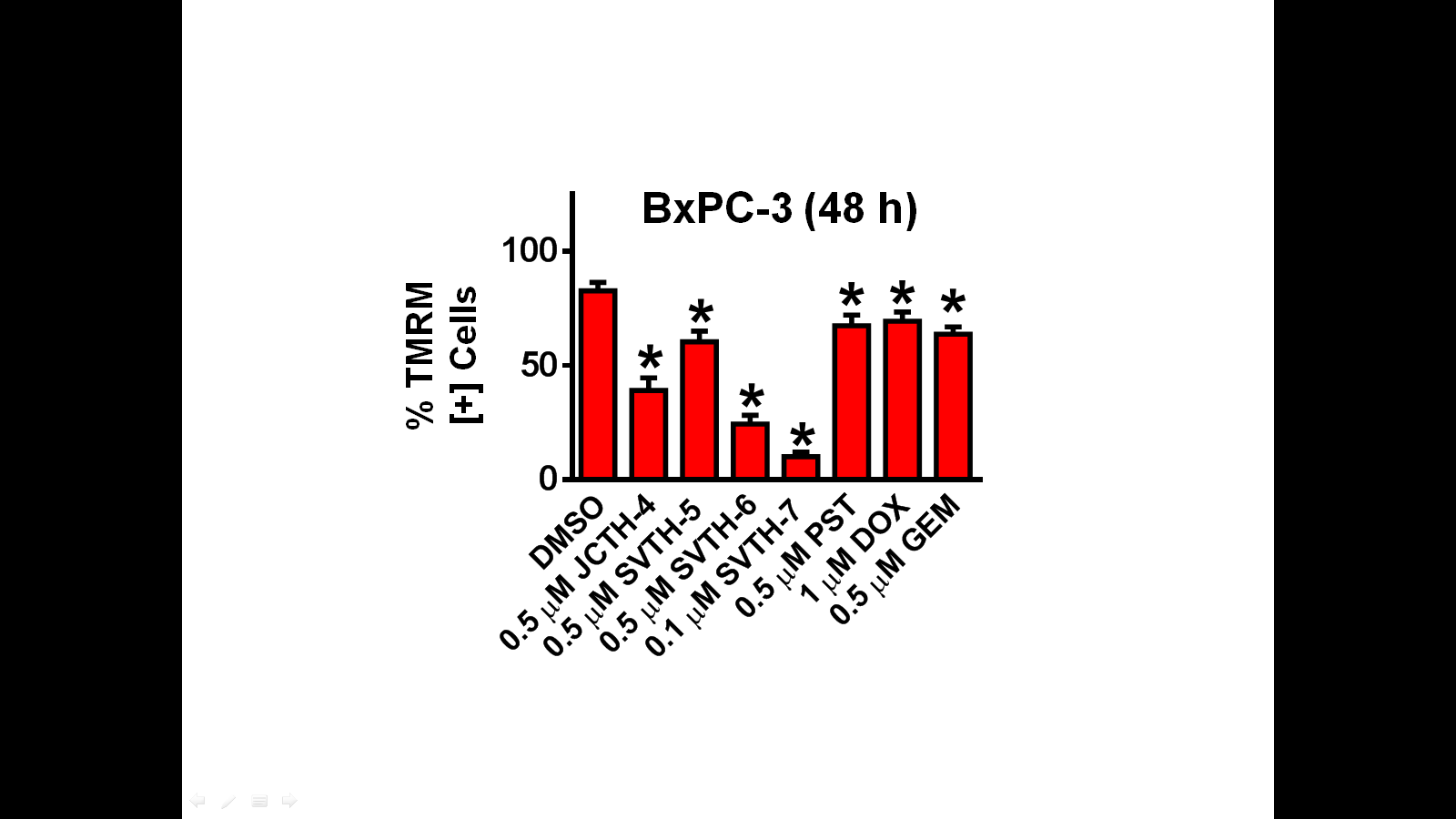


**Supplemental Figure 3f. PST Analogs & PST Cause Mitochondrial Membrane Potential (MMP) Collapse in BxPC-3 Pancreatic Adenocarcinoma Cells.** TMRM was used to monitor MMP in BxPC-3 pancreatic adenocarcinoma cells treated with PST, PST analogs, Doxorubicin (DOX), and Gemcitabine (GEM) with image-based cytometry. **p*<0.01 vs. DMSO control. All quantitative values are expressed as mean ± SD from at least 3 independent experiments.


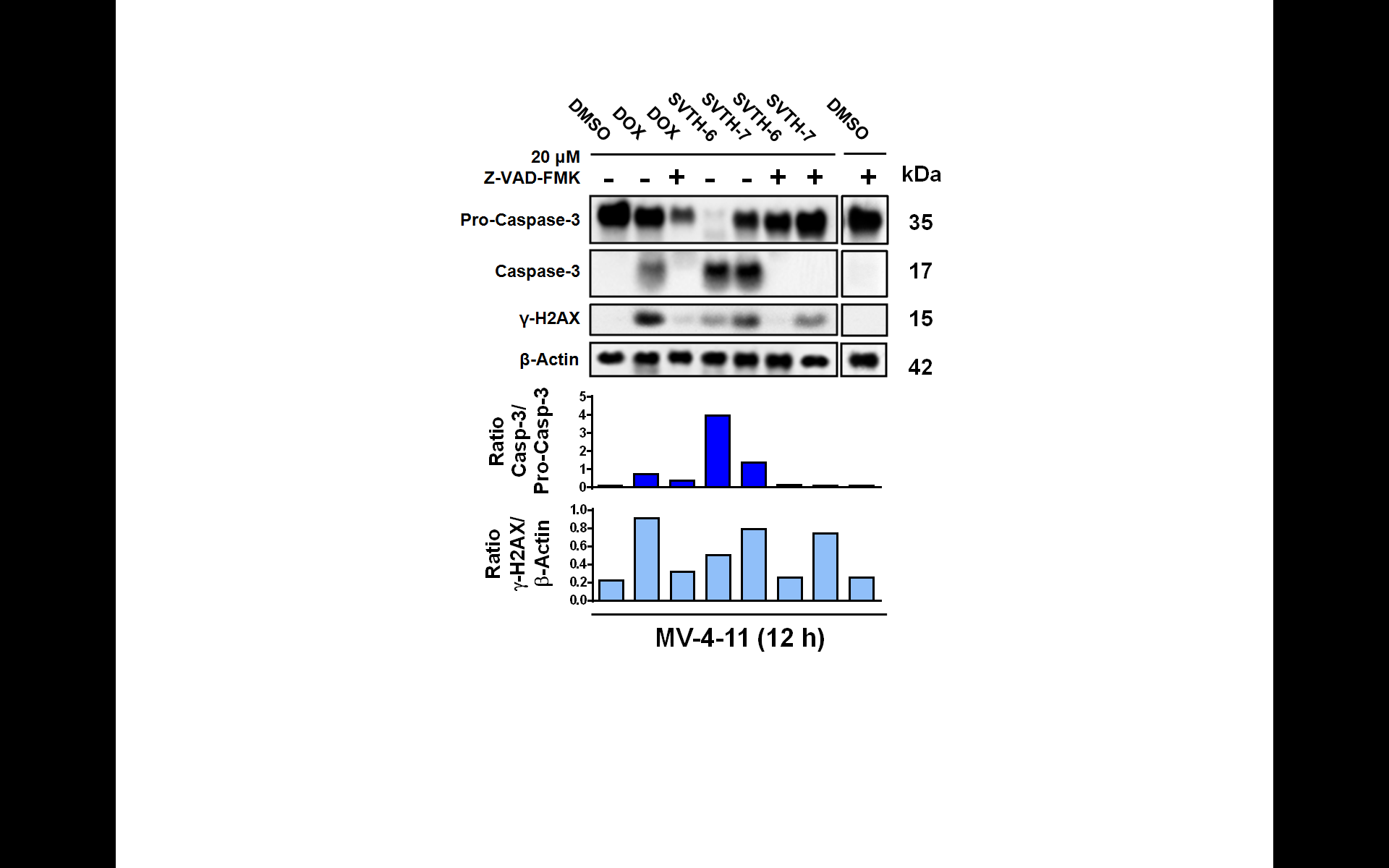


**Supplemental Figure 4a. Z-VAD-FMK Inhibited Casapse-3 cleavage induced by PST Analogs.** MV-4-11 leukemia cells were pre-treated with 30 μM Z-VAD-FMK broad spectrum caspase inhibitor for 1 hour and then treated with PST analogs for 12 hours. Western blots were performed on corresponding cell lysates. Images and values are representative of 3 independent experiments. Z-VAD-FMK was able to prevent Caspase-3 cleavage in cells treated with 0.5 µM SVTH-6, 0.01 µM SVTH-7, and 1 µM Doxorubicin (DOX). Furthermore, Z-VAD-FMK was able to decrease the amount of γ-H2AX, a marker for DNA damage, induced by SVTH-6, SVTH-7 and DOX.


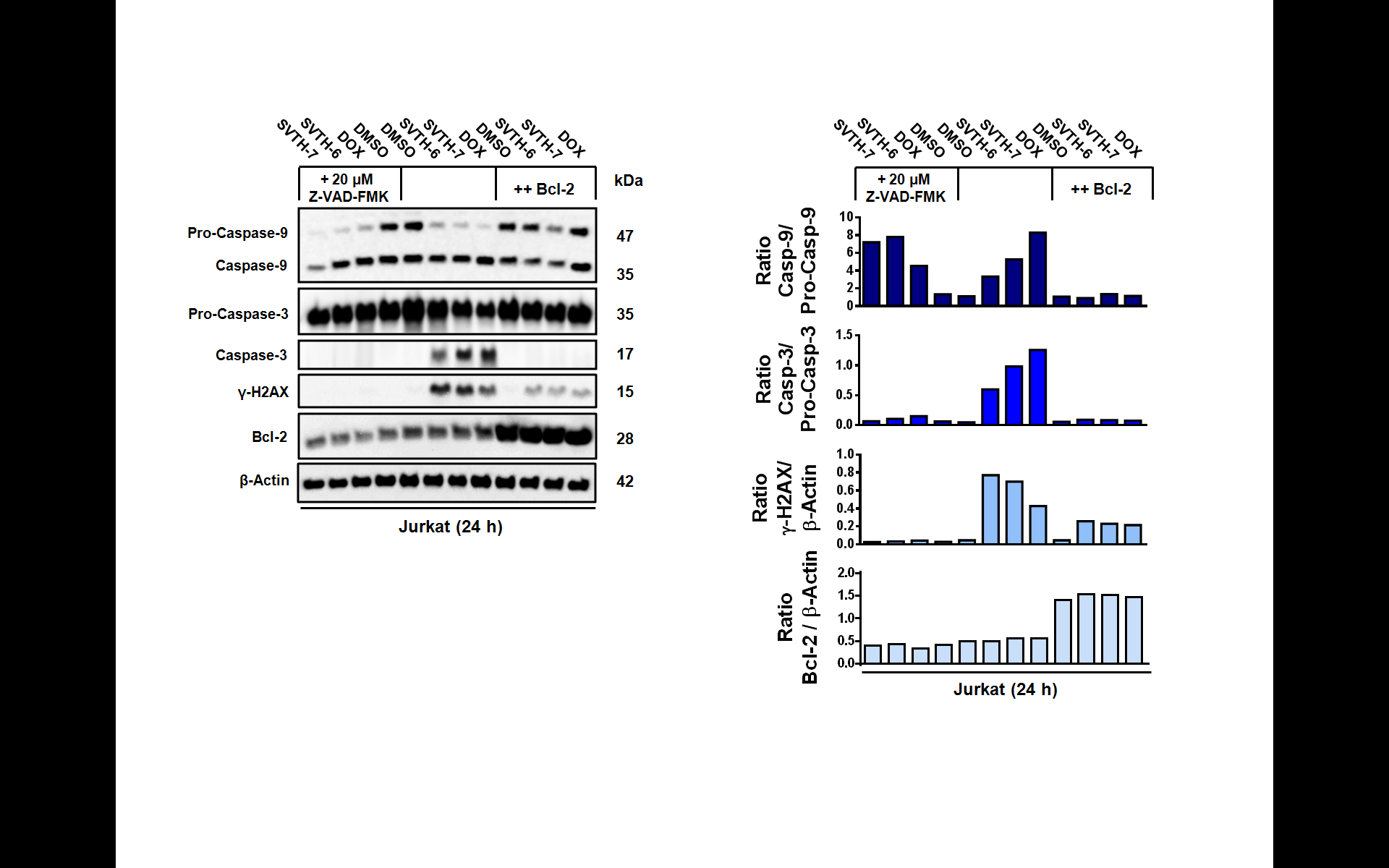


**Supplemental Figure 4b. PST Analog-Induced Apoptosis in Leukemia Cells is Highly Dependent on Mitochondrial Membrane Permeabilization.** Jurkat cells (E6-1 leukemia cells) were pre-treated with 30 μM Z-VAD-FMK broad spectrum caspase inhibitor for 1 hour or DMSO. These cells, along with Jurkat cells over-expressing the anti-apoptotic protein Bcl-2 (++Bcl-2), a protein known to stabilize mitochondria and prevent mitochondrial membrane permeabilization, were then treated with PST analogs and Doxorubicin (DOX) for 24 hours. Western blots were performed on corresponding cell lysates. Images and corresponding densitometric values are representative of 3 independent experiments. Z-VAD-FMK was able to prevent Caspase-3 cleavage and decrease γ-H2AX (marker for DNA damage) in jurkat cells treated with 0.5 µM SVTH-6, 0.1 µM SVTH-7, and 1 µM Doxorubicin (DOX). ++Bcl-2 Jurkat cells had drastically lower levels of Capase-3 and -9 activation and γ-H2AX compared to jurkat cells with no over-expression of Bcl-2.


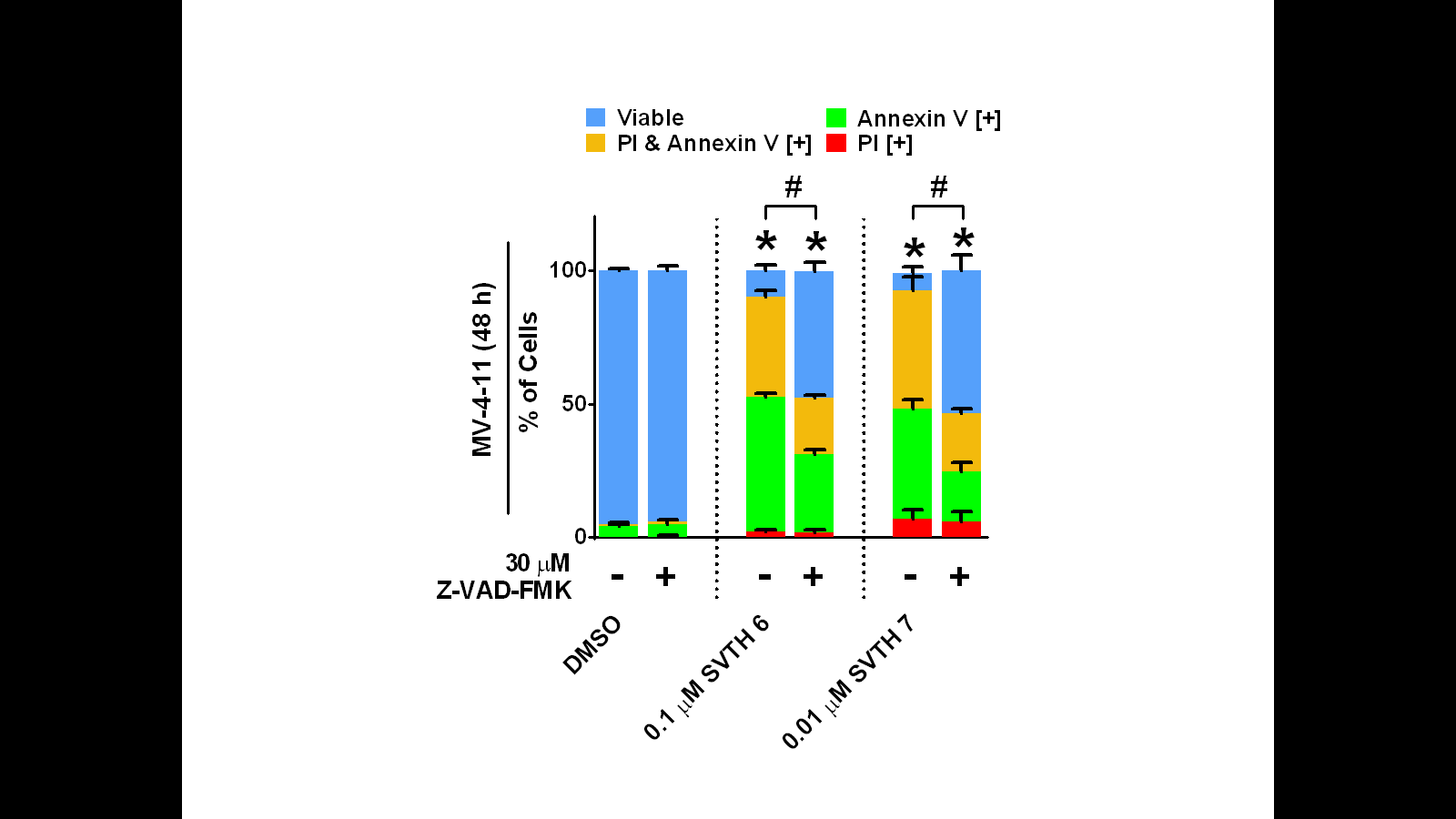


**Supplemental Figure 4c. PST Analogs Induce Apoptosis in MV-4-11 Leukemia Cells in a Partial Caspase-Dependent Manner.** MV-4-11 leukemia cells were pre-treated with 30 μM Z-VAD-FMK broad spectrum caspase inhibitor for 1 hour and then treated with PST analogs to determine the dependence of caspases in PST analog-induced apoptosis. Annexin V binding and PI staining was monitored with image-based cytometry. Values are expressed as mean ± SD from at least 3 independent experiments. **p*<0.01 vs. DMSO control (comparison of viable cells only); #*p*<0.001 vs. respective groups untreated with Z-VAD-FMK (comparison of viable cells only). Z-VAD-FMK was able to partially rescue cells from SVTH-6 and -7, indicating that apoptosis induced by these compounds is partially dependent on caspases.


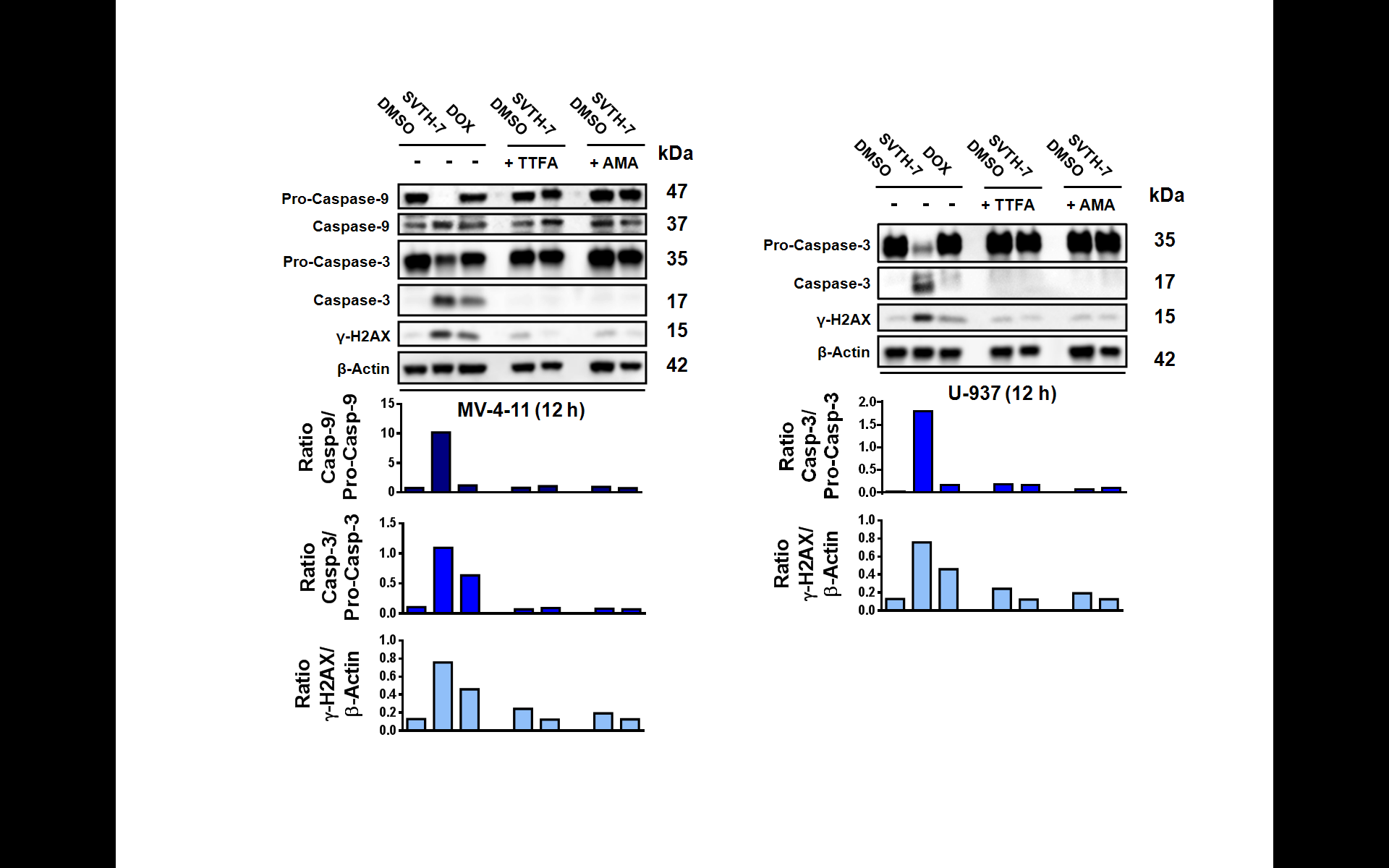


**Figure 5a. PST Analog-Induced Apoptosis is Dependent on Functional Complex II and III of the Mitochondrial Electron Transport Chain.** MV-4-11, and U-937 cancer cells were pre-treated with 25 µM TTFA complex II inhibitor or 5 µM antimycin A (AMA) complex III inhibitor for 1 hour and then treated with PST analog and Doxorubicin (DOX) for 12 hours. Western blots were performed on corresponding cell lysates. Images and densitometric values are representative of 3 independent experiments. TTFA and AMA were able to prevent Caspase-3 and -9 cleavage induced by SVTH-7 treatment and had drastically lower levels of γ-H2AX, a marker of DNA damage.


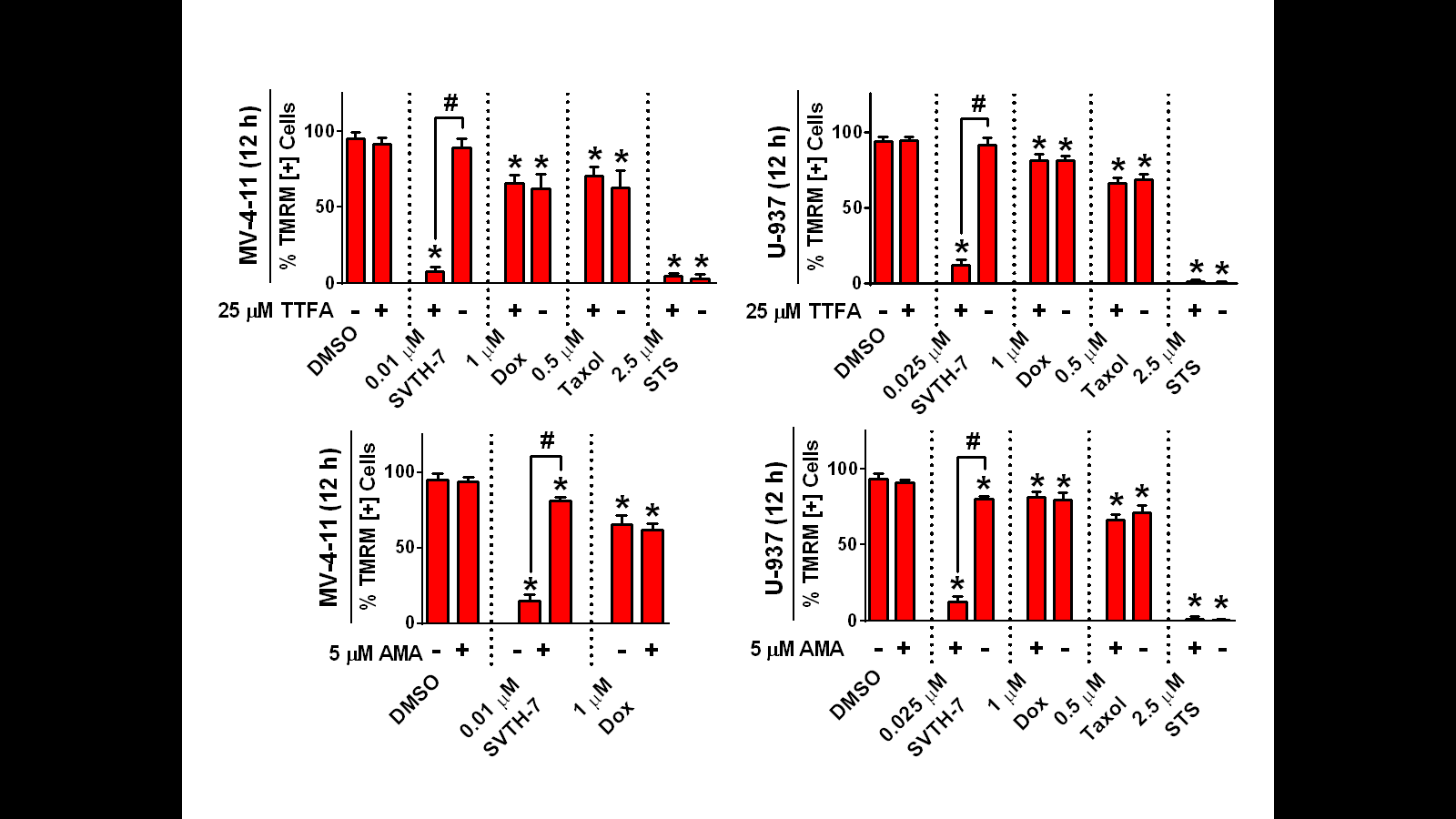
**Supplemental Figure 6. PST Analog-Induced MMP Dissipation is Dependent on Functional Complex II and III of the Mitochondrial Electron Transport Chain.** MV-4-11, and U-937 cancer cells were pre-treated with TTFA complex II inhibitor or antimycin A (AMA) complex III inhibitor for 1 hour and then treated with PST analog SVTH-7, staurosporine (STS), Doxorubicin (DOX), and Taxol for 12 hours. TMRM quantitation of MMP of cells was performed using image based cytometry. **p*<0.01 vs. DMSO control; #*p*<0.001 vs. respective groups without TTFA or AMA. All values are expressed as mean ± SD from at least 3 independent experiments. TTFA and AMA were able to prevent SVTH-7-induced MMP dissipation in MV-4-11 and U-937 cells.


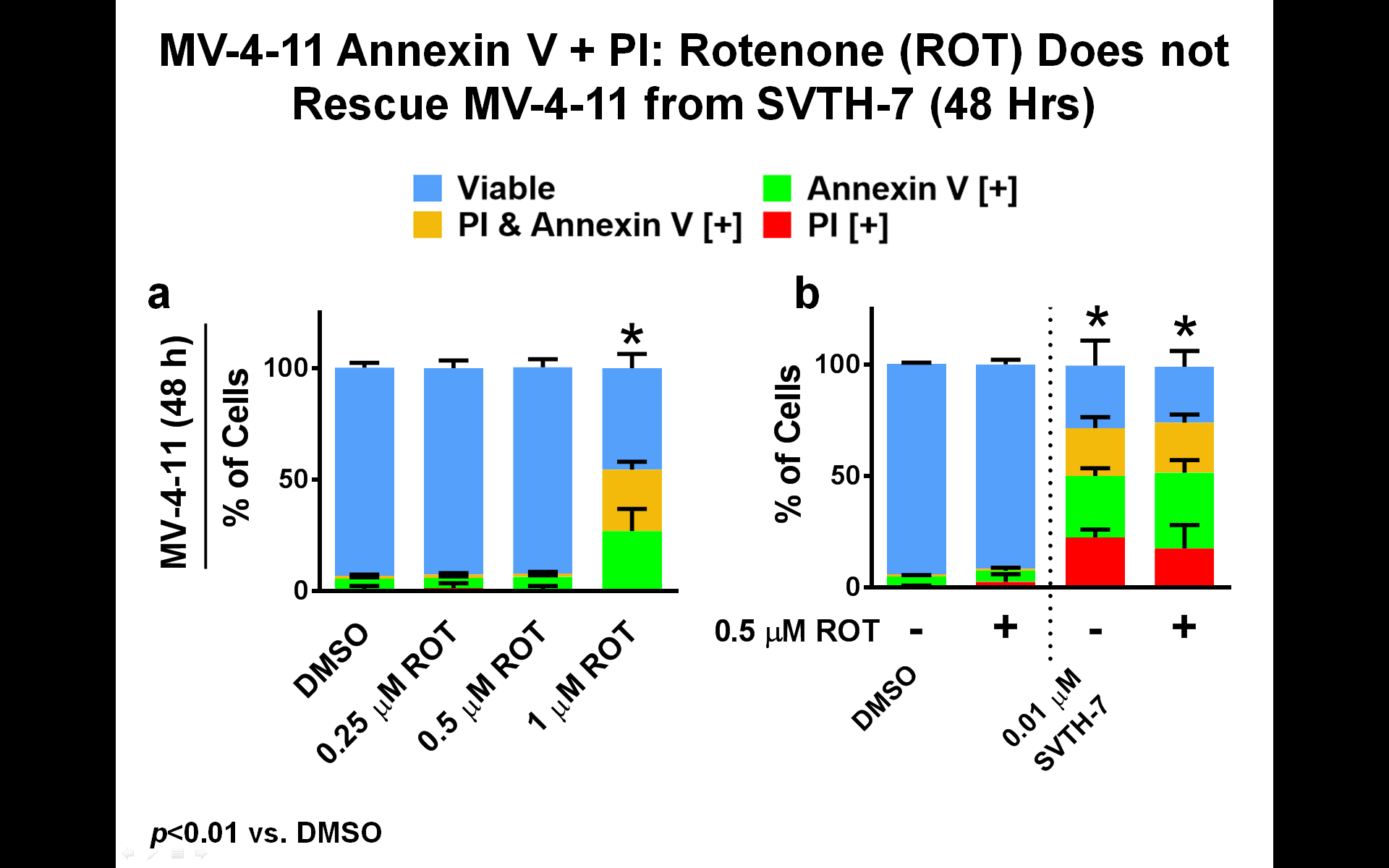


**Supplemental Figure 7. PST Analog-Induced Apoptosis is not Dependent on Functional Complex I of the Mitochondrial Electron Transport Chain. (a)** Different doses of the complex I inhibitor rotenone were tested in MV-4-11 leukemia cells to find a concentration that was well tolerated. **(b)** MV-4-11 leukemia cells were pre-treated with the complex I inhibitor rotenone (ROT) at a well tolerated dose for 1 hour and then treated with PST analog for 48 hours. Annexin V binding (green) and PI staining (PI) (red) was quantified with image based cytometry. **p*<0.01 vs. DMSO control (comparison of viable cells only). All values are expressed as mean ± SD from at least 3 independent experiments. ROT did not appear to affect the apoptosis inducing activity of SVTH-7.


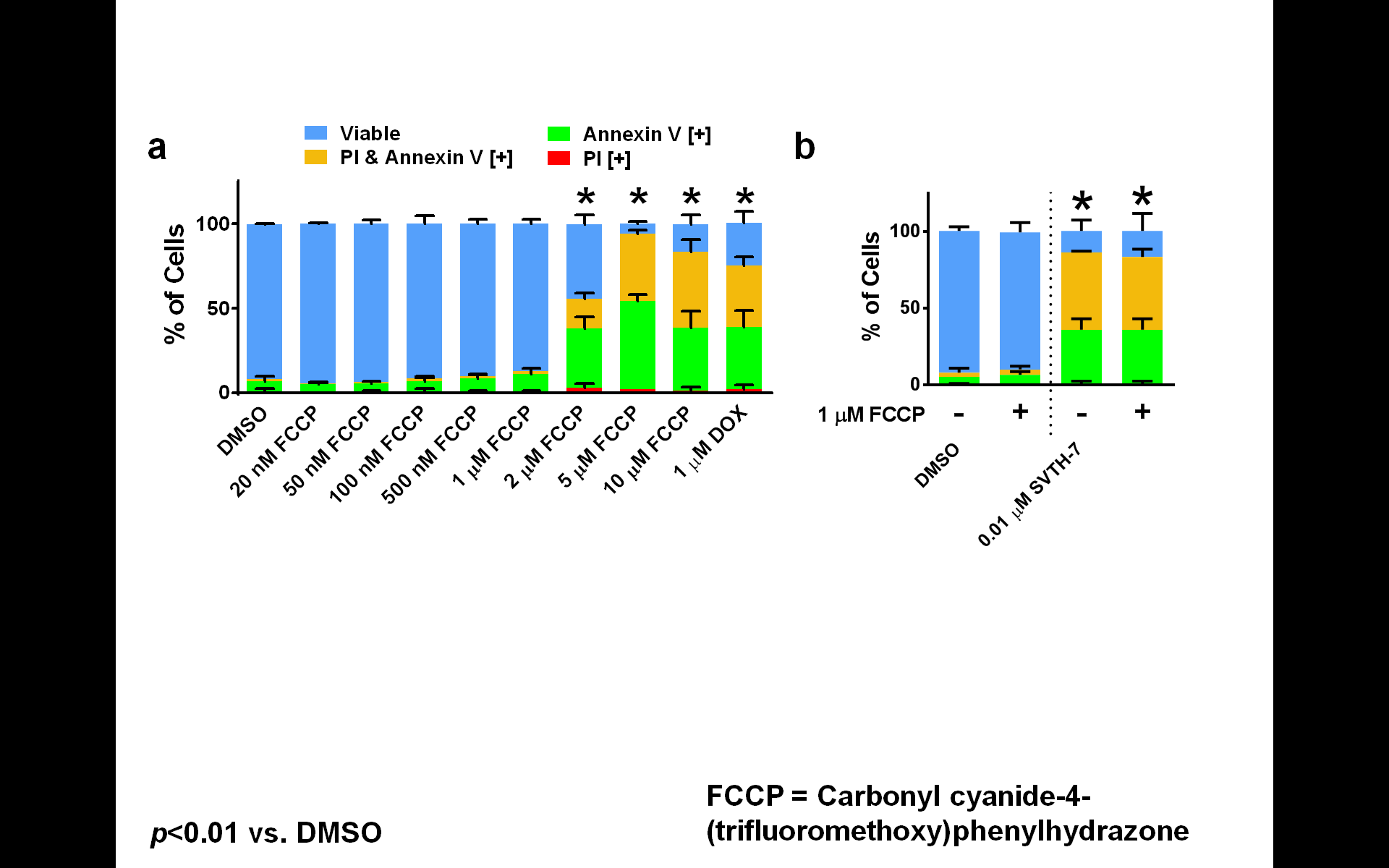


**Supplemental Figure 8. PST Analog-Induced Apoptosis is not Affected by Uncoupling the Electron Transport Chain (ETC) with ATP Production. (a)** Different doses of the carbonyl cyanide-4-(trifluoromethoxy)phenylhydrazone (FCCP) ETC uncoupling agent were tested in MV-4-11 leukemia cells to find a concentration that was well tolerated. **(b)** MV-4-11 leukemia cells were pre-treated with FCCP at a well tolerated dose of 1 µM for 1 hour and then treated with PST analog for 48 hours. Annexin V binding (green) and PI staining (PI) (red) was quantified with image based cytometry. **p*<0.01 vs. DMSO control (comparison of viable cells only). All values are expressed as mean ± SD from at least 3 independent experiments. FCCP did not appear to affect the apoptosis inducing activity of SVTH-7.


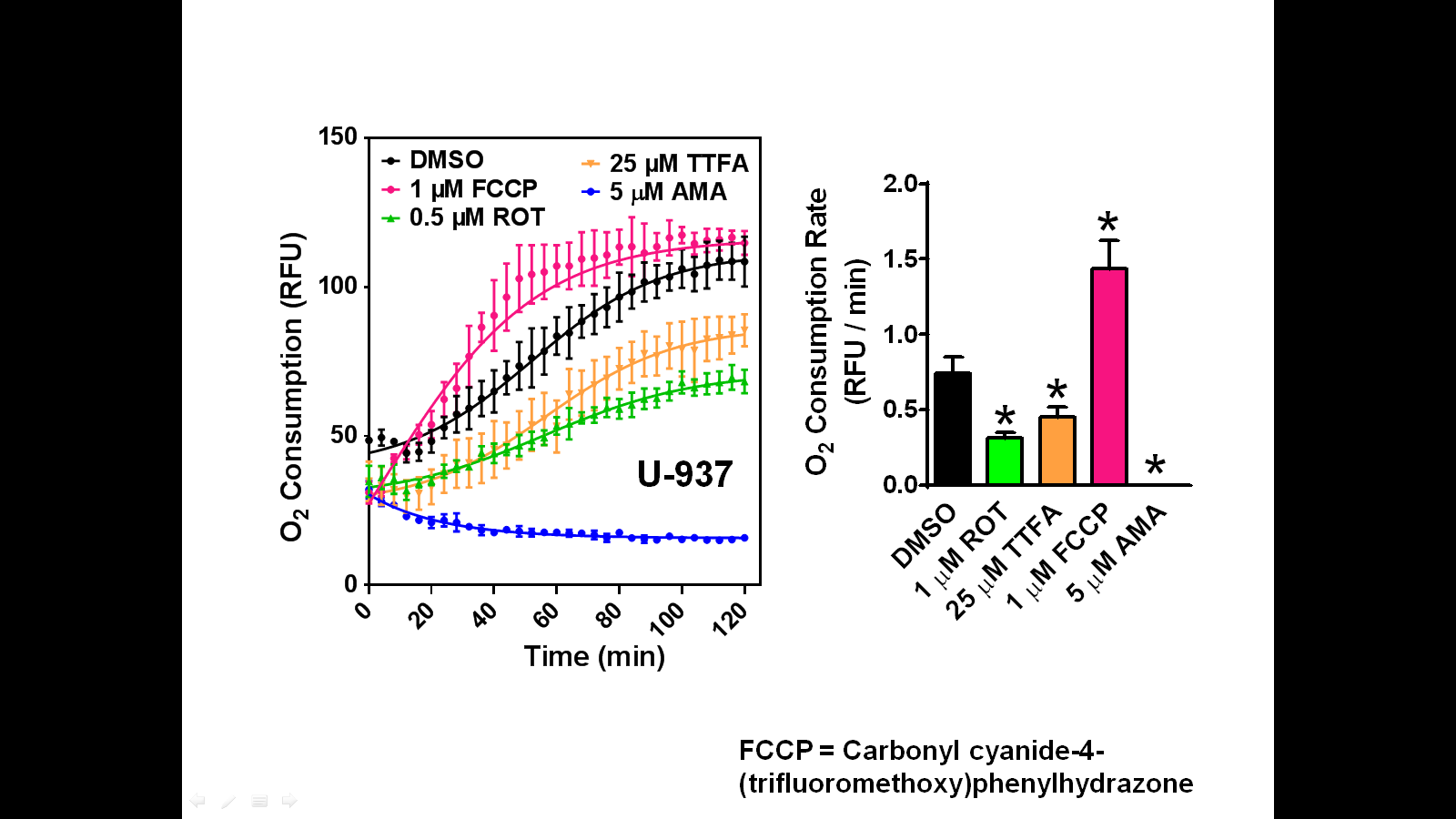


**Supplemental Figure 9. Electron Transport Chain (ETC) Modulators Affect Mitochondrial Function.** The MitoXpress® Xtra - Oxygen Consumption Assay was used to monitor oxygen consumption via fluorescence generation as an indicator of mitochondrial function. U-937 lymphoma cells were treated with Rotenone (ROT), carbonyl cyanide-4-(trifluoromethoxy)phenylhydrazone (FCCP), TTFA, and AMA and the fluorescent MitoXpress® reagent was added monitored at Ex. 380 nm and Em. 650, every 2 minutes for 2 hours at 37 °C. Oxygen consumption rates were calculated by measuring the slopes of the linear regions of the oxygen consumption curves. Values are expressed as mean ± SD from at least 3 independent experiments. **p*<0.01 vs. DMSO control. FCCP caused an increase in oxygen consumption rates while ROT, TTFA and AMA caused decreases in oxygen consumption rates.


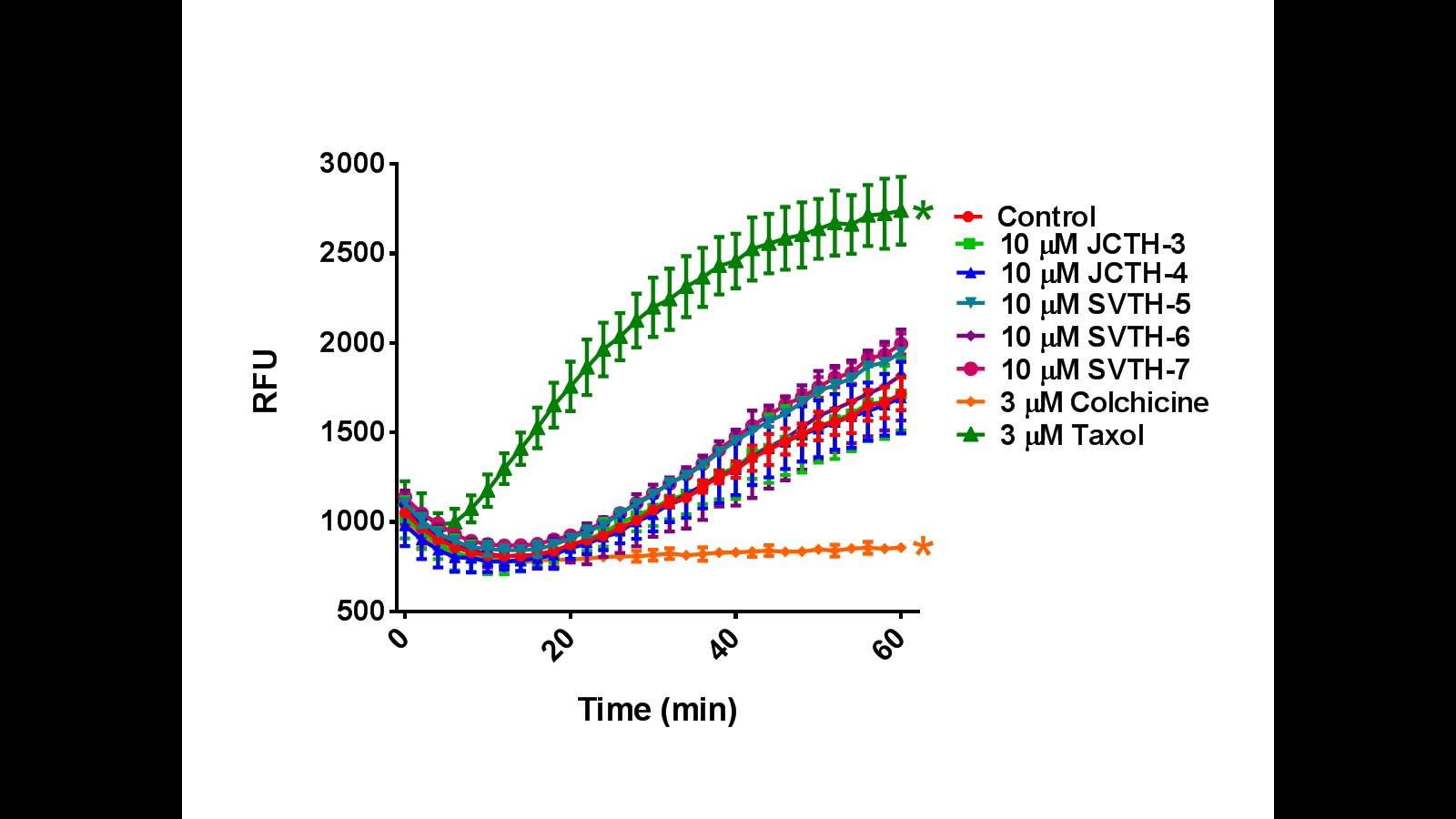


**Supplemental Figure 10. PST Analogs Do Not Evidently Affect Tubulin Polymerization.** A tubulin polymerization assay kit (Cytoskeleton Inc., Cat. No. BK011P, Denver, CO, USA) was used to evaluate PST analogs in their ability to alter tubulin polymerization dynamics. A 96-well plate was pre-warmed at 37°C for half an hour prior to the assay. Wells were treated with DMSO solvent control and PST analogs dissolved in PBS. All wells were incubated with 2 mg/mL tubulin in tubulin polymerization buffer (80 mM PIPES pH 6.9, 2 mM MgCl2, 0.5 mM EGTA, 1 mM GTP and 15% glycerol). Fluorescence was measured (Ex. 360 nm; Em. 450 nm) every 1 minute for 1 hour using a SpectraMax Gemini XS multi-well plate reader (Molecular Devices, Sunnyvale, CA, USA) at 37°C. Fluorescence emission at 450 nm increases as tubulin polymerizes due to the incorporation of a fluorescent reporter. Statistical significance was determined by comparing the relative fluorescence units (RFU) at the 60 minute endpoint between the DMSO solvent control and the compound of interest. All values are expressed as mean ± SD from at least 3 independent experiments. **p*<0.001 vs. DMSO control. Colchicine and Taxol were used as controls for tubulin destabilization and tubulin polymerization respectively.


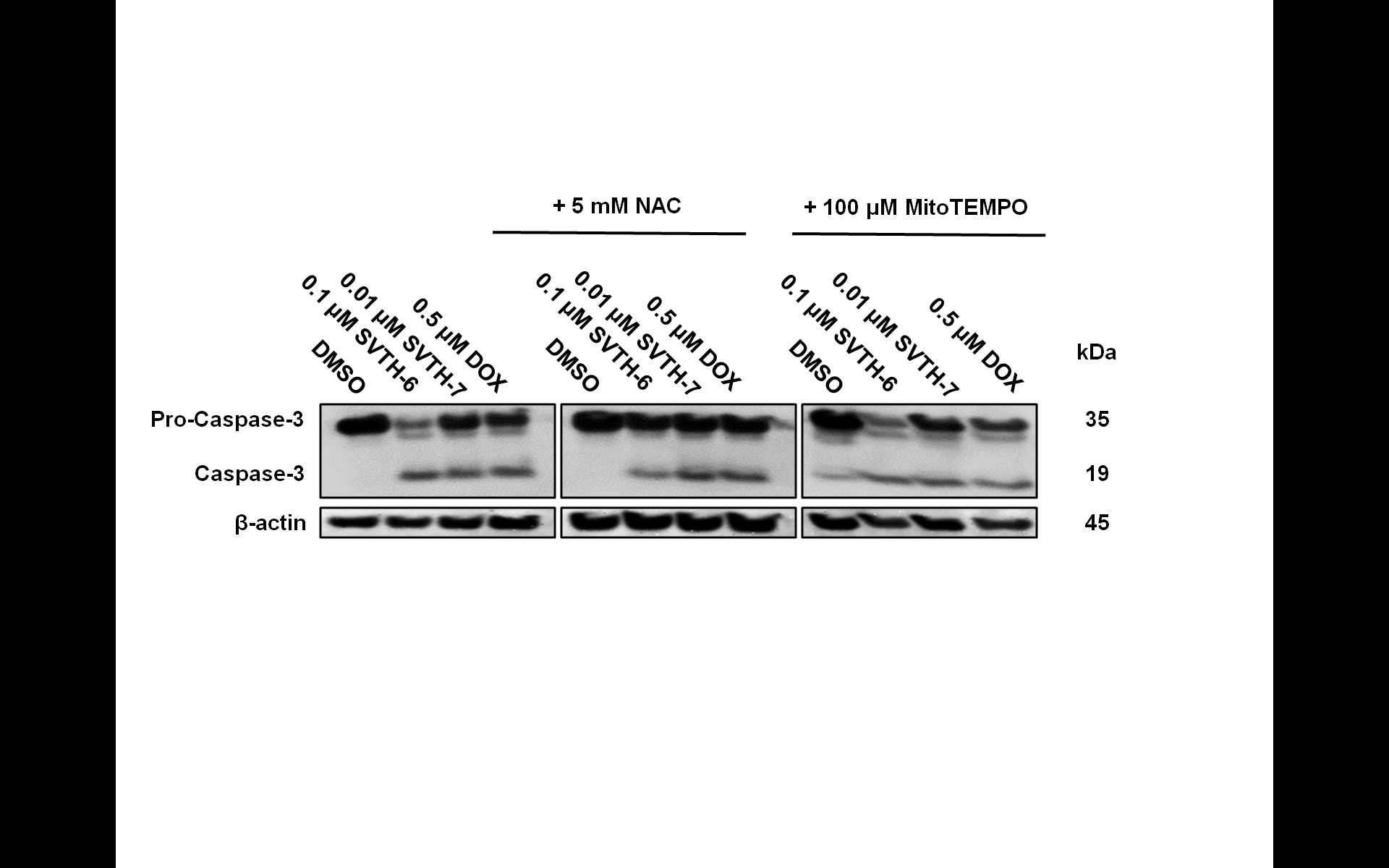


**Supplemental Figure 11. PST Analogues Cause Caspase-3 Cleavage in the Presence of Antioxidants.** The MV-4-11 cell line was treated with DOX, SVTH-6, - and 7 with or without a pre-treatment of NAC and MitoTEMPO for 24 hours. Western blot analysis was carried out and the results are representative of three independent trials.
